# HiHo-AID2: boosting homozygous knock-in efficiency enables robust generation of human auxin-inducible degron cells

**DOI:** 10.1186/s13059-024-03187-w

**Published:** 2024-02-26

**Authors:** Shiqian Li, Yafei Wang, Miesje van der Stoel, Xin Zhou, Shrinidhi Madhusudan, Kristiina Kanerva, Van Dien Nguyen, Nazli Eskici, Vesa M. Olkkonen, You Zhou, Taneli Raivio, Elina Ikonen

**Affiliations:** 1 Department of Anatomy and Stem Cells and Metabolism Research Program, Faculty of Medicine, University of Helsinki, 00290 Helsinki, Finland; 2 Minerva Foundation Institute for Medical Research, 00290 Helsinki, Finland; 3 Stem Cells and Metabolism Research Program, Research Programs Unit, and Department of Physiology, Faculty of Medicine, University of Helsinki, 00290 Helsinki, Finland; 4 Systems Immunity Research Institute, Cardiff University, Cardiff, CF14 4XN UK; 5 Division of Infection and Immunity, School of Medicine, Cardiff University, Cardiff, CF14 4XN UK; 6 New Children’s Hospital, Pediatric Research Center, Helsinki University Hospital, 00290 Helsinki, Finland

**Keywords:** Auxin-inducible degron 2, Homology-directed repair enhancers, Homozygous knock-in, Functional inactivation, Chemogenetics, Human embryonic stem cell (hESC), hESC-derived neurons

## Abstract

Recent developments in auxin-inducible degron (AID) technology have increased its popularity for chemogenetic control of proteolysis. However, generation of human AID cell lines is challenging, especially in human embryonic stem cells (hESCs). Here, we develop HiHo-AID2, a streamlined procedure for rapid, one-step generation of human cancer and hESC lines with high homozygous degron-tagging efficiency based on an optimized AID2 system and homology-directed repair enhancers. We demonstrate its application for rapid and inducible functional inactivation of twelve endogenous target proteins in five cell lines, including targets with diverse expression levels and functions in hESCs and cells differentiated from hESCs.

**Supplementary Information:**

The online version contains supplementary material available at 10.1186/s13059-024-03187-w.

## Background

The auxin-inducible degradation (AID) technology allows chemogenetic control of proteolysis [1]. To apply AID, a destabilizing peptide, or “degron,” is tagged to the target protein by genetic engineering. An auxin receptor (such as *Os*TIR1) is exogenously expressed in the same cells, functioning as the substrate recognizing subunit of Skp1-Cullin1-TIR1 (SCF^TIR1^) ubiquitin ligase complex. Auxin (such as Indole-3-acetic acid, IAA) bridges the SCF^TIR1^ ubiquitin ligase and the degron-tagged protein as a chemical glue, leading to rapid poly-ubiquitination and proteasomal degradation of the degron-tagged protein [1, 2]. AID shows rapid and efficient targeted protein degradation, avoids secondary and side effects observed during long-term silencing or CRISPR knock-out, and has provided important mechanistic insights into the functions of diverse target proteins in dynamic biological processes [3–7]. However, some hurdles have limited our ability to harness the full potential of AID.

The first challenge is to engineer optimal AID components. The initial AID systems in mammalian cells using the auxin receptor *Os*TIR1 resulted in severe degradation of target proteins before induction, known as basal or leaky degradation [8–10]. Several strategies have recently been developed to tackle this issue. We found that substitution of *Os*TIR1 by its homolog *At*AFB2 largely avoided basal degradation [8]. Moreover, *Os*TIR1(F74G) mutant that exploits a bump-and-hole approach developed in plant showed no leaky degradation [9, 11]. Other AID components have also been tailored, to enable efficient inducible degradation with low inducer concentration. Substituting the degron miniAID by miniIAA7 improved the inefficient inducible degradation with *At*AFB2 and changing the inducer IAA to 5-PH-IAA required a 670 times lower ligand concentration with *Os*TIR1(F74G) [8, 9]. The *Os*TIR1(F74G)/5-PH-IAA combination was designated as the first AID2 system that features non-leaky degradation and low inducer concentration [9]. The low concentration of 5-PH-IAA significantly improved the efficiency of AID in mice [9]. Similar to 5-PH-IAA, cvxIAA and pico_cvxIAA were used at 100- to > 1000-fold lower concentrations than IAA with both *Os*TIR1(F74G) and *Os*TIR1(F74A) mutants [12, 13]. These developments have solved technical pitfalls of AID components, with the AID2 system appearing as the most promising one for future applications.

**Fig. 1:**
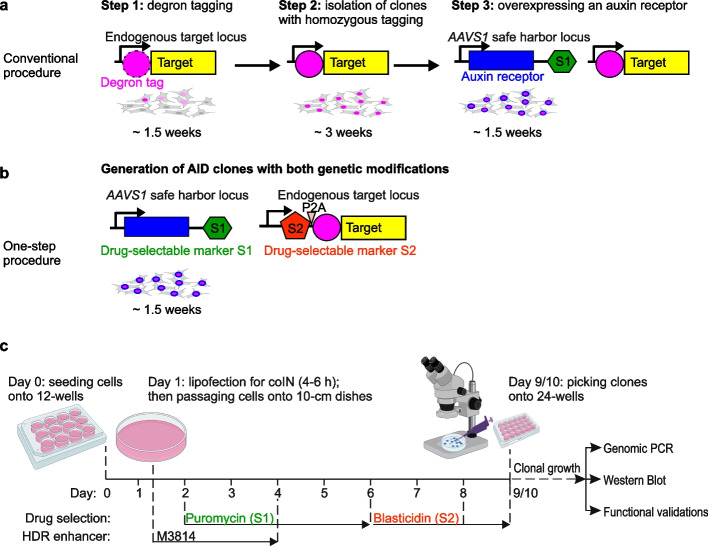
Overview of conventional and HiHo-AID2 procedures to generate AID cells. **a**, **b** Illustration of genetic modifications and time required to generate AID clones with conventional (**a**) and HiHo-AID2 (**b**) procedure. P2A: self-cleaving peptide. **c** Timeline for generation of AID clones in human cell lines with HiHo-AID2. Dashed line: timepoints for medium change or passaging/picking of cells

The second challenge is to effectively generate AID cells. Engineering cells with AID to deplete endogenous proteins requires two genetic modifications, i.e., homozygous degron tagging and auxin receptor expression. With the ease of CRISPR-Cas9 for genetic engineering, applications of AID in mammalian cells have become feasible [8, 10]. However, homozygous degron tagging through CRISPR/Cas9-mediated homology-directed repair (HDR) suffers from low efficiency in mammalian cells [14]. Hence, FACS sorting or drug selection is first used to enrich the engineered cells before single-cell cloning, and additional engineering steps are required to introduce the auxin receptor (Fig. 1a) [8, 10]. Such multi-step procedures are challenging to establish, labor-intensive, time consuming, and cannot be easily scaled up for multiple targets. Moreover, homozygous tagging in pluripotent cells, such as human embryonic stem cells (hESCs), suffers from even lower efficiency, and AID has so far not been documented in any hESC-derived cell lineage [15–18].

Recently, several techniques that improve HDR efficiency and/or enrich HDR in cells have been developed. These include the use of HDR enhancers [19–22], special design of HDR template [23, 24], 2A-drug selection cassette (2A: self-cleaving peptide) [25–27], and co-incident insertion (coIN, or coselection) that allows introduction of two genetic modifications in one step [28, 29]. In this work, we developed HiHo-AID2, a robust one-step procedure that integrates optimized components of the *AID2* system with the use of coIN and HDR enhancers to achieve *hi*ghly efficient generation of AID cell lines with *ho*mozygous degron tagging and auxin receptor expression. HiHo-AID2 is scalable for multiple targets and applicable for several human cancer cell lines as well as hESCs. The resulting hESC lines were further differentiated into embryoid bodies (EBs) and neurons, where we achieved chemogenetic control of several functionally distinct proteins with robust anticipated phenotypic outcomes.

## Results

### Overview of HiHo-AID2

Conventionally, AID cells are generated with low-efficiency homozygous tagging and require two engineering steps (Fig. 1a) [8, 10]. To simplify and boost the process, we took advantage of coIN and HDR enhancers to increase homozygous degron-tagging efficiency. In addition, coordinated selection with puromycin (S1) and blasticidin (S2) was used to simultaneously enrich AID cells with both genetic modifications, i.e., degron tagging and auxin receptor expression (Fig. 1b, c). Afterwards, manual picking under a stereo microscope enabled simple isolation of AID cell clones from 10-cm dishes (Fig. 1c). Overall, the procedure is robust and efficient and does not require other special equipment. It takes about 10 days from initial cell seeding to clone isolation and only a small number of clones (typically 6–10) need to be screened. Simultaneous handling of 10–20 plates is feasible by a single operator. Below we describe the development of HiHo-AID2.

We first compared different options of the AID components, i.e., auxin receptor, degron tag, and chemical inducer [8, 9, 13]. We chose an AID system composed of *At*AFB2(F74A) as the auxin receptor, miniIAA7-3xFlag as the degron tag, and 0.5 µM pico_cvxIAA as the inducer of proteolysis. This modified AID2 system uses a small degron tag, shows no basal degradation, and achieves rapid inducible depletion with negligible off-target effects of the inducer (Additional file 1: Fig. S1, and Note S1).

For efficient coIN, plasmids at a ratio of 1:3 (*AAVS1*: endogenous locus) were chosen to simultaneously introduce *At*AFB2(F74A) through HDR-mediated *AAVS1* safe harbor integration and tag endogenous loci with a degron (Additional file 1: Fig. S2, and Note S2). Testing of several HDR enhancers in coIN identified M3814 [19, 23] and i53 [20] as effective HDR enhancers that increased degron-GFP tagging efficiencies (Additional file 1: Fig. S3a-b, and Note S2). M3814 and i53 inhibit DNA-PK and 53BP1 respectively, both of which are pro-NHEJ factors limiting the efficiencies of HDR [14]. Nearly 100% of cells were *At*AFB2(F74A)-mCherry positive after puromycin selection in all experiments unless otherwise specified (Additional file 1: Fig. S2b).

### HDR enhancers and coIN synergize to improve degron-tagging efficiency

**Fig. 2:**
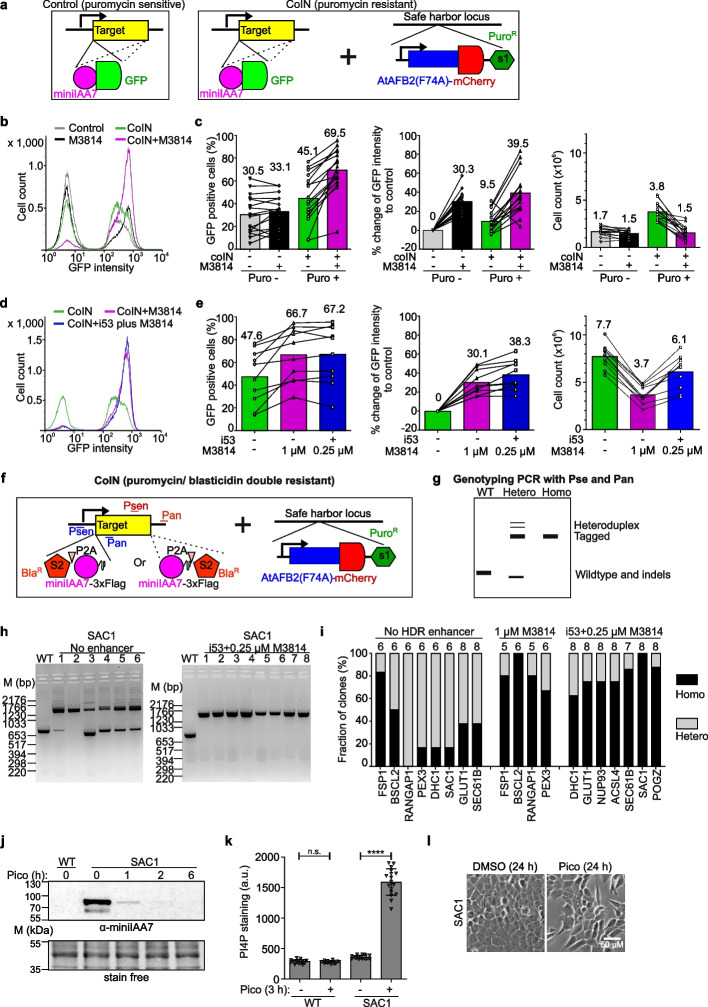
Establishment and application of HiHo-AID2 in A431 cells. **a** Scheme of genetic modifications for degron-GFP tagging through conventional procedure (control) and one-step procedure (coIN) to assess tagging efficiencies in **b**–**e**. PuroR: puromycin-resistance gene. **b**, **c** Comparison of degron-GFP tagging efficiencies in conventional procedure and one-step procedure with 1 μM M3814 as HDR enhancer as indicated. A representative FACS profile (**b**) and statistics of percentage of GFP-positive cells (**c**, left), the percentual change of single-cell GFP intensity compared to control (conventional procedure) (**c**, middle), and the cell count (× 10^6^ cells/ml) (**c**, right) are shown. *N* = 16 pairs of tagging plasmids. Statistical comparisons are shown in Additional file 4: Table S6. **d**, **e** Comparison of degron-GFP tagging efficiencies in one-step procedure using different HDR enhancers. A representative FACS profile (**d**) and graphs depicting the percentage of GFP-positive cells (**e**, left), the percentual change of single-cell GFP intensity to control (coIN) (**e**, middle), and the cell count (× 10^6^ cells/ml) (**e**, right) are shown. *N* = 10 pairs of tagging plasmids. Numbers above columns indicate mean values; lines link the same endogenous tagging pairs in **c**, **e**. Statistical comparisons are shown in Additional file 4: Table S6. **f** Scheme of the genomic modifications in HiHo-AID2. BSD: Blasticidin S deaminase. Psen and Pan indicate the primer set for genotyping PCR of either N- or C-terminal tagging. **g** Scheme of a representative genotyping PCR result showing heterozygous and homozygous tagging at endogenous loci in the AID clones. **h** Genotyping PCR results of SAC1 clones generated with or without i53 plus 0.25 μM M3814 as HDR enhancers. **i** Graphs depicting the genotyping PCR results for 8 targets without HDR enhancer, and 11 targets with either 1 μM M3814 or i53 plus 0.25 μM M3814 as HDR enhancers. Numbers above columns indicate total amount of clones analyzed. **j** WB analysis of inducible SAC1 degradation. **k** Graph showing the PI4P staining intensity upon SAC1 degradation in A431 wild-type and SAC1 AID cells. One-way ANOVA, n.s.: non-significant, ****p < 0.001. *N* = 17 fields. All statistical comparisons are shown in Additional file 4: Table S6. **l** Widefield imaging of cell morphological changes upon SAC1 degradation. Scale bar: 50 μM. Representative of 2 clones (**j**, **k**). WT: wild-type; Hetero: heterozygous; Homo: homozygous; pico: 0.5 μM pico_cvxIAA treatment; a.u. arbitrary unit

We next tested 16 endogenous degron-GFP tagging pairs (8 templates with 2 different sgRNAs each) to assess the degron-tagging efficiencies through combining coIN and HDR enhancer M3814 (1 µM) in A431 cells. While coIN increased the percentage of GFP-positive cells by 1.5-fold (from ~ 30 to ~ 45%), addition of M3814 raised average GFP intensities by about 30% in GFP-positive populations (Fig. 2a–c). Higher average GFP intensities with M3814 treatment indicate higher efficiencies of homozygous tagging. Strikingly, coIN plus M3814 showed synergistic effects with an increase in both the percentage of GFP-positive cells (from ~ 30 to ~ 70%) and the average GFP intensities (increase by ~ 40%) compared to control condition (Fig. 2b, c). M3814 increased *At*AFB2(F74A)-mCherry levels (Additional file 1: Fig. S3c), but showed considerable cytotoxicity, with a 60% reduction in puromycin selected cell numbers (from ~ 3.8 × 10^6^ to ~ 1.5 × 10^6^ cells/ml) (Fig. 2c).

I53 overexpression showed no cell toxicity and a combination of i53 plus 0.25 µM M3814 as HDR enhancers was further evaluated (Additional file 1: Fig. S3a, b). Testing of 10 degron-GFP tagging pairs demonstrated that i53 plus 0.25 µM M3814 outperformed 1 µM M3814 in cell counts and led to comparable increases in degron-GFP tagging efficiencies and *At*AFB2(F74A)-mCherry levels (Fig. 2d, e, and Additional file 1: Fig. S3c). Collectively, these results demonstrate that by combining coIN with HDR enhancers, high homozygous tagging efficiency and auxin receptor overexpression can be achieved in a single step in A431 cells.

Two small size selection markers, Blasticidin S deaminase (BSD) [30] and *Streptoalloteichus hindustanus* bleomycin (Sh_ble) genes [31], were tested as 2A-drug selection cassettes in HDR templates to further improve degron-tagging efficiencies. Results with Sh_ble are shown in Additional file 1: Fig. S4a, b and Note S3. BSD had higher sensitivity than Sh_ble, effectively enriching all targets tested, and was thus used in the templates for endogenous tagging (Fig. 2f). AID clones were then isolated as outlined in Fig. 1c, with genotyping PCR (gPCR) to evaluate the homozygous tagging efficiencies in isolated clones (Fig. 2g). Testing of 11 targets of widely different functions and expression levels [8] showed a degron-tagging efficiency of 100% (heterozygous plus homozygous) (Fig. 2h, i). Clones generated with HDR enhancers (either 1 µM M3814 or i53 plus 0.25 µM M3814) for the 11 targets showed significantly higher homozygous degron-tagging efficiencies (average of 81%, varying from 62 to 100%) compared to the 8 targets without HDR enhancers (average of 32%, varying from 0 to 83%) (Fig. 2h, i, and Additional file 1: Data S1). Thus, HDR enhancers increased homozygous tagging efficiency in coIN, in line with the FACS analysis. Of note, RABGGTA tagged with miniIAA7-GFP achieved high-efficiency homozygous tagging of 90% with HDR enhancers i53 plus 0.25 µM M3814 without BSD selection (Additional file 1: Fig. S4c, d). Together, these results show that the combination of coIN with the HDR enhancers achieves one-step generation of AID cells with high-efficiency homozygous tagging and that P2A-BSD effectively eliminates untagged A431 cells.

The inducible degradation and ensuing functional readouts were then characterized for the 12 targets generated with HiHo-AID2. A mouse monoclonal antibody against miniIAA7 (α-miniIAA7) was generated to facilitate the detection of degron-tagged proteins (see “Methods”). Based on Western blotting (WB), 11 of the target proteins were rapidly degraded in 1 h (Additional file 1: Fig. S5a), except for NUP93 that was effectively degraded after 6 h. NUP93 localizes in the nuclear pore complex and might have limited accessibility to *At*AFB2(F74A) [32].

Functional analysis of the targets revealed that upon pico_cvxIAA induction, most homozygous clones showed expected phenotypic changes that were not observed in heterozygous clones (Additional file 1: Fig. S5b-h) [33–35]. An interesting example is SAC1, the single known PI4P phosphatase in human cells that is required for cell viability [36, 37]. Rapid inducible degradation is thus optimal to study its functions but has not been reported before. WB analysis showed that SAC1 was largely depleted after 1 h induction with pico_cvxIAA (Fig. 2j), accompanied by a fourfold increase in PI4P antibody staining intensity at 3 h induction (Fig. 2k) and a clearly altered cell morphology 24 h after induction (Fig. 2l).

A few homozygous clones identified by gPCR with primers on the homology arms exhibited reduced protein levels before induction and showed no clear functional readouts (Additional file 1: Fig. S5a and d, ACSL4_clone 1), potentially due to large deletions or other rearrangements in the target loci undetectable with gPCR [19, 38]. It has been reported that about 10% of clones harbored long deletions when using M3814 as an HDR enhancer [19]. Indeed, long-range gPCR with arm-spanning primers detected 2 out of 16 selected homozygous clones of having additional deletions (Additional file 1: Data S1b). Thus, use of multiple homozygous clones identified by gPCR is therefore recommended for further long-range PCR, WB, and functional analyses to avoid clones with on-target mutations. Together, these results demonstrate rapid and effective removal of the degron-tagged proteins for all targets generated with HiHo-AID2 and excellent performance of *At*AFB2(F74A)/miniIAA7-3xFlag/pico_cvxIAA system for functional depletion of target proteins.

### Application of HiHo-AID2 in other commonly used cell lines

**Fig. 3:**
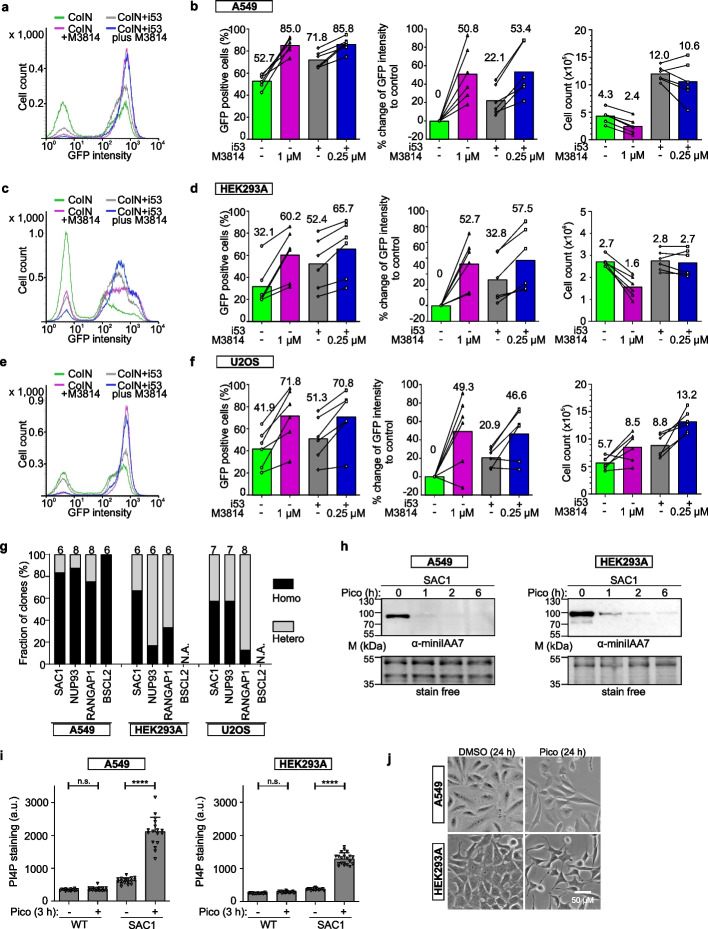
Evaluation and applications of HiHo-AID2 in other human cancer cell lines. **a**–**f** Comparison of degron-GFP tagging efficiencies in coIN with different HDR enhancers in A549 (**a**, **b**), HEK293A (**c**, **d**), and U2OS (**e**, **f**) cells. Representative FACS profile (**a**, **c**, **e**) and graphs depicting the percentage of GFP-positive cells, the percentual change of single-cell GFP intensity compared to control (coIN), and the cell count (× 10^5^ cells/ml) (**b**, **d**, **f**) are shown. Numbers above columns indicate mean values; lines link the same endogenous tagging pairs. *N* = 6 pairs of tagging plasmids. Statistical comparisons are shown in Additional file 4: Table S6. **g** Graphs showing the genotyping PCR results for AID clones generated with HiHo-AID2. Clones were generated and identified as indicated in Fig. 2f, g. Total number of clones analyzed is indicated above each column. N.A.: not available due to cell death after selection with blasticidin (S2). **h** WB analysis of inducible SAC1 degradation in A549 and HEK293A cells. **i** Graphs showing the PI4P staining intensity upon SAC1 degradation in A549 and HEK293A wild-type and SAC1 AID cells. *N* = 17 (A549) and 20 (HEK293A) fields. One-way ANOVA, n.s.: non-significant, **** *p* < 0.001. All statistical comparisons are shown in Additional file 4: Table S6. **j** Widefield imaging of cell morphological changes upon SAC1 degradation. Scale bar: 50 μM. Representative of 1 (A549) and 2 (HEK293A) clones (**h**–**j**). WT: wild-type; pico: 0.5 μM pico_cvxIAA treatment; a.u.: arbitrary unit

We next tested HiHo-AID2 in other widely used human cell lines, including lung alveolar cancer A549, embryonic kidney HEK293A, osteosarcoma U2OS, and prostate cancer-derived PC3 cells. CoIN of 6 endogenous degron-GFP tagging pairs (3 templates with 2 different sgRNAs each) was first performed with or without HDR enhancers. PC3 cells died out after stable puromycin selection, likely due to deficiency of the HDR repair pathway [39]. In the other 3 cell lines, effective degron-GFP tagging was achieved using coIN and further improved with HDR enhancers as in A431 cells (Fig. 3a–f). Substantially lower degron-GFP tagging efficiency was obtained through conventional tagging without coIN and HDR enhancer (Additional file 1: Fig. S6a). In A549, HEK293A and U2OS cells, i53 plus 0.25 µM M3814 again outperformed 1 µM M3814, yielding similar enhancement of HDR efficiency with lower cytotoxicity (Fig. 3a–f). With HDR enhancers, the expression of *At*AFB2(F74A)-mCherry was again slightly increased in all 3 cell lines (Additional file 1: Fig. S6b). These results indicate that HiHo-AID2 achieves efficient homozygous degron tagging in several human cancer cell lines proficient in HDR.

Single-cell clones were then generated as for A431 cells (Fig. 1c and Fig. 2f, g). Of the 3 cell lines, A549 showed the highest homozygous degron-tagging efficiency (average > 85% in A549 cells, > 40% in U2OS cells, and > 35% in HEK293A cells, for 3–4 targets) (Fig. 3g, and Additional file 1: Data S1c, e, g). For A549 and U2OS cells, clones were isolated directly from 10-cm plates. For HEK293A cells, limited dilution cloning into 96-well plates was used as the cells did not form clones with a clear boundary. Of note, BSCL2 degron clones could not be isolated in HEK293A and U2OS cells, as the clones died after blasticidin (S2) selection, indicating that the cells might express BSCL2 at lower levels or be more sensitive to blasticidin (Fig. 3g). A more sensitive selection marker as S2 might solve the issue. Alternatively, single clones could be isolated without S2 selection as for RABGGTA in A431 cells (Additional file 1: Fig. S4c, d). Similar to A431 cells, long-range PCR detected additional deletions on the target sites in 1 out of 8 (A549), 1 out of 6 (HEK293A), and 0 out of 5 (U2OS) clones (Additional file 1: Data S1d, f, h).

Regarding functional effects, WB and microscopy analyses of SAC1 degron A549 and HEK293A clones showed rapid protein degradation, an expected increase of cellular PI4P and morphological changes analogous to those observed in A431 cells (Fig. 3h–j). Similar changes as in A431 cells were also found for the other target proteins tested in A549 and HEK293A cells, including WB and phenotypic readouts (Additional file 1: Fig. S7a-d and f). In general, U2OS cells exhibited a somewhat slower degradation of all 3 target proteins post-induction, despite proper expression of *At*AFB2(F74A)-mCherry (Additional file 1: Fig. S7a and e). The slower degradation rate might be due to lower activity of other SCF E3 ligase components in these cells.

### Establishment and application of HiHo-AID2 in hESCs

**Fig. 4:**
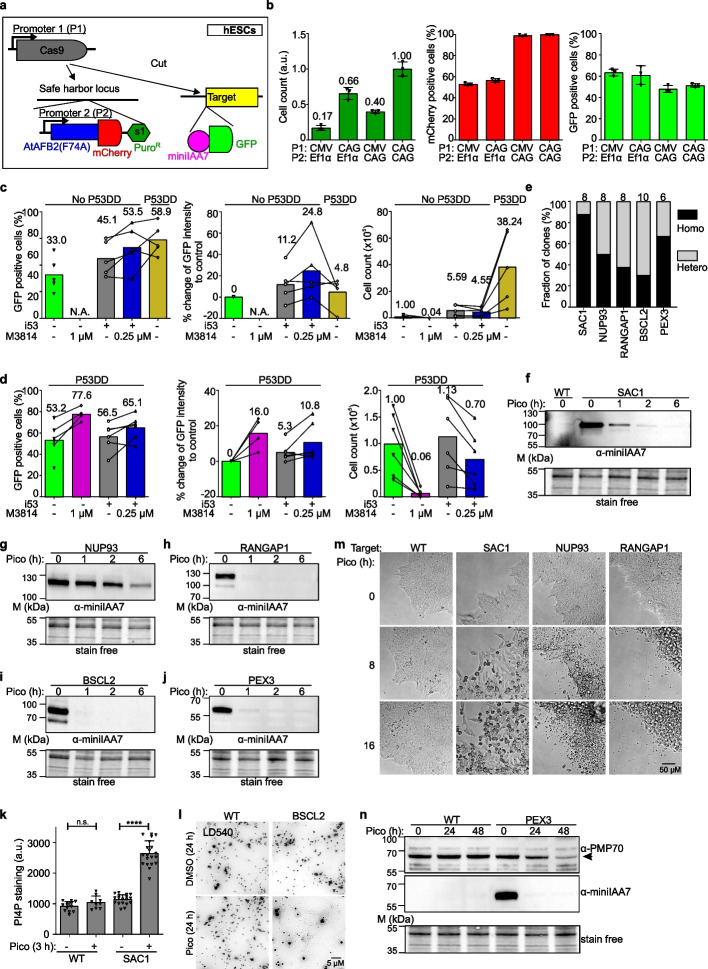
Optimization and application of HiHo-AID2 in hESCs. **a** Scheme illustrating the two promoters P1 and P2 in plasmids for one-step generation of AID cells. **b** Graphs showing the cell count, fraction of mCherry-positive and GFP-positive cells using different P1 and P2 promoters in hESCs. *N* = 3 technical repeats. **c**, **d** Graphs showing the percentage of GFP-positive cells, the percentual change of single-cell GFP intensity compared to control (coIN) and the cell count (× 10^5^ cells/ml) with different HDR enhancers with or without P53DD. P53DD: a dominant-negative P53 mutant; numbers above columns indicate mean values; lines link the same endogenous tagging pairs. N.A.: not available due to insufficient number of cells for FACS analysis. *N* = 5 (**c**) and 6 (**d**) different protein targets. All statistical comparisons are shown in Additional file 4: Table S6. **e** Graph depicting the genotyping PCR results for AID clones generated with HiHo-AID2 in hESCs using i53 plus 0.25 μM M3814 as HDR enhancers in the absence of P53DD. Clones were generated as indicated in Fig. 2f, g. Number above each column indicates total amount of clones analyzed. **f**–**j** WB analysis of inducible degradation of 5 target proteins in degron hESCs. **k** Graph showing the PI4P staining intensity of wild-type and SAC1 degron hESCs. *N* = 13, 9, 18, 20 fields respectively. One-way ANOVA, n.s.: non-significant, **** *p* < 0.001. All statistical comparisons are shown in Additional file 4: Table S6. **l** Lipid droplet (LD) staining with LD540 in wild-type and BSCL2 degron hESCs. Oleic acid (0.2 mM) was added during the final 4 h to induce LD formation. Scale bar: 5 μM. **m** Live-cell imaging analysis of morphological changes and cell death in wild-type and 3 different degron hESCs. Representative images in different time points from the same areas are shown. Scale bar: 50 μM; *N* = 4 fields. **n** WB analysis with anti-PMP70 and anti-miniIAA7 antibodies in wild-type and PEX3 degron hESCs. Arrow indicates the specific PMP70 protein bands. Representative of 2 clones for each target protein (**f**–**n**). WT: wild-type; pico: 0.5 μM pico_cvxIAA treatment; a.u. arbitrary unit

Homozygous tagging is of low efficiency in human pluripotent stem cells, e.g., hESCs [15, 16]. Accordingly, our early attempts to tag endogenous POGZ locus in hESCs using electroporation resulted in only 0.1% homozygously tagged cells (Additional file 1: Fig. S8a-c). Moreover, the expression of *At*AFB2(F74A) under the EF1a promoter was unstable in hESCs, leading to inefficient inducible degradation (Additional file 1: Fig. S8d, e). Further optimization revealed that the use of CAG promoter improved both stable expression of *At*AFB2(F74A)-mCherry (from about 60% mCherry-positive cells with EF1a promoter to 99% with CAG promoter) and *AAVS1* integration efficiency (by about 2.5-fold compared to CMV promoter to express Cas9) (Fig. 4a, b). These results emphasize the importance of the choice of promoters for successful establishment of auxin receptor expression and degron tagging in human stem cells [40].

We next tested degron-GFP tagging pairs for 5 different targets using coIN and HDR enhancers as well as P53DD, a dominant-negative P53 mutant. P53DD substantially improved the viability of engineered human stem cells that are sensitive to Cas9 induced double-strand breaks (DSBs) in a P53-dependent pathway [41]. We found that in coIN, transient expression of P53DD dramatically increased puromycin-resistant cell counts by roughly 40-fold, and the percentage of degron-tagged cells by almost twofold without a clear impact on single-cell GFP intensity (Fig. 4c). Interestingly, i53 increased the cell count by ~ fivefold in the absence of P53DD, but not in its presence (Fig. 4c, d). The results imply that i53 might partly inhibit a P53-dependent pathway to improve cell viability.

M3814 at 1 µM showed severe cytotoxicity that reduced the cell count by about 20-fold and was not rescued by P53DD (Fig. 4c, d). M3814 at 0.25 µM still showed severe cytotoxicity (Additional file 1: Fig. S8f), which could be relieved by addition of i53 (Fig. 4c, d). Moreover, i53 plus 0.25 µM M3814 increased the efficiency of degron tagging, single-cell GFP intensity, and cell count (Fig. 4c, d). Finally, HiHo-AID2 with i53 plus 0.25 µM M3814 as HDR enhancers allowed the isolation of hESC clones in 1.5 weeks and showed an average homozygous degron-tagging efficiency of > 50% (ranging from 30 to 87%) for 5 targets (Fig. 4e, and Additional file 1: Data S1i).

Long-range PCR with arm-spanning primers showed that 0 out of 4 hESC-AID clones had additional deletions on the target site (Additional file 1: Data S1j). Furthermore, BSCL2, PEX3, and SAC1 hESC-AID clones and their parental hESCs were subjected to whole genome sequencing (WGS) for further characterization. This revealed that HiHo-AID2 resulted in the correct knock-in of the respective targets and successful integration of *At*AFB2(F74A) into *AAVS1* safe harbor locus (Additional file 1: Fig. S9a-d, and Additional file 2: Table S1). Compared to the respective parental hESCs, no off-target editing was found in the hESC-AID clones and no copy number variation between parental hESCs and hESC-AID clones was observed (Additional file 2: Table S1). Moreover, the hESC-AID clones did not exhibit P53 mutations (Additional file 1: Fig. S9e). These results suggest that HiHo-AID2 enables efficient homozygous tagging in hESCs without increasing off-target effects.

### Protein degradation in hESCs

WB analysis showed that 4 of the targets were efficiently degraded in 1 h and NUP93 was again degraded more slowly (in about 6 h) upon pico_cvxIAA treatment (Fig. 4f–j). Functional analyses revealed expected phenotypes for all the targets: rapid increase of PI4P staining in SAC1 degron cells (3 h induction), lipid droplet biogenesis defects in BSCL2 degron cells (24 h induction), and extensive degradation of peroxisomal membrane protein PMP70 in PEX3 degron cells (24–48 h induction) (Fig. 4k, l, and n). Furthermore, live-cell imaging showed specific and distinct morphological changes preceding cell death in SAC1, NUP93, and RANGAP1 degron cells within 16 h of induction (Fig. 4m). WB with Oct3/4 antibody indicated that the isolated degron cell lines maintained their pluripotency (Additional file 1: Fig. S8g). Together, these results demonstrate that the AID2 system is robust and effective for chemogenetic control of endogenous proteins in hESCs.

### Chemogenetic control of endogenous protein degradation in hESC-derived cells

**Fig. 5:**
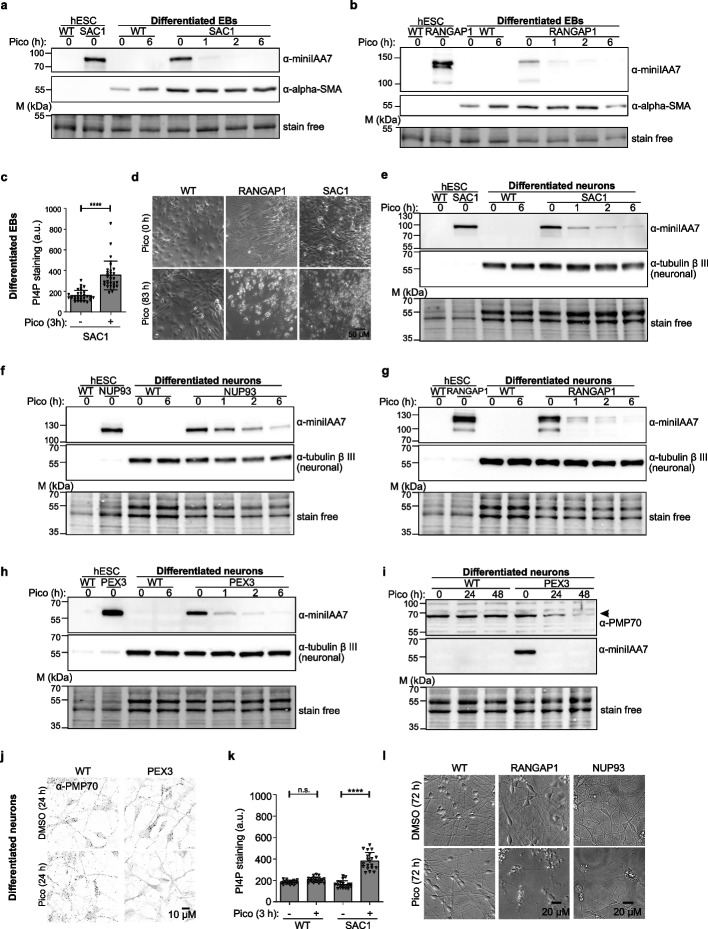
Chemogenetic control of endogenous protein degradation in hESC-derived cells. **a**, **b** WB analysis of EBs differentiated from SAC1 (**a**) and RANGAP1 (**b**) degron hESCs. Blotted for miniIAA7 and α-SMA. WT: wild-type; pico: 0.5 μM pico_cvxIAA treatment. **c** Graph showing PI4P staining intensity in EBs differentiated from SAC1 degron hESCs. Student’s *t* test, ****p < 0.001. All statistical comparisons are shown in Additional file 4: Table S6. **d** Representative images from live-imaging of cell death in EB differentiated from wild-type, RANGAP1, and SAC1 degron hESCs after 83 h of pico_cvxIAA induction. Scale bar: 50 μM. WT: wild-type; pico: 0.5 μM pico_cvxIAA treatment. **e**–**h** WB analysis in neurons differentiated from degron hESCs targeting SAC1 (**e**), NUP93 (**f**), RANGAP1 (**g**), and PEX3 (**h**). WT: wild-type; pico: 0.5 μM pico_cvxIAA treatment. **i**, **j** WB analysis (**i**) and immunofluorescence staining (**j**) of peroxisomal membrane protein PMP70 in neurons differentiated from wild-type and PEX3 degron hESCs. Scale bar: 10 μM. **k** Graph showing the PI4P staining intensity in neurons differentiated from wild-type and SAC1 degron hESCs. a.u. arbitrary unit. *N* = 20 fields. **l** Representative images of live-cell imaging of cell death in neurons differentiated from wild-type, RANGAP1, and NUP93 degron hESCs. Scale bar: 20 μM. Representative of 1 (RANGAP1 and NUP93) and 2 (SAC1 and PEX3) cell clones

Theoretically, hESCs have the capability to give rise to all somatic cell types of an embryo. To investigate whether HiHo-AID2 edited hESCs maintain pluripotency and inducible protein degradation after differentiation, we selected the embryoid body (EB) model. Aggregation of hESCs into 3-dimensional EB structures has a general inductive influence and is frequently used as a first step in in vitro differentiation of many cell lineages. We found that the hESC-AID clones could differentiate into the expected three germ layer derivatives (Additional file 1: Fig. S8h). Furthermore, rapid and efficient degradation of target proteins (SAC1 and RANGAP1) was observed in the EBs derived from hESC-degron cells (Fig. 5a, b). Of note, EB formation is heterogenous and may affect target expression levels when compared to hESCs (as for RANGAP1 in Fig. 5b). Yet, the expected phenotypic effects were evident upon inducible protein degradation: the increase in PI4P signal upon SAC1 degradation in EBs was observed in 3 h (Fig. 5c) and cell death in both SAC1 and RANGAP1 EBs in 83 h after pico_cvxIAA addition (Fig. 5d).

As EB formation produces spontaneous differentiation, we also specifically differentiated hESC-AID cells into neurons with an established protocol [42]. Morphological and WB analysis with the neuronal marker anti-β-Tubulin III confirmed the successful neuronal differentiation of the hESC-derived AID cell lines (Fig. 5e–h). Upon pico_cvxIAA induction, rapid and efficient depletion of the target proteins was achieved in the hESC-neurons, as shown by WB (in 1 h for SAC1, PEX3 and RANGAP1; in 6 h for NUP93). This was followed by expected functional readouts, i.e., degradation of PMP70 in PEX3 degron cells (Fig. 5i, j), an increase of PI4P staining in SAC1 degron cells (Fig. 5k) and cell death in RANGAP1 and NUP93 degron cells (Fig. 5l).

Altogether, these data provide the first demonstration of AID for chemogenetic control of endogenous proteins in different cell lineages derived from hESC-AID lines, revealing the potential of AID for dissecting diverse biological processes in differentiated cell types.

## Discussion

Loss-of-function analysis is one of the most important strategies for understanding protein functions in mammalian cells [43]. AID provides a powerful means to achieve this in a rapid, inducible manner but requires the introduction of two genetic modifications. So far, efforts for one-step generation of AID cells have employed a rescue strategy through CRISPR/Cas9-mediated knock-out in conjunction with the expression of a degron-tagged rescue construct plus an auxin receptor [44–46]. Extensive identification of single clones was needed, and the targets were biased towards essential proteins [44–46]. Moreover, this strategy may suffer from downsides of CRISPR knock-out, including compensations that can rescue target activities [47, 48]. Critically, single clones show considerable variations of target protein expression as the rescue constructs are randomly inserted into the genome at various copy numbers [44–46]. The rescued cells further suffer from lack of endogenous transcriptional and translational control, such as response to environmental stimuli or cell differentiation cues.

Here, we harnessed recent developments of the AID technology and employed the AID2 system that combines non-leakiness with low inducer concentration and negligible off-target effects [8, 9, 11, 13]. We then established HiHo-AID2, a streamlined procedure for one-step generation of AID cell clones through endogenous degron tagging with an unprecedented speed of about 10 days and tagging efficiencies of 100% in the isolated clones (homozygous plus heterozygous), for multiple target proteins in 5 cell lines**.** This was achieved by integrating an optimized AID2 system with the use of coIN and HDR enhancers. The combination of i53 plus 0.25 µM M3814 as HDR enhancers was found to be optimal due to low cytotoxicity, especially in hESCs. We typically used blasticidin to enrich the knock-in cells with a P2A-BSD cassette, but this was not always necessary (as in the case of RABGGTA; Additional file 1: Fig. S4c, d), nor useful in case the target protein was expressed at too low levels to be selected with BSD.

Extensive isolation of single clones was not required in HiHo-AID2, since (1) the tagged proteins were expressed under the control of endogenous promoters and (2) high-efficiency homozygous tagging was achieved with near 100% expression of the auxin receptor. The cell clones isolated largely represent *bona fide* single clones, as gPCR of homozygous clones showed no extra band at the wild-type position (Additional file 1: Data S1) and functional characterization of several clones at single-cell level provided no indication of contamination from wild-type or heterozygously tagged cells (Additional file 1: Fig S5, S7). Of note, further characterization with long-range PCR indicated that about 10% of the identified clones harbored additional large on-target deletions, as previously reported [19]. Therefore, careful genomic characterization of several clones in combination with available functional readouts is recommended.

The target cell line has a major impact on the established procedure. Cells deficient in HDR, such as PC3, are not suitable for it [39]. Of the cell lines tested, A431 and A549 cells were the most proficient, while HEK293A and U2OS displayed somewhat lower homozygous tagging efficiencies. For cell lines that are deficient in HDR, recent developments in genetic engineering tools, such as PASTE [49] that combines prime editing [50] with site-specific integrase [51], might enable homozygous tagging of endogenous proteins.

Chemical induction of proteolysis is particularly attractive for stem cell biology as it is non-invasive, rapid, efficient, and flexible [52]. Moreover, stem cells can be differentiated into cell types that are not proficient in HDR [53]. So far, there is only a single report, with no functional verification, on the application of AID for an endogenous protein in human stem cells [17], and no reports on AID of endogenous proteins in differentiated human cells.

In the present study, we further optimized HiHo-AID2 for hESCs and their differentiated progeny by identifying a promoter that enables uniform auxin receptor expression in these cells and by mitigating cytotoxicity using i53. With these improvements, we successfully isolated homozygously tagged cell clones for multiple targets with different expression levels and functions as efficiently as for cancer cells, in about 10 days. WGS analysis on 3 of the resulting clones showed no off-target effects and no mutations at P53 loci (Additional file 1: Fig. S9e). Of note, hESC clones with mosaic expression were observed in some other attempts, possibly due to inefficient single-cell dissociation and/or the rapid proliferation of hESCs. A further step of single-cell cloning after generating AID cell pools may be applied in such cases.

Finally, by employing HiHo-AID2 we achieved chemogenetic degradation of several endogenous targets in hESCs with high efficiency. We provided the first evidence that AID removes endogenous proteins also in cells differentiated from them, using EBs and neurons as test cases. Neurons stand out from several other cell types in their high degree of compartmentalization and prominent local regulation of proteasomal activity. We were therefore initially concerned about issues regarding the accessibility of *At*AFB2(F74A) to its substrates as well as the poly-ubiquitination and proteasomal degradation of substrates after the inducible interaction of *At*AFB2(F74A) with the miniIAA7 degron. Excitingly, our results show that AID can induce rapid and efficient protein degradation in hESC-derived neurons, despite a moderate reduction in degradation efficiency compared to hESCs. This holds major promise for stem cell biology: genetic engineering in human stem cells followed by targeted cell differentiation will now enable rapid inducible degradation of endogenous proteins in various differentiated cell types, opening new possibilities for, e.g., disease modeling and cell therapy.

## Conclusions

The HiHo-AID2 method established here provides a robust genome-editing pipeline for high-efficiency homozygous knock-in and auxin receptor expression in several commonly used human cell lines, including hESCs, in a single step in ~ 10 days. The established AID cells exhibited rapid and efficient degradation of a broad spectrum of endogenous target proteins, accompanied by expected functional outcomes. The edited hESCs could be further differentiated into EBs and neurons, from which endogenous proteins were inducibly and acutely removed and cellular functions altered. In summary, HiHo-AID2 boosts homozygous knock-in and assists in the implementation of AID, including cells challenging to engineer such as hESCs and their differentiated counterparts, and provides the first demonstration that AID can efficiently remove endogenous proteins from differentiated human cells.

## Methods

### Culture and transfection of human cancer cell lines

A431 cells (ATCC CRL-1555), U2OS cells (kindly provided by Marikki Laiho at John Hopkins University, USA), and HEK293A cells (Invitrogen R70507) were cultured in DMEM (high-glucose, Lonza/Gibco), and A549 cells (ATCC CCL-185) in F-12 Nutrient Mixture (Gibco), supplemented with 10% FBS, penicillin/streptomycin (100 U ml^−1^ each), L‐glutamine (2 mM) at 37 °C in 5% CO_2_. Cells were tested negative for Mycoplasma using PCR detection. Cells were seeded for 16 h in 12-well and transfected at 80–95% confluence using Lipofectamine LTX with PLUS Reagent (Invitrogen 15338–100), typically with 1.0 µg plasmid(s) per 1.0 µl of PLUS reagent (1.5 µl for HEK293A), 2.0 µl of Lipofectamine LTX (3.0 µl for HEK293A), and 4.0 × 10^5^ (A431 and HEK293A) or 3.0 × 10^5^ (U2OS and A549) cells per well.

### Chemicals and antibodies

Nedisertib (M3814, Selleckchem S8586) prepared as 10 mM stock in DMSO; cvxIAA (TCI M3141) 25 mM stock in DMSO, 5-Adamantyl-IAA (pico_cvxIAA, TCI A3390) 2.5 mM diluted from 100 mM stock in DMSO, 5-PH IAA (MCE HY-134653) 20 mM in DMSO, Indole-3-acetic acid sodium (IAA, Sigma I5148) 0.25 M stock in H_2_O, puromycin (Sigma, P8833) 10 mg/ml in H_2_O, Blasticidin S HCl (Gibco A1113903) 10 mg/mL solution, Zeocin (Gibco R25001) 100 mg/ml solution, LD540 (synthesized by Princeton BioMolecular Research) 1 mg/ml in Ethanol, Erastin (Selleckchem S7242) 10 mM in DMSO, Oleic Acid (OA, Sigma O-1383) 1 mM as OA:BSA complex (8:1 molar ratio) in serum-free DMEM, goat anti-FABP4 (WB 1:500, Santa Cruz sc-18661), mouse monoclonal anti-tubulin beta III (neuronal) (WB 1:2000 Sigma, T8578-100UL), mouse monoclonal anti-PMP70 (WB 1:1000, Sigma-Aldrich SAB4200181), mouse monoclonal anti-PI4P IgM (IF 1:200, Echelon Z-P004), mouse monoclonal anti-Oct3/4 (WB 1:500, Santa Cruz sc-5279), goat polyclonal anti-SOX17 (IF 1:500, R&D Systems AF1924), mouse monoclonal anti-α-Smooth Muscle Actin (SMA) (IF/WB 1:500, Sigma-Aldrich A2547), goat anti-mouse IgG (H/L) HRP (1:1000, Bio-Rad 1,706,516), donkey anti-goat (H/L) IgG HRP (1:10,000, Jackson immuno research 705–035-147), goat anti-mouse IgM (H) Alexa Fluor 488 (1:200, Thermo Fisher A21042). Mouse anti-miniIAA7 (WB final 0.5 µg/ml, Ximbio 158,027) was generated by Genscript. Briefly, the 20 aa. core peptide of miniIAA7 tag with an extra C conjugated to KHL (QVVGWPPVRNYRKNMMTQQKC-KHL) was used as the immunogen and BALB/c mouse was used as the host strain. Antisera from 5 mice were tested in WB with endogenous miniIAA7 tagged A431 cell lysates. The best one was chosen for cell fusion to generate hybridoma cell clones. The clones were screened again with WB and 5 of the best clones were selected for subcloning to get the final clone 12A8-1. 12A8-1 was used for antibody production to generate purified mouse IgG and designated as mouse anti-miniIAA7 (12A8-1).

### Construction of plasmids

Vector sequences and sgRNA sequences are provided in Additional file 3: Table S2 and S3.

To express different auxin receptors through *AAVS1* integration, pSH-EFIRES-P-*At*AFB2-mCherry (Addgene 129716) was used as a backbone. *Os*TIR1 was synthesized by Genscript as codon-optimized cDNA and substituted *At*AFB2 on the backbone through restriction ligation. Overlap PCR was used to introduce F74A and F74G point mutations in the auxin receptors. CAG promoter was cloned from plasmid AAS1815 (Addgene 107942) [54] to substitute EF1a promoter on the backbone where indicated.

To express Cas9 and different sgRNAs, sgRNAs were synthesized as two unphosphorylated primers, annealed and inserted into BbsI-cut pCas9-sgRNA (as Addgene 129726) or pCas9/VRQR-sgRNA (Addgene 129725) vectors. CAG promoter from plasmid AAS1815 (Addgene 107942) was used to substitute the CMV promoter on the vectors to express Cas9 where indicated. SgRNA targeting sites were searched manually for –NGG PAM sequence within 18 bp after insertion sites or CCN- within 18 bp before insertion sites. The sgRNA target sites were disrupted in the templates by the insertions.

To construct HDR templates of endogenous targets, homology arms on donor vectors were amplified from A431 genomic DNA through PCR using Q5 High-Fidelity DNA Polymerase with High GC Enhancer (NEB, M0491). Tags were synthesized as codon-optimized cDNA and cloned into the donor through restriction ligation or overlap PCR. The PCR fragments were cloned into pGL3-basic backbone using NEBuilder HiFi DNA Assembly Master Mix (NEB, E2621) or through restriction ligation. Tags on the HDR templates were changed through restriction ligation or Gibson assembly with NEBuilder HiFi DNA Assembly Master Mix.

P53DD and i53 were synthesized as codon-optimized cDNAs by Genscript and cloned into pGL3-basic vector with EF1a promoter and HSV TK poly(A).

### A431 cell lines for testing different auxin receptors

pSH-IRES-B-BSCL2-miniIAA7-mEGFP (Addgene 129719) was used to generate single clones expressing BSCL2-miniIAA7-mEGFP through HDR-mediated *AAVS1* integration. A431 single clones with homozygously tagged DHC1-miniIAA7-mEGFP, BSCL2-miniIAA7-mEGFP, and heterozygously tagged EGFR-miniIAA7-mEGFP have been described [8]. To express different auxin receptors, *AAVS1* integration vector expressing auxin receptors (0.6 µg) and pCas9-sgAAVS1-1 (0.4 µg, Addgene 129726) were co-transfected into cells with Lipofectamine LTX with PLUS Reagent for 24 h, followed by selection with 1 µg/ml puromycin for 6 days. Cell pools were used for FACS and live-cell imaging.

### HiHo-AID2 for generation of human AID clones in cancer cell lines

An overview of the procedure is provided at Fig. 1c. A431, U2OS, HEK293A, and A549 cells were seeded on 12-well plates at day 0 and transfected with a mixture of 4 plasmids (*AAVS1*: target at 1:3 ratio as shown in Additional file 1: Fig. S2e) or 5 plasmids (0.8 µg of 4 plasmids plus 0.2 µg i53 plasmid) at day 1 using Lipofectamine LTX with PLUS Reagent. After 4–6 h, one third of transfected cells was passaged to a 10-cm dish containing the indicated concentration of M3814 (0.25 or 1 µM). At day 2, medium was replaced with fresh medium containing 1 µg/ml of puromycin and the same concentration of M3814 as in day 1. At day 4, fresh medium with puromycin but without M3814 was used. At days 6 and 8, fresh medium with 10 µg/ml Blasticidin was used to select clones with endogenous tagging. Blasticidin was omitted in the case of RABGGTA or changed to 100 µg/ml of Zeocin in cases where Sh_ble was used as S2.

At days 9–10, single clones formed on the 10-cm plates. Picking of single clones is analogous to iPS clone picking with videos available online, it can generally be learned on the first attempt and is easy to master. Briefly, a S9 E StereoZoom microscope (Leica, 10450814) with 10 × Eyepieces on a TL3000 ergo light base (Leica, 10450690) was set up to check and isolate single clones. Before picking of single clones, medium on the 10-cm plates were changed to PBS or antibiotic-free medium to help the survival of clones after picking. A 24-well plate with regular medium was prepared to grow the clones. Clones formed on the 10-cm plate can be visualized on the TL3000 ergo light source with proper contrast. Clones on the plate were moved to the centre, checked under the objective, and picked with a regular 10- or 20-µl pipette. The pipette with a tip was gently pressed beforehand and the single clones were detached by the pipette tip with gentle mechanical scraping. When cells were detached, the pressed pipette was released slowly to suck in the detached cells and the cells were transferred to 24-well plate with regular growth medium. Two to three days later, clones on the 24-well plates can be passaged with trypsin for further expansion and characterization. Of note, single clones on the 10-cm plate are growing faster than on a 96-well plate, as on the 10-cm plate gas exchange and cell–cell communication are maintained better, and medium can be simply changed to improve cell growth. Moreover, clone densities on the 10-cm plate are flexible and densities of 1–1000 clones per plate can be easily isolated.

### FACS analysis

Cells were seeded at 1:5 (for A431 and HEK293A) or 1:3 (for A549 and U2OS) into a six-well plate. On day 1, medium was changed to 2 ml fresh medium without (for 0 h and 1 h induction) or with (for 16 h induction) inducers. On day 2, the 1-h samples were supplemented with 0.5 ml medium containing 5 × concentration of the indicated inducer and incubated for 1 h at 37 °C. After treatment, cells were detached with 0.5 ml trypsin at 37 °C for 5–8 min (U2OS, HEK293A and A549) or 8–12 min (A431), put on ice and transferred to 1.5-ml Eppendorf tubes containing 0.5 ml serum-free CO_2_ independent medium (Gibco 18045088). The cell suspensions were centrifuged at 4 °C, resuspended in 0.3-ml ice-cold serum-free CO_2_ independent medium and stored on ice before FACS analysis. FACS analysis was performed on a BD Influx cell sorter (BD Biosciences) with a 100-µm nozzle at 4–8 °C using BD FACS Software. Cells were gated with SSC, FSC, and trigger pulse width for singlets, and 50,000–100,000 cells were analysed from each sample. GFP was excited with a 488-nm laser and detected with a 530/40 detector; mCherry was excited with a 561-nm laser and detected with a 615/20 detector. Data were analysed with BD FACS Software. Background subtracted mean fluorescence intensity was used for analysis.

### Cell counting

Cells were counted with Bio-Rad TC10 automated cell counter (Bio-Rad 145–0001) using 10–20 µl of cell suspension in TC10 counting slides (Bio-Rad 145–0015). Histograms of cell diameter distribution were checked after each count to avoid counts with abnormal histograms.

### Reversibility assays

For inducer washout experiments, cells were seeded at day 1 on μ-slide 8-well ibiTreat dishes. On the second day, cells were treated with the indicated inducers overnight. On the third day, cells were washed 4 times with FluroBrite DMEM containing 10% FBS without inducer before live-cell imaging. Cells were imaged immediately after washing with a Nikon Eclipse Ti-E widefield microscope equipped with × 20 air objective NA 0.8, Nikon Perfect Focus System 3, Hamamatsu Flash 4.0 V2 scientific CMOS and Okolab stage top incubator system. Multipoint and time lapse imaging was started immediately, and recording was every 30 min for 18 h. Background subtracted fluorescence intensities were used for analysis.

### RNAseq and analysis

For the RNAseq data presented in Additional file 1: Fig. S1m, RNA samples were extracted using NucleoSpin RNA Mini kit (MACHEREY–NAGEL 740955). For RNA sequencing, library was prepared using NEB Ultra II directional RNA library prep kit (NEB E7760). Samples were sequenced using an Illumina HiSeq 4000 with 75 bp paired-end reads. Sequencing reads of all samples were first quality controlled using FastQC v0.11.8 (http://www.bioinformatics.babraham.ac.uk/projects/fastqc/), followed by a trimming process using trimmomatic v0.38 [55] to obtain high-quality reads. Qualified reads were then mapped to GRCh38 with STAR v2.7.0e [56], and subsequently went through an indexing process with samtools v1.10 [57]. Finally, the aligned files were used for generating gene counts by featureCounts programme under subread v2.0.0 [58]. All the mapping process was done on the Hawk high-performance computing system based at Cardiff University (https://portal.supercomputing.wales/index.php/about-hawk/).

For downstream analysis, the generated gene counts matrix was filtered, to assess genes expressed in at least 50% of the samples. DESeq2 was next applied for differential expression analysis between groups of comparisons [59]. A Benjamini–Hochberg-adjusted *p* value of < 0.05 was considered as statistically significant. To visualize the differential expression results, ggplot2 v3.3.6 package was used to generate volcano plots for the comparisons of interest (https://ggplot2.tidyverse.org). Downstream analysis and visualization were done on R v4.0.3 (https://www.R-project.orghttps://www.r-project.org/).

### Genotyping PCR

Primer sequences for PCR are provided in Additional file 3: Table S4 and S5. Genomic DNA from cultured cells was extracted using the NucleoSpin Tissue kit (Macherey–Nagel 740952). Finally, DNA was eluted in 60 µl elution buffer. Genotyping PCR was performed with Q5 PCR DNA polymerase (NEB M0491) plus GC enhancer using 2–4 µl of genomic DNA in 50 µl reaction. PCR products were analyzed on 2–2.5% agarose gels and imaged with a ChemiDoc MD Imaging System (Bio-Rad).

### Western blot

Cells were washed twice with ice-cold PBS and lysed in RIPA lysis buffer (1% NP-40, 0.1% SDS, 0.5% sodium deoxycholate, in 1 × TBS) with protease inhibitors (25 µg/ml chymostatin, 25 µg/ml leupeptin, 25 µg/ml antipain hydrochloride, 25 µg/ml pepstatin A). Protein concentration was measured using DC™ Protein Assay Kit I (Bio-Rad, 5000111) and 10–15 µg of protein was loaded on Mini-Protein TGX Stain-Free gels (Bio-Rad, 1610181, 1610183, 1610185, and 5678094) and run at 120 V in 1 × SDS-Page running buffer. After running, gels were activated using the ChemiDoc MD Imaging System (Bio-Rad) and transferred onto 0.45-µm Low Fluorescence PVDF membranes (Bio-Rad, 1704274). Membranes were blocked with 5% skim milk in 0.1% Tween-20 in TBS (TBS-T) for 45 min and subsequently incubated with primary antibodies diluted in blocking buffer overnight at 4 °C. Membranes were washed 3 × in TBS-T and incubated with secondary antibodies at room temperature for 45 min. Membranes were washed 3 × with TBS-T and incubated with Clarity Western ECL substrate (Bio-Rad, 1705061) and imaged using a ChemiDoc MD Imaging System (Bio-Rad).

### PI4P staining and analysis

Cellular PI4P staining was performed with mouse anti-PI4P IgM antibody (Echelon, Z-P004). Briefly, cells were fixed with 2% paraformaldehyde (Electron Microscopy Sciences, 15710) in either 1 × PBS or culture medium for 15 min at room temperature and permeabilized with 20 µM digitonin (Sigma-Aldrich, 11024–24-1) for 5 min. Cells were then blocked for 1 h with a blocking solution containing 1% fatty acid-free BSA (Sigma, a-3803) in 1 × PBS. Subsequently, cells were incubated with anti-PI4P antibody (1:200), washed 3 times with PBS, then incubated with Alexa FluorTM 488 labeled goat anti-mouse IgM secondary antibody (1:200, ThermoFisher A21042), and washed again at room temperature. Images were taken with Nikon Eclipse Ti-E microscope and analyzed with ImageJ software.

### Lipid droplet staining

A431 and A549 wild-type and BSCL2 degron cells were seeded onto Ibidi 8-well Labtek dishes (Ibidi, 155409) and treated for 24 h with DMSO or pico_cvxIAA. During the final 2 h, 0.2 mM oleic acid was added as OA:BSA complex. Lipid droplets were stained with the lipid droplet dye LD540 (1:2000) by adding the dye to medium for the final 20 min. Lipid droplets were imaged by Nikon Eclipse Ti-E widefield microscope using a Plan Apo VC × 100 oil DIC N2 objective.

### Generation of AID hESCs cells for POGZ

Human embryonic stem cells line (H9, WiCell, WIC-WA09-RB-001) was used for this study. hESCs were detached as single cells from the culture dishes with StemPro Accutase (Thermo Fisher A1110501) and washed with PBS. Cells were electroporated using the Neon transfection system (Invitrogen). A total of 2.5 × 10^6^ cells and plasmid mixture, containing Alt-R Cas9 nuclease, sgRNA targeting POGZ (TCTGATGGAGATTTGAGTGT TGG), electroporation enhancer, and the donor template plasmid (POGZ-miniIAA7-GFP), were electroporated in a 100-µL tip with 1100 V, 20 ms, and 2 × pulse settings. Electroporated hESCs were plated on Matrigel-coated 35-mm dishes in mTeSR medium containing 10 µM ROCK inhibitor Y-27632 2HCL (Selleckchem S1049) and 1 µM Alt-R HDR Enhancer. After 24 h, medium was changed to mTeSR medium without ROCK inhibitor or HDR enhancer, and the cells were further cultured until 72 h. The HDR efficiency was checked with FACS analysis. GFP + cells were single-cell sorted into 96-well plates, and clones were expanded and checked with gPCR for identifying homozygous or heterozygous tagging. One homozygously tagged clone was selected to introduce *At*AFB2 (F74A)-SNAPf-weakNLS with BSD selection marker into *AAVS1* locus by electroporation, followed by blasticidin selection for 2 weeks.

### HiHo-AID2 for generation of AID clones in hESCs

hESCs were cultured in mTeSR Plus culture medium (Stem cell technologies, 100–0276) on plates coated with Matrigel (Corning, 356231) diluted 1:200 in DMEM/F-12 (Gibco, 31331–028). Cells were detached by washing carefully 1–2 × with PBS (Corning, 21–040-cv), and subsequently treated with 0.5 µM EDTA (Invitrogen 15575020, diluted 1:1000 in PBS) for 4–5 min at RT. Alternatively, cells were passaged with StemPro EZPassage Disposable Stem Cell Passaging Tool (Invitrogen, 23181–010) and scraping in mTeSR Plus medium.

For transfection of hESCs, 5 plasmids (including i53 as for cancer cell lines mentioned above) were used. The medium was changed to fresh mTeSR Plus medium 3–5 h before passaging. hESCs were washed with PBS, incubated with StemPro Accutase (Thermo Fisher, A1110501) for 5–6 min at 37 °C, and centrifuged for 4 min at 1000 rpm. The cell pellet was resuspended in 2-ml mTeSR Plus medium with 10 µM Y27632 (Selleckchem, S1049). Cells were counted using a TC10 Automated cell counter (Bio-Rad) and histograms were checked to avoid counts with abnormal distributions. 3 × 10^5^–5 × 10^5^ cells were passaged to a 6-well in mTeSR medium with 10 µM Y27632. On day 1, medium was changed to 2 ml of fresh mTeSR Plus medium with 10 µM Y27632. hESCs were transfected using 5 µl Lipofectamine STEM transfection reagent (Thermo Fisher, STEM00015) with 2.5-µg plasmids. At 4–6 h post-transfection, medium was changed to fresh medium with 10 µM Y27632 and different concentrations of M3814 (0, 0.25, and 1 µM). On day 2, medium with 10 µM Y27632, 0.5 µg/ml puromycin, and indicated concentration of M3814 was added to the cells. Medium was changed to mTeSR Plus medium supplemented with M3814 and puromycin on day 3. On day 4, puromycin medium was added to the cells, followed by 2–3 days of 10 µg/ml Blasticidin selection until colonies were picked. Before picking colonies, medium was changed to fresh mTeSR Plus medium without antibiotics and 6–8 colonies per transfection were picked and transferred to a 4-well dish with 0.5 ml of mTeSR Plus medium. Medium of colonies was changed every 2 days for clonal growth.

### Lipid droplet staining in hESCs

hESC wild-type and BSCL2 degron cells were seeded in mTeSR with Y27632 onto Matrigel-coated Ibidi 8-well Labtek dishes (Ibidi, 155409) and treated for 24 h with DMSO or pico_cvxIAA. During the final 4 h, 0.4 mM oleic acid (prepared as a 1 mM OA-BSA complex at a 8:1 molar ratio to BSA in serum-free DMEM) was added to the cells. Lipid droplets (LDs) were stained with the lipid droplet dye LD540 by adding the dye to mTeSR medium for the final 20 min (1:2000, Princeton BioMolecular Research). Lipid droplets were imaged using a Nikon Eclipse Ti-E widefield microscope with a Plan Apo VC × 100 oil DIC N2 objective. Z-stacks were taken with a 0.3-µm interval. Images represent maximal projections, and brightness and contrast were adjusted in ImageJ.

### Whole genome sequencing of hESCs

Whole genome sequencing (WGS) was performed by CeGaT GmbH. Wild-type (× 2), BSCL2, PEX3, and SAC1 degron hESCs were subjected to WGS. All steps described in the following section were performed at CeGaT GmbH, Tübingen, Germany. DNA quantity of the samples was measured with Quant-iT dsDNA Broad-Range Assay Kit (Thermo Fisher Scientific) using the Gemini XPS microplate reader (VWR). One hundred nanograms DNA of each sample was used for library preparation with the TruSeq Nano DNA Library Prep Kit (Illumina) according to the manufacturer’s recommendations. Next-generation sequencing was performed on a NovaSeq 6000 platform (Illumina) with 2 × 150 bp read mode. The generated sequencing data were demultiplexed with Illumina bcl2fastq (2.20). Adapters were trimmed with Skewer (version 0.2.2) [60]. Quality trimming of the reads has not been performed. Sequencing data analysis was performed using the Illumina DRAGEN platform (software version 4.2.4). Reads were mapped to the reference genome hg19, and duplicates were marked. Calling of small variants, regions of homozygosity, and structural variants was performed with default parameters. Copy number variations (CNVs) were called in self-normalization mode. Variants between specified samples were compared using bcftools [57]. Potential off-targets for guide RNAs A-D were predicted using Cas-OFFinder [61] allowing for up to two mismatches. Variants close to the off-targets were extracted using an in-house tool.

### Differentiation of hESCs to neurons

Human neurons were derived by differentiating human hESCs using a small-molecule cocktail as described before, with minor adjustments [42]. N2B27 medium (N2B27 basal medium; 50% DMEM/F12 and 50% Neurobasal medium supplemented with 0.5 × N2 and 0.5 × B27, 1 mM GlutaMAX, and 1 × Penicillin–Streptomycin) was used throughout the differentiation protocol. Before induction, hESCs were passaged in mTeSR™ Plus and replated to form a uniform monolayer of cells. When the cells had reached around 95% confluence, medium was replaced with dual SMAD inhibition medium: N2B27 supplemented with 2 µM dorsomorphin (Selleckchem S7306) and 10 µM SB431542 (Sigma S4317). On day 10, the cells were replated in 1:2 ratio using 200 U/mL Collagenase IV (Gibco) onto Matrigel-coated dishes in N2B27 supplemented with 10 µM Y-27632 (Selleckchem S1049). From day 11 to day 20, N2B27 was supplemented with 100 ng/mL of FGF8 (PeproTech AF-100–25). On day 20, cells were detached using 0.5 mM EDTA and replated in N2B27 at 1:8 ratio. The next day following the split, N2B27 was supplemented with 20 µM DAPT (Selleckchem S2215) and the medium was replaced every 2 days.

### Preparing neuronal samples for WB, PMP70 staining, and live-cell imaging

The hESC-derived neurons were collected on day 25 of differentiation. For WB, neurons were grown in 35-mm dishes. Medium was removed, and neurons were washed once with 1 mL DMEM/F12 and once with 1 mL PBS. Neurons were detached with cell scraper, collected into a 1.5-mL Eppendorf tube, and centrifuged at 300 × *g* for 3 min. The supernatant was removed, and cells were lysed in RIPA buffer for WB analysis. For PMP70 staining, neurons were grown on Matrigel-coated coverslip. Medium was removed, and neurons were washed once with DMEM/F12 and once with sterile PBS. Neurons were fixed with 2% PFA for 15 min. For live-cell imaging, neurons were grown on ibidi plate (Cat.80826) and processed for imaging.

### PMP70 immunofluorescence microscopy

Fixed cells were quenched with 50 mM NH_4_Cl for 10 min, permeabilized with 0.1% saponin (Sigma S4521) in PBS for 10 min, and blocked by incubation with 10% FBS in PBS for 30 min. The cells were then stained with anti-PMP70 antibodies (Sigma, SAB4200181) for 1 h and Alexa Fluor 568-conjugated secondary antibodies (Thermo Fisher, A11004) for 30 min. Prior to each antibody incubation, the cells were washed with 0.1% saponin in PBS. Cells mounted with Mowiol/DABCO (Calbiochem 475904/Sigma D2522) were imaged with a confocal Leica Stellaris 8 inverted microscope using × 63 HC PL APO CS2 oil objective NA 1.40.

### Differentiation of hESCs into embryoid bodies

hESCs were split into small clumps and plated on low attachment dishes (Corning) in embryoid bodies (EBs) culture medium (DMEM/F12, Life Technologies) containing 20% KnockOut Serum Replacement (Life Technologies), 0.0915 mM 2-mercaptoethanol (Life Technologies), and 1 × Non-essential Amino Acids (Life Technologies)) to allow EB formation. The EB culture medium was supplemented overnight with 5 µM ROCK inhibitor (Y-27632, Selleckchem) after the initial plating to improve cell viability. Medium was changed every other day. EBs were grown in suspension for 14 days, after which they were plated on matrigel-coated cell culture dishes/coverslips. EBs were allowed to form outgrowths for 7 days.

### Preparing EBs samples for WB, IF staining and live-cell imaging

The hESCs cell-derived EBs were collected on day 21 of differentiation with or without 0.5 µM pico_cvxIAA. For WB, EBs were grown on 35-mm dishes. Medium was removed, and cells were washed once with 1 mL PBS. EBs were detached with a cell scraper, collected into a 1.5-mL Eppendorf tube, and centrifuged at 300 × *g* for 3 min. The supernatant was removed, and cells were lysed in RIPA buffer for WB analysis. For immunocytochemistry, EBs were grown on Matrigel-coated coverslips. Medium was removed, and cells were washed once with DMEM/F12 and once with PBS. Cells were fixed with 2% PFA for 15 min. For live-cell imaging, EBs were plated on Matrigel-coated ibidi plate and processed for imaging.

### Statistics and reproducibility

GraphPad Prism 9 (GraphPad Software, Inc.) was used to generate graphs. Quantitative data are presented as mean ± S.D. Results were validated in at least two cell lines for each endogenous target. Statistical analyses were performed using GraphPad Prism 9. Overview of all the statistical comparisons is shown in Additional file 4: Table S6. In short, data was tested for normal distribution. For normal distributed data, parametric tests were used. For non-normal distributed data, non-parametric tests were used. For comparisons of 2 groups, Student’s *t* test was performed. For statistical comparisons of > 2 groups, one-way or two-way ANOVA were used. N.s.: non-significant, * < 0.05, ** < 0.01, *** < 0.005, **** < 0.001.

### Supplementary Information

**Additional file 1: Note S1-3**, **Fig. S1-9** and **Data S1.** **Note S1.** Comparison of different AID components. **Note S2.** Optimization of coIN and HDR enhancers. **Note S3.** Comparison of Sh_ble and BSD as S2. **Fig. S1.** Comparison of AID components. **Fig. S2.** Optimization of coIN for one-step generation of AID cells. **Fig. S3.** Test of different HDR enhancers in coIN. **Fig. S4.** Generation of AID clones with HiHo-AID2 in A431 cells with Sh_ble as S2 or without S2. **Fig. S5.** WB and functional analysis of the A431 degron cell lines generated with HiHo-AID2. **Fig. S6.** Evaluation of coIN and HDR enhancers in other human cancer cell lines. **Fig. S7.** WB and functional analysis of A549, U2OS and HEK293A degron cell lines generated with HiHo-AID2. **Fig. S8.** Generation of AID hESCs with a conventional procedure and immunostainings of AID hESC-derived embryoid bodies. **Fig. S9.** On-target insertions in HiHo-AID2 hESCs. Data S1. Analysis of HiHo-AID2 cell lines using genomic PCR.

**Additional file 2: Table S1.** Overview of whole genome sequencing of HiHo-AID2 hESCs.

**Additional file 3: Table S2-5. Table S2.** Summary of sequences included. **Table S3.** sgRNA sequences. **Table S4.** PCR primers for genotyping PCR. **Table S5.** Arm-spanning primers for genotyping PCR.

**Additional file 4: Table S6.** Overview of statistical analyses.

**Additional file 5.** Review history.

## Data Availability

Vectors enabling HiHo-AID2 will be available via Addgene (Plasmid #216243–216251). RNAseq data was deposited to the NCBI Gene Entology Omnibus database under the accession number: GSE243637 (https://www.ncbi.nlm.nih.gov/geo/query/acc.cgi?acc=GSE243637) [62]. Whole Genome Sequencing data was deposited to the NCBI Sequence ReadArchive (SRA) data repository under the accession number: PRJNA1050771 (https://www.ncbi.nlm.nih.gov/sra/?term=PRJNA1050771) [63].
