# Supplementary material for: HiHo-AID2: boosting homozygous knock-in efficiency enables robust generation of human auxin-inducible degron cells
Source: Genome Biol. 2024 Feb 26;25:58. doi: 10.1186/s13059-024-03187-w (PMC10895734; doi:10.1186/s13059-024-03187-w)
Supplement: Supplementary file 1 — Additional file 1: Note S1-3, Fig. S1-9 and Data S1. Note S1. Comparison of different AID components. Note S2. Optimization of coIN and HDR enhancers. Note S3. Comparison of Sh_ble and BSD as S2. Fig. S1. Comparison of AID components. Fig. S2. Optimization of coIN for one-step generation of AID cells. Fig. S3. Test of different HDR enhancers in coIN. Fig. S4. Generation of AID clones with HiHo-AID2 in A431 cells with Sh_ble as S2 or without S2. Fig. S5. WB and functional analysis of the A431 degron cell lines generated with HiHo-AID2. Fig. S6. Evaluation of coIN and HDR enhancers in other human cancer cell lines. Fig. S7. WB and functional analysis of A549, U2OS and HEK293A degron cell lines generated with HiHo-AID2. Fig. S8. Generation of AID hESCs with a conventional procedure and immunostainings of AID hESC-derived embryoid bodies. Fig. S9. On-target insertions in HiHo-AID2 hESCs. Data S1. Analysis of HiHo-AID2 cell lines using genomic PCR. [file 13059_2024_3187_MOESM1_ESM.pdf]

Li et al. HiHo-AID2: boosting homozygous knock-in efficiency enables robust generation of human auxin-inducible degron cells

Additional file 1: Note S1-3, Fig. S1-9 and Data S1.

Note S1: Comparison of different AID components.

Several significant pitfalls have been described regarding the AID technique, including basal degradation before induction, inefficient inducible degradation and high inducer concentration (500 μM IAA). Substantial improvements in AID technique were recently published [8,9,13]. Systematic comparisons of the available options to improve the performance of AID were conducted here.

We first compared the different auxin receptors with overexpressed BSCL2-miniIAA7-mEGFP, a substrate that was shown to be sensitive to basal degradation and rapidly degraded upon induction [8] **(Fig. S1a-c)**. As reported, the auxin receptor *Os*TIR1 caused severe basal degradation and this issue could be circumvented by using *At*AFB2 or *Os*TIR1 F74 mutants **(Fig. S1a-c)** [8,9]. The new *At*AFB2(F74A) mutant, like *Os*TIR1(F74G), showed no basal degradation and required 100-1,000 fold lower concentration of the engineered inducers cvx_IAA and pico_cvxIAA compared to IAA for rapid induction of degradation **(Fig. S1d-g)**. It has been reported that the F74G or F74A mutation lost binding to IAA, as substitution of the bulky F amino acid to the small A or G widen the IAA binding pocket [11]. We thus envisage that basal degradation of *Os*TIR1 could be caused by an unknown endogenous ligand analogous to IAA with low activity that bridge the substrate to SCF^{TIR1}. F74G or F74A mutant with widened binding pocket would thus loose the binding to this unknown ligand to reverse the basal degradation [11]. One the other hand, the enhanced sensitivity of engineered inducers might partly result from their higher binding affinity to the F74A and F74G mutants [12]. Moreover, the engineered cvxIAA and pico_cvxIAA would increase membrane permeability due to

their enhanced hydrophobicity compared to IAA that has poor membrane permeability at natural pH [64].

*Os*TIR1(F74G) behaved as *At*AFB2(F74A) in the test, while *Os*TIR1(F74A) showed residual amount of basal degradation but had a 10-fold higher sensitivity to pico_cvxIAA **(Fig. S1a-f)**. *Os*TIR1(F74A) might thus be beneficial when the ligand concentration is limiting, such as in certain *in vivo* applications.

Rapid inducible degradation with AID can be reversed by simply washing out the inducers. We further compared the different combinations regarding the kinetics of washout after induction of rapid degradation. *At*AFB2(F74A) treated with 5 µM cvx_IAA, and *At*AFB2 with 500 µM IAA, showed the best reversibility **(Fig. S1h-i)**. Noticeably, pico_cvxIAA showed poor reversibility, likely due to the retention of residual inducer in the cells after washout and/or the high sensitivity of the auxin receptors to pico_cvxIAA **(Fig. S1h-i)**. Thus, *At*AFB2(F74A) with 5 µM cvx_IAA show no basal degradation, rapid inducible degradation and the best reversibility.

A small tag has less steric hindrance and lower impact on the target protein functions. Previous results show that the fusion of other tags to the C-terminus of miniIAA7 improves inducible degradation [8]. Using GFP as the substrate, we tested the different small tags fused to the C-terminus of miniIAA7 and compared them to miniAID. Interestingly, the miniIAA7-3xFlag showed minimal impact on EGFP stability before induction and the fastest inducible degradation in comparison to miniAID, miniIAA7, and other small miniIAA7 fusion tags tested **(Fig. S1k-l)**. These results emphasize that the choice of fusion at the C-terminus of miniIAA7 may have a major impact on both the target protein stability and its rapid inducible degradation. In addition, use of a suboptimal AID degron tag fusion when comparing with other degron tagging technologies could lead to biased conclusions [65].

We also noticed that the miniIAA7 degron was incorrectly used in the previous literature, resulting in an erroneous conclusion [9]. Based on these results, the miniIAA7-3xFlag was chosen as the small degron tag in the current paper.

High concentration of IAA (500 μM) is challenging to apply *in vivo* and raises the concern of side effects in cultured cells. We used RNA-seq to obtain an overview of at the side effects caused by treatment with different inducers at their effective concentrations in A431 cells. Pico_cvxIAA (0.5 μM) showed negligible off-target activity compared to cvxIAA (5 μM) and IAA (500 μM) **(Fig. S1m-n)**. Interestingly, all the 6 targets (MT-ND2, MT-ND3, MT-ND4L, MT-ND6, MT-ATP6 and MT-ATP8) upregulated by pico_cvxIAA treatment are annotated as mitochondrial proteins and the same targets were not changed in cvxIAA and IAA treated groups **(Fig. S1m-n)**. It is possible that pico_cvxIAA with higher hydrophobicity might accumulate at higher local concentration in mitochondria. With IAA, FABP4 was upregulated more than 8-fold after 24 h treatment, which was further confirmed in WB and not observed with the other inducers **(Fig. S1m-n)**. It should be noted that the lower concentration of the inducer is likely the main reason for lower off-target effects, as cxv_IAA and pico_cvxIAA, but not IAA, at concentration above 50 μM showed clear cell toxicity (data not shown). We thus used 0.5 μM pico_cvxIAA as the inducer of proteolysis for all the subsequent experiments. For experiments that rely on inducer washout, 5 μM of cvxIAA is recommended as discussed above **(Fig. S1h-i).** We would also suggest that in case lower inducer concentration is critical, such as for some *in vivo* applications, *Os*TIR1(F74A) would be a good option that requires 10-fold lower pico_cvxIAA concentration for effective inducible degradation.

Note S2: Optimization of coIN and HDR enhancers.

Simultaneous introduction of two independent genomic modifications results in high coincidence in the same cells, provided that they are mediated by a similar DNA repair pathway (HDR or NHEJ)

[28,29]. CoIN, or co-selection, uses this phenomenon to effectively enrich cells with both genomic modifications. The mechanism was harnessed here for one-step introduction of two AID components. Degron tagging was mediated by HDR. An HDR-mediated *AAVS1* safe harbor integration system (2 plasmids) was thus chosen to overexpress the auxin receptor *At*AFB2(F74A)-mCherry (**Fig. S2a)**. After selection with puromycin, the majority of cells expressed *At*AFB2(F79A)-mCherry (>99%) and integration was effectively mediated by HDR (sgAAVS1) instead of random integration (sgCtrl) or homology-independent targeted insertion (sgSEC61B) [53], especially in the presence of HDR enhancers **(Fig. S2b)**.

For degron-tagging through coIN, an endogenous degron-GFP tagging pair (2 plasmids) was co-transfected with the *AAVS1* integration system **(Fig. S2c-d)**. *At*AFB2(F74A)-mCherry expressing cells were selected with puromycin and the efficiency of degron-GFP tagging in the selected cells was measured by FACS. The efficiency of endogenous tagging increased with a higher amount of endogenous tagging plasmids. Tagging efficiency reached a plateau at 1:3 ratio and a further increase of the ratio decreased the number of puromycin resistant cells **(Fig. S2d-e)**. The ratio of 1:3 was thus chosen for effective degron-tagging with a high yield of puromycin selected cells. *At*AFB2(F74A)-mCherry expression was commonly close to 100% and will not be described separately below.

The efficiencies of endogenous tagging through HDR were determined by the interplay between the main DNA Double-Strand Break (DSB) repair pathways, including Homology-Directed Repair (HDR) and Non-Homologous End Joining (NHEJ) [14]. DNA-PK is a critical component of the NHEJ pathway that is recruited by the Ku heterodimer to DNA DSBs to form the DNA-PK complex, which undergoes auto-phosphorylation and activates additional NHEJ factors [19]. 53BP1 (encoded by TP53BP1 in human cells) is another pro-NHEJ factor that limits HDR in part by blocking DNA end resection, which is the rate-limiting step in the initiation of HDR [20]. Thus, inhibitors targeting

DNA-dependent protein kinase (DNA-PK) and 53BP1 were used to increase HDR efficiencies through inhibition of NHEJ.

Several inhibitors with different properties were tested to improve degron-tagging efficiency in coIN. These included M3814 (a small-molecule inhibitor of DNA-PK) [19], i53 (a small-peptide inhibitor of 53BP1) [20], and Cas9-53BP1-DN1S (a Cas9 fusion protein that was designed to inhibit 53BP1 locally at Cas9 cut sites) [21]. In addition, XL413 (a CDC7 inhibitor controlling the cell cycle) was included as it was recently identified in a large screen to improve HDR [22]. Two of the inhibitors, M3814 and i53, effectively increased degron-GFP tagging efficiencies in coIN (7.5-fold increase in GFP levels with 1 μM M3814 and 2.6-fold with i53 overexpression) **(Fig. S3a)**.

Moreover, i53, but not 1 μM M3814, improved degron-GFP tagging efficiency without reducing cell counts **(Fig. S3a)** and i53 reduced the concentration of M3814 needed from 1 μM to 0.25 μM to achieve high tagging efficiency (**Fig. S3b)**. 1 μM M3814 and i53 plus 0.25 μM M3814 were thus chosen for further tests.

Note S3: Comparison of Sh_ble and BSD as S2.

Streptoalloteichus hindustanus bleomycin (Sh_ble) and Blasticidin S deaminase (BSD) genes, that confer resistance to Zeocin and Blasticidin respectively, were initially selected as S2 due to their small size (369 bp and 396 bp) **(Fig. S4a** and **Fig. 2f)**. The P2A-S2 cassettes do not have a promoter and thus will not likely be expressed through random integration. In cells with degron tagging, the selection marker is transcribed under the control of an endogenous promoter and is translated at 1:1 ratio with the endogenous target through P2A self-cleavage [30].

Single clones were initially isolated as in **Fig. 1c** without HDR enhancer. Using Sh_ble as S2,

homozygous clones were effectively enriched for 2 targets (SAC1 and DHC1) **(Fig. S4b)**. However, further tests showed that low-expressing BSCL2 clones died out and high-expressing ones, such as SEC61B, LMNA and MYH9 [8], had a high number of heterozygous clones **(Fig. S4b)**. Moreover, SAC1 and DHC1 clones grew more slowly than the high-expressing clones during Zeocin selection with clear indications of cell stress (data not shown). Further tests showed that HDR enhancer M3814 (1 μM) substantially improved the efficiency of homozygous tagging for the high-expressing targets with Sh_ble as S2 **(Fig. S4b)**. These results, together with results using BSD as S2 **(Fig. 2i)**, consistently show that HDR enhancers substantially improved homozygous degron tagging efficiencies in one-step generation of AID cells.

BSD catalyzes the degradation of Blasticidin [30] while Sh_ble binds stoichiometrically to Zeocin [31]. The catalytic feature of BSD might explain its higher sensitivity to enrich the low-expressing BSCL2 clones **(Fig. 2i)**. The choice of S2 depends on the drug sensitivity of the cell line and the expression level of the target protein.

References

64. Yang Y, Hammes UZ, Taylor CG, Schachtman DP, Nielsen E. High-Affinity Auxin Transpor by the AUX1 Influx Carrier Protein. Current Biology. 2006;16(11):1123-1127. Doi:10.1016/j.cub. 2006.04.029

65. Bondeson DP, Mullin-Bernstein Z, Oliver S, et al. Systematic profiling of conditional degron tag technologies for target validation studies. Nature Communications 2022;13(1):1-11. Doi:10. 1038/s41467-022-33246-4

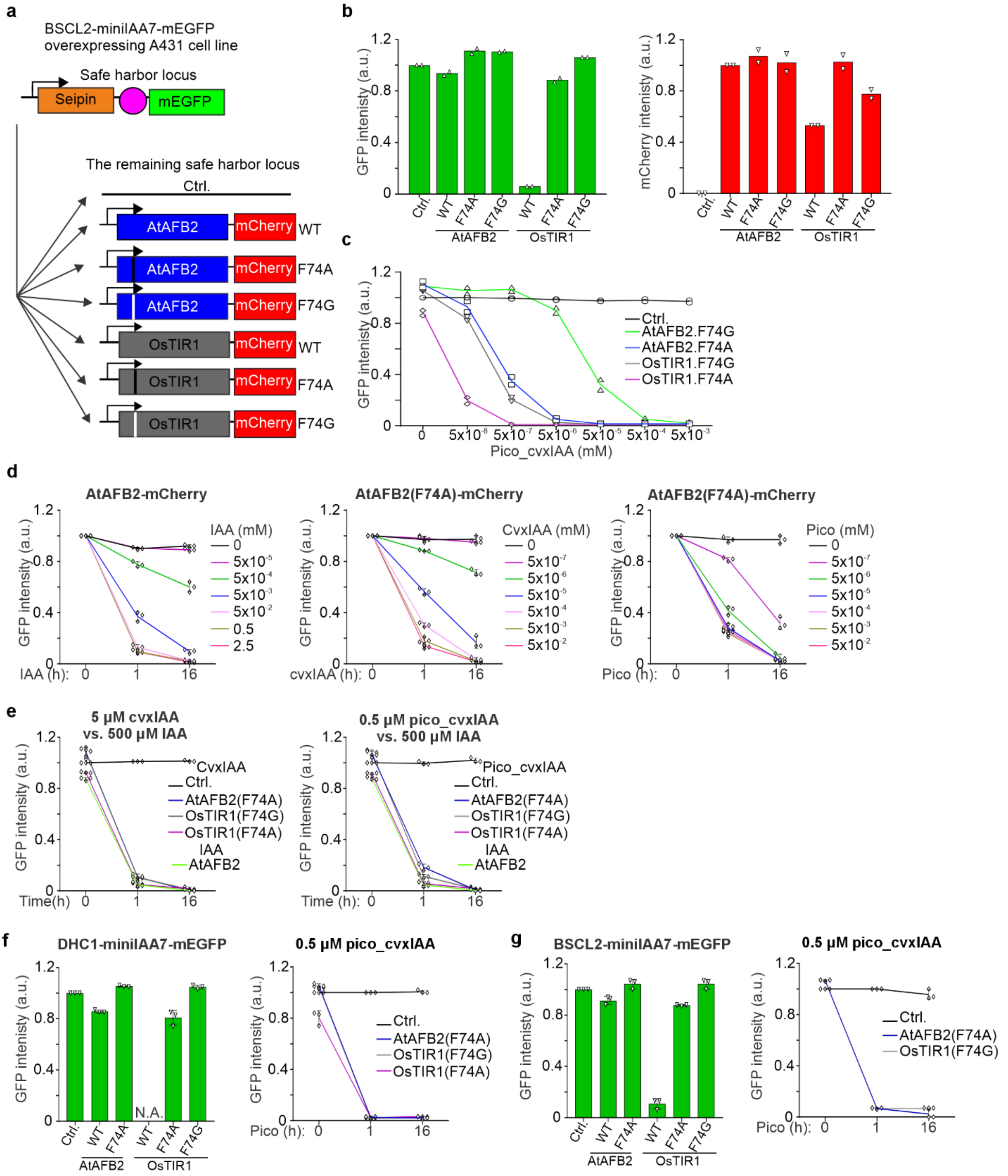
a
BSCL2-miniIAA7-mEGFP
overexpressing A431 cell line
Safe harbor locus
Seipin
mEGFP
The remaining safe harbor locus
Ctrl.
AtAFB2
mCherry
WT
F74A
F74G
OsTIR1
b
GFP intenisty (a.u.)
mCherry intenisty (a.u.)
Ctrl.
WT
F74A
F74G
AtAFB2
OsTIR1
c
GFP intenisty (a.u.)
Ctrl.
AtAFB2.F74G
AtAFB2.F74A
OsTIR1.F74G
OsTIR1.F74A
Pico_cvxIAA (mM)
d
AtAFB2-mCherry
AtAFB2(F74A)-mCherry
GFP intensity (a.u.)
IAA (mM)
CvxIAA (mM)
Pico (mM)
IAA (h):
cvxIAA (h):
Pico (h):
e
5 μM cvxIAA
vs. 500 μM IAA
0.5 μM pico_cvxIAA
vs. 500 μM IAA
CvxIAA
Pico_cvxIAA
Ctrl.
AtAFB2(F74A)
OsTIR1(F74G)
OsTIR1(F74A)
IAA
AtAFB2
Time(h)
Time (h):
f
DHC1-miniIAA7-mEGFP
0.5 μM pico_cvxIAA
N.A.
g
BSCL2-miniIAA7-mEGFP

Fig. S1

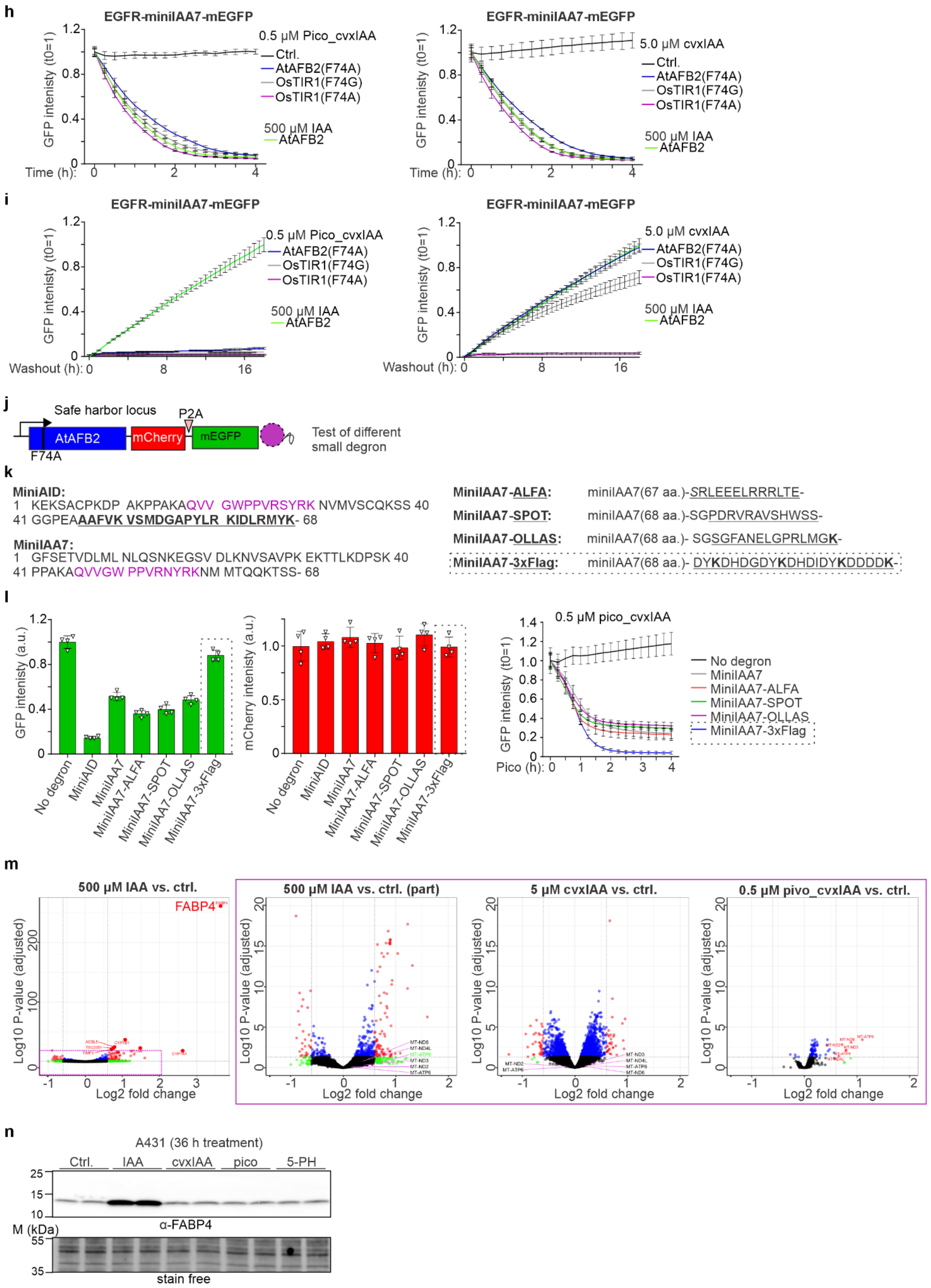
h
EGFR-miniIAA7-mEGFP
GFP intenisty (t0=1)
Time (h):
0.5 µM Pico_cvxIAA
Ctrl.
AtAFB2(F74A)
OsTIR1(F74G)
OsTIR1(F74A)
500 µM IAA
AtAFB2
5.0 µM cvxIAA
i
Washout (h):
j
Safe harbor locus
P2A
AtAFB2
F74A
mCherry
mEGFP
Test of different
small degron
k
MiniAID:
1 KEKSACPKDP AKPPAKAQVV GWPPVRSYRK NVMVSCQKSS 40
41 GGPEAAAFVK VSMDGAPYLR KIDLRMYK- 68
MiniIAA7:
1 GFSETVDLML NLQSNKEGSV DLKNVSAVPK EKTTLKDPSK 40
41 PPAKAQVVGW PPVRNYRKNM MTQQKTSS- 68
MiniIAA7-ALFA: miniIAA7(67 aa.)-SRLEEELRRRLTE-
MiniIAA7-SPOT: miniIAA7(68 aa.)-SGPDRVRAVSHWSS-
MiniIAA7-OLLAS: miniIAA7(68 aa.)- SGSGFANELGPRLMGK-
MiniIAA7-3xFlag: miniIAA7(68 aa.)- DYKDHDGDYKDHDIDYKDDDDK-
l
GFP intenisty (a.u.)
mCherry intenisty (a.u.)
No degron
MiniAID
MiniIAA7
MiniIAA7-ALFA
MiniIAA7-SPOT
MiniIAA7-OLLAS
MiniIAA7-3xFlag
0.5 µM pico_cvxIAA
Pico (h):
m
500 µM IAA vs. ctrl.
FABP4
500 µM IAA vs. ctrl. (part)
5 µM cvxIAA vs. ctrl.
0.5 µM pivo_cvxIAA vs. ctrl.
Log10 P-value (adjusted)
Log2 fold change
n
A431 (36 h treatment)
Ctrl.
IAA
cvxIAA
pico
5-PH
M (kDa)
α-FABP4
stain free

Fig. S1.2

Fig. S1: Comparison of AID components.

a, scheme of the genomic modifications in cells to compare different auxin receptors using overexpressed BSCL2-miniIAA7-mEGFP as substrate.

b, basal levels of BSCL2-miniIAA7-mEGFP and expression levels of mCherry tagged auxin receptors before induction, analyzed by FACS. N=2 independent experiments.

c, levels of BSCL2-miniIAA7-mEGFP after 16 h treatment of pico_cvxIAA at indicated concentrations in cells expressing different auxin receptors, analyzed by FACS. N=2 independent experiments.

d, levels of BSCL2-miniIAA7-mEGFP after treatment with IAA (left) or engineered IAA (middle and right) for 0, 1 and 16 h at indicated concentrations, analyzed by FACS. N=3 independent experiments.

e, levels of BSCL2-miniIAA7-mEGFP after treatment with 500 μM IAA (left and right), 5 μM cvxIAA (left) or 0.5 μM pico_cvxIAA (right) for 0, 1 and 16 h in cells expressing different auxin receptors, analyzed by FACS. N=3 independent experiments.

f, basal levels (left) and inducible degradation (right) of endogenous DHC1-miniIAA7-mEGFP in cells expressing different auxin receptors, analyzed by FACS. N=3 independent experiments.

g, basal levels (left) and inducible degradation (right) of endogenous BSCL2-miniIAA7-mEGFP in cells expressing different auxin receptors, analyzed by FACS. N=3 independent experiments.

h, rapid inducible degradation of endogenous EGFR-miniIAA7-mEGFP (heterozygous tagging) after treatment with 500 μM IAA (left and right), 5 μM cvxIAA (left) or 0.5 μM pico_cvxIAA (right) in cells expressing different auxin receptors, analyzed by live cell imaging. N=4 fields. Representative of 2 independent experiments.

i, recovery from washout of endogenous EGFR-miniIAA7-mEGFP (heterozygous tagging) in cells after 16 h treatment with 500 μM IAA (left and right), 5 μM cvxIAA (left) or 0.5 μM pico_cvxIAA (right). N=4 fields. Representative of 2 independent experiments.

j, scheme of genetic modifications to compare different small degron tags. P2A: self-cleavage peptide.

k, amino acid sequences of different small degrons. Magenta: core sequences for the auxin receptor binding; bold and underlined: sequence derived from the dimerization domain in miniAID; framed: miniIAA7-3xFlag used to establish AID clones.

l, basal levels of mEGFP fusions (left), expression levels of *At*AFB2(F74A)-mCherry (middle), and the inducible degradation of mEGFP fusions (right), analyzed by live-cell imaging. N=4 fields. Representative of 2 independent experiments.

m, volcano plots of RNAseq in A431 wild-type cells treated with different inducers for 24 h compared to control. Full plot of IAA treated cells is shown on the left with 7 targets out of the range for comparison (magenta frame); FABP4 highlighted for WB analysis. No target is out of this range for cells treated with the other 2 inducers. Threshold for different colors: fold change at >1.5 or <0.5, P value at 0.05.

n, WB analysis of FABP4 in A431 cells treated with different inducers for 36 h. 500 µM IAA, 5.0 µM cvxIAA, 0.5 µM pico_cvxIAA or 1 µM 5-PH was used.

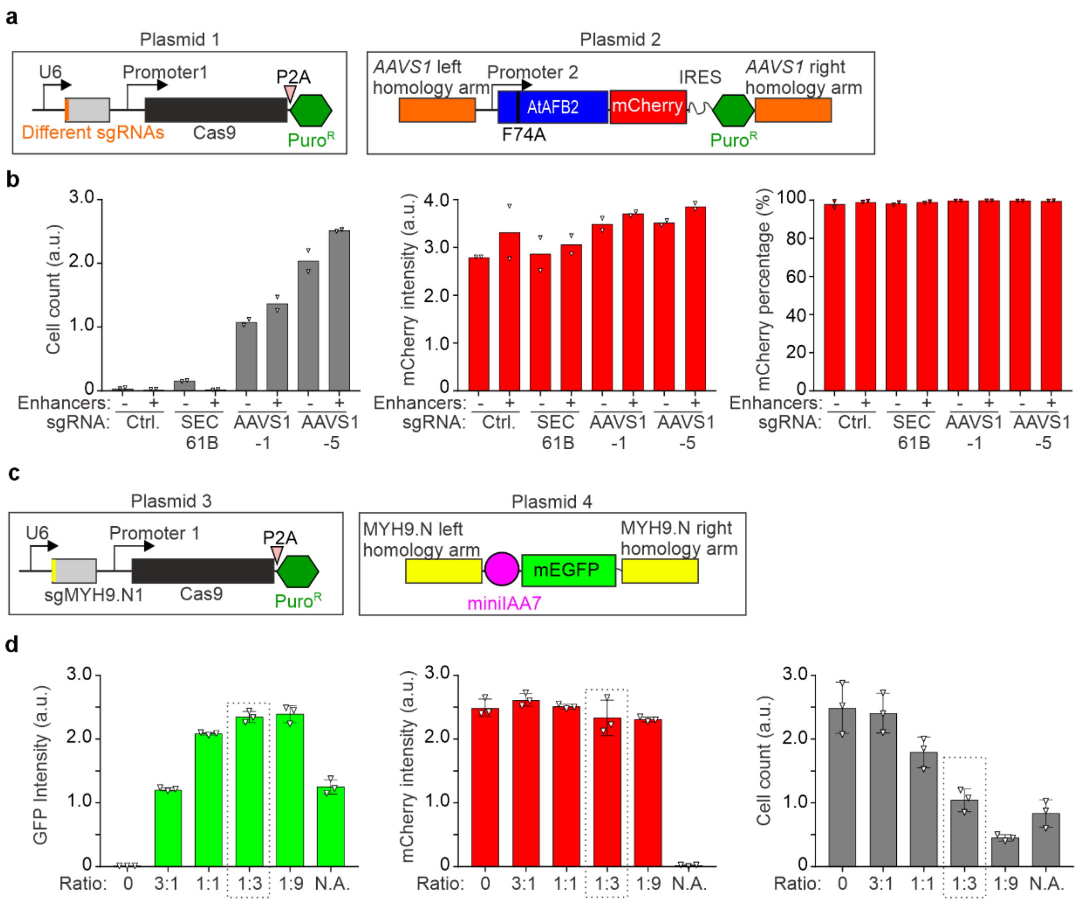

e

***AAVS1*:MYH9 Ratio=1:3**
(for a 12-well, total of 1 µg plasmids)
Plasmid 1 (µg): 0.1
Plasmid 2 (µg): 0.15
Plasmid 3 (µg): 0.3
Plasmid 4 (µg): 0.45

Fig. S2

Fig. S2: Optimization of coIN for one-step generation of AID cells.

a, scheme of the plasmid pair used in (b) to test the HDR-mediated *AAVS1* integration efficiency.

b, cell count (left), mCherry intensity (middle) and percentage of mCherry positive cells (right) in cell pools generated with indicated sgRNAs. Cells were transfected with Plasmid 1 and plasmid 2 at 2:3 ratio (µg: µg). Enhancers: i53 plus 0.25 µM M3814 as HDR enhancers (see Fig. S3 for a description of HDR enhancers).

c, scheme of the plasmid pair used in (d) to test HDR-mediated endogenous miniIAA7-mEGFP tagging efficiency of MYH9.

d, levels of GFP (MYH9 tagging efficiency), mCherry (*At*AFB2(F74A) expression level), and cell counts (*AAVS1* integration efficiency) in cell pools generated with indicated ratio of *AAVS1*: MYH9 tagging plasmid pairs (plasmid 1+2: plasmid 3+4). Wild-type A431 cells were co-transfected with the 4 plasmids and selected for stable puromycin-resistant cell pools before analysis (except for group N.A.). N.A. no *AAVS1* integration plasmid pair.

e, amount of 4 plasmids used at 1:3 ratio (*AAVS1*: endogenous loci) for transfection of a 12-well of cells.

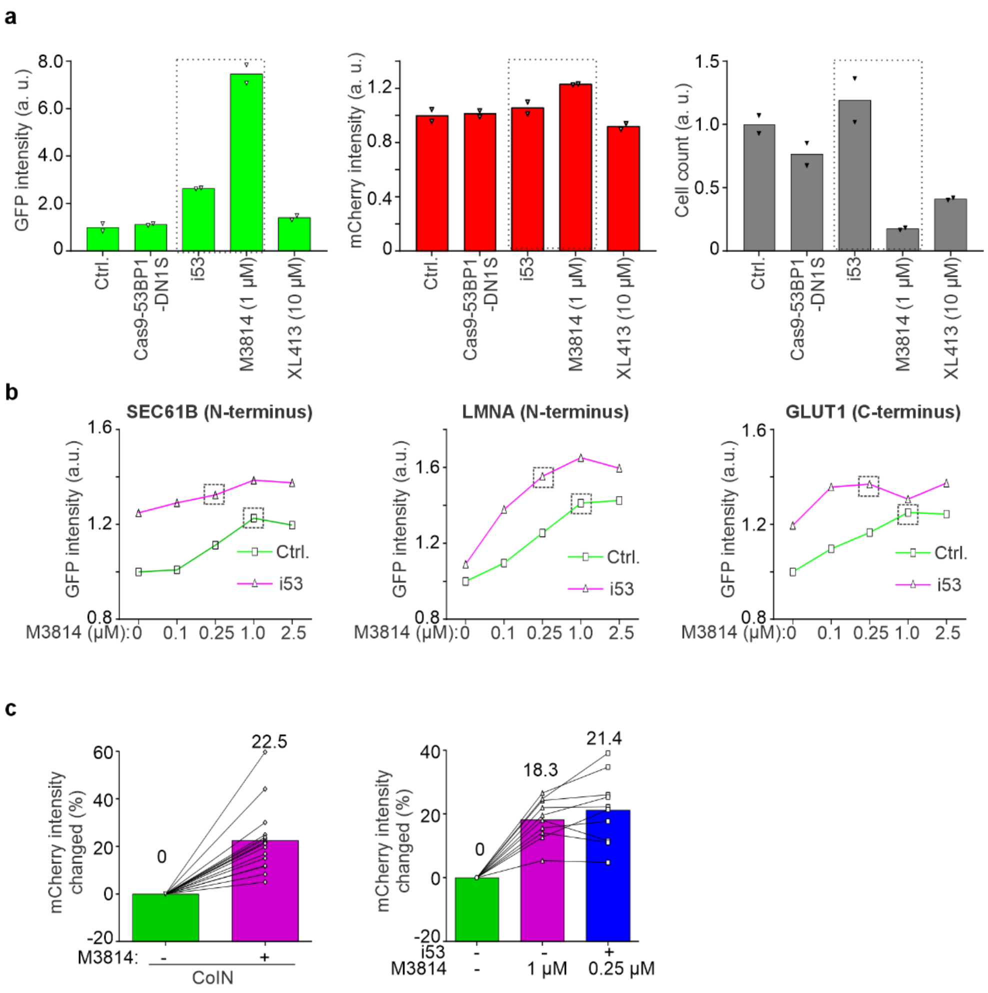

Fig. S3: Test of different HDR enhancers in coIN.

a, levels of GFP (MYH9 tagging efficiency), mCherry (*At*AFB2(F74A) expression level), and cell count (cell toxicity) in stable cell pools using different HDR enhancers. Stable cell pools were generated by coIN with *AAVS1*:MYH9 at 1:3 ratio. N=2 technical repeats. Black frames indicate M3814 and i53 used for further analysis.

b, endogenous tagging efficiencies of SEC61B (left), LMNA (middle) and GLUT1 (right) in cell pools generated by coIN with or without i53 expression plus different concentration of M3814. Frames indicate 1 µM M3814 and i53 plus 0.25 µM M3814 chosen for later experiments.

c, mCherry expression levels with indicated HDR enhancers. Results from the same experiment as **Fig. 2 c** and **e.** N=16 (left) and 10 (right). Numbers above columns indicate mean values; lines link the same endogenous tagging pairs.

Fig. S3

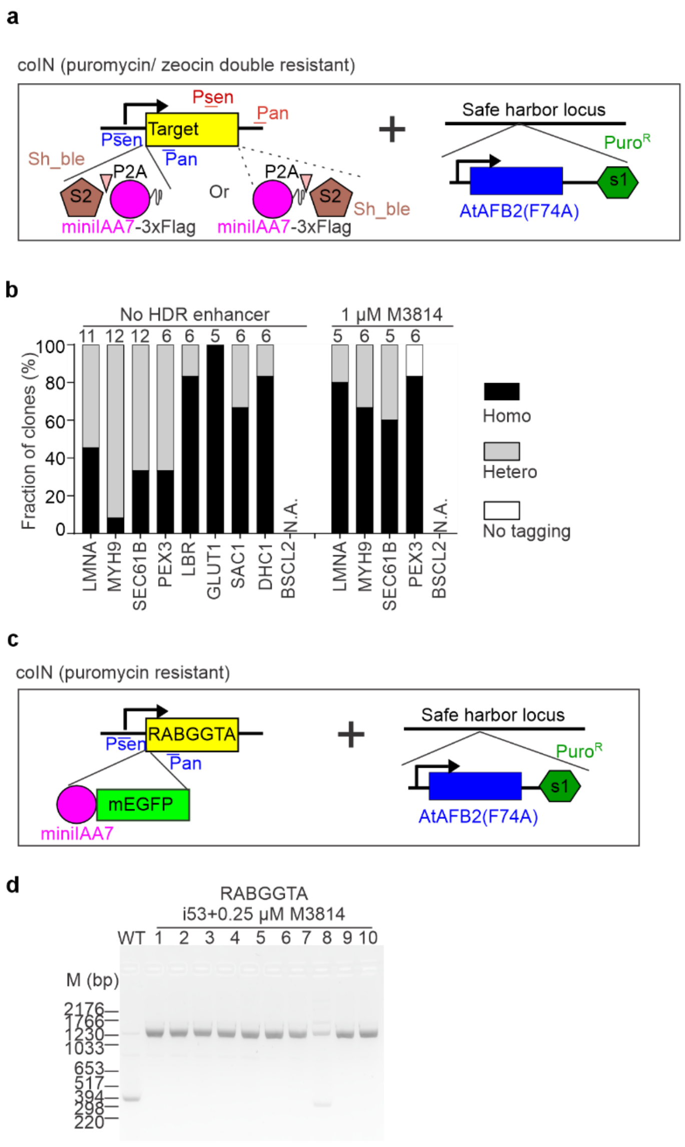

Fig. S4: Generation of AID clones with HiHo-AID2 in A431 cells with Sh_ble as S2 or without S2.

a, scheme of the genomic modifications in one-step generation of AID cells using Sh_ble as S2.

b, statistics of genotyping PCR results for 9 targets without HDR enhancer, and 5 targets with 1 μM M3814 as the HDR enhancer . N.A. not available due to cell death after Zeocin (S2) selection . Numbers indicate total amount of clones analyzed.

c, scheme of the genomic modifications in one-step generation of AID cells targeting RABGGTA without S2.

d, genotyping PCR results for RABGGTA clones generated without S2. WT: wild-type.

Fig. S4

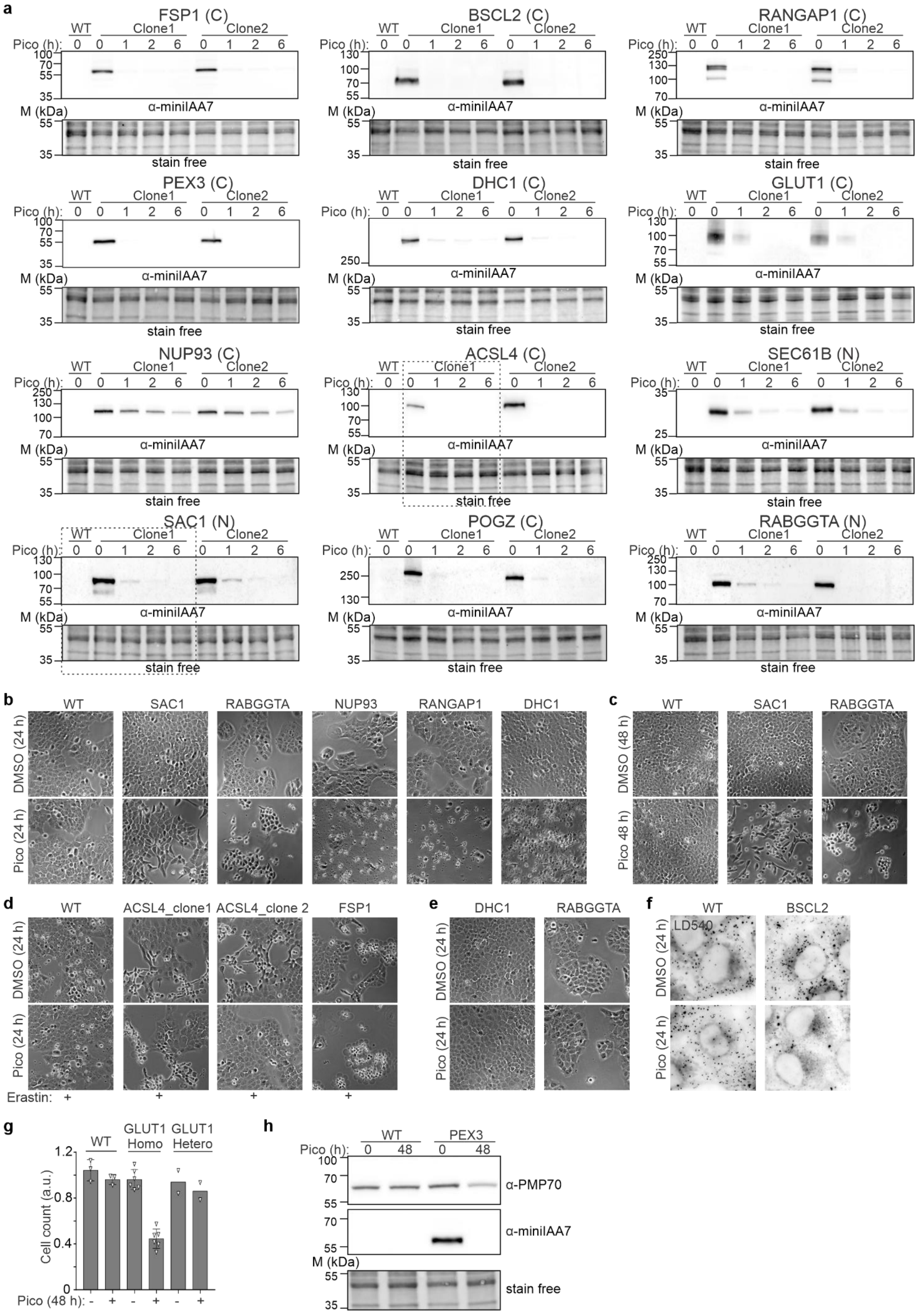
a
FSP1 (C)
BSCL2 (C)
RANGAP1 (C)
PEX3 (C)
DHC1 (C)
GLUT1 (C)
NUP93 (C)
ACSL4 (C)
SEC61B (N)
SAC1 (N)
POGZ (C)
RABGGTA (N)
WT
Clone1
Clone2
Pico (h):
M (kDa)
α-miniIAA7
stain free
b
WT
SAC1
RABGGTA
NUP93
RANGAP1
DHC1
DMSO (24 h)
Pico (24 h)
c
DMSO (48 h)
Pico 48 h)
d
ACSL4_clone1
ACSL4_clone 2
FSP1
Erastin:
e
f
BSCL2
LD540
g
GLUT1 Homo
GLUT1 Hetero
Cell count (a.u.)
Pico (48 h):
h
PEX3
α-PMP70

Fig. S5

Fig. S5: WB and functional analysis of the A431 degron cell lines generated with HiHo-AID2.

a, WB analysis of inducible degradation with anti-miniIAA7 antibody. 2 homozygous clones identified in gPCR generated with HDR enhancers for each target. Frames highlight the results of SAC1 clone 1 shown in Fig. 2j**,** and ASCL4_clone 1 that might be heterozygously tagged with no clear phenotypic change after induction in (d). N and C in brackets indicate terminus of degron-tagging.

b-c, representative live-cell images showing morphological changes and cell death in homozygous clones after 24 h (b) and 48 h (c) of induction. N=8 (SAC1), 9 (RABGGTA), 6 (NUP93), 5 (DHC1) and 4 (RANGAP1) clones.

d, representative live-cell images showing erastin-induced ferroptosis in homozygous clones after 24 h of induction. FSP1 inhibits while ASCL4 promotes ferroptosis. ASCL4_clone 1 with lower degron-tagged protein level in (a) show no clear phenotypic change. N=2 clones for FSP1.

e, live-cell images of heterozygous clones identified in gPCR showing no clear phenotypic changes after 24 h of induction. He: heterozygous; N=3 (DHC1) and 1 (RABGGTA) clone.

f, live-cell images showing lipid droplets stained by LD540 with lots of small and a few big lipid droplets in BSCL2 degron cells after 24 h induction; 0.2 mM of oleic acid was added during the final 2 h to induce LD formation. N=6 clones.

g, growth of cells analyzed by cell counting after 48 h of induction. Ho: homozygous; He: heterozygous; each data point of a GLUT1 column representing one clone. N=6 (Homo) and 2 (Hetero) clones.

h, WB analysis of peroxisomal membrane protein PMP70 depletion in PEX3 homozygous clones after 48 of induction.

WT: wild-type; pico: 0.5 μM pico_cvxIAA treatment; a.u.: arbitrary unit.

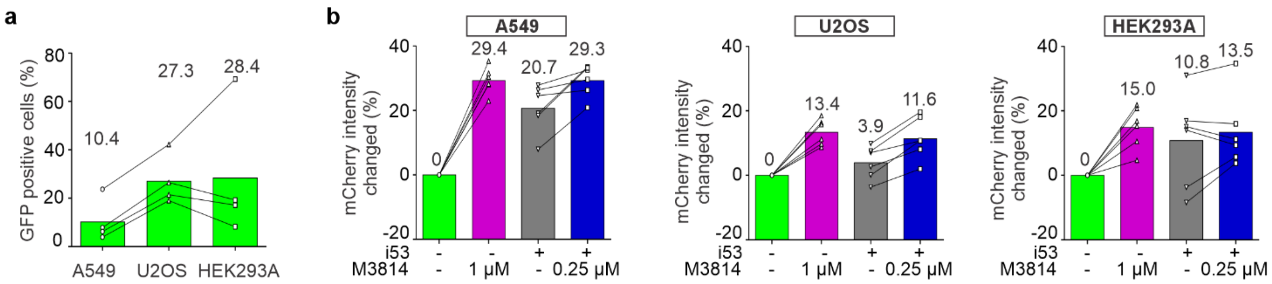

Fig. S6: Evaluation of coIN and HDR enhancers in other human cancer cell lines.

a, percentage of degron-GFP tagging in A549, U2OS and HEK293A cells through conventional tagging without *AAVS1* integration analyzed by FACS. Numbers above columns indicate mean value. Lines link the same endogenous tagging pair. N=4 (2 target proteins with 2 sgRNAs each).

b, mCherry expression levels with indicated HDR enhancers in A549, U2OS and HEK293A cells. Results from the same experiment as **Fig. 3 b, d, f.** Numbers above columns indicate mean values; lines link the same endogenous tagging pairs. N=6 pairs of tagging plasmids.

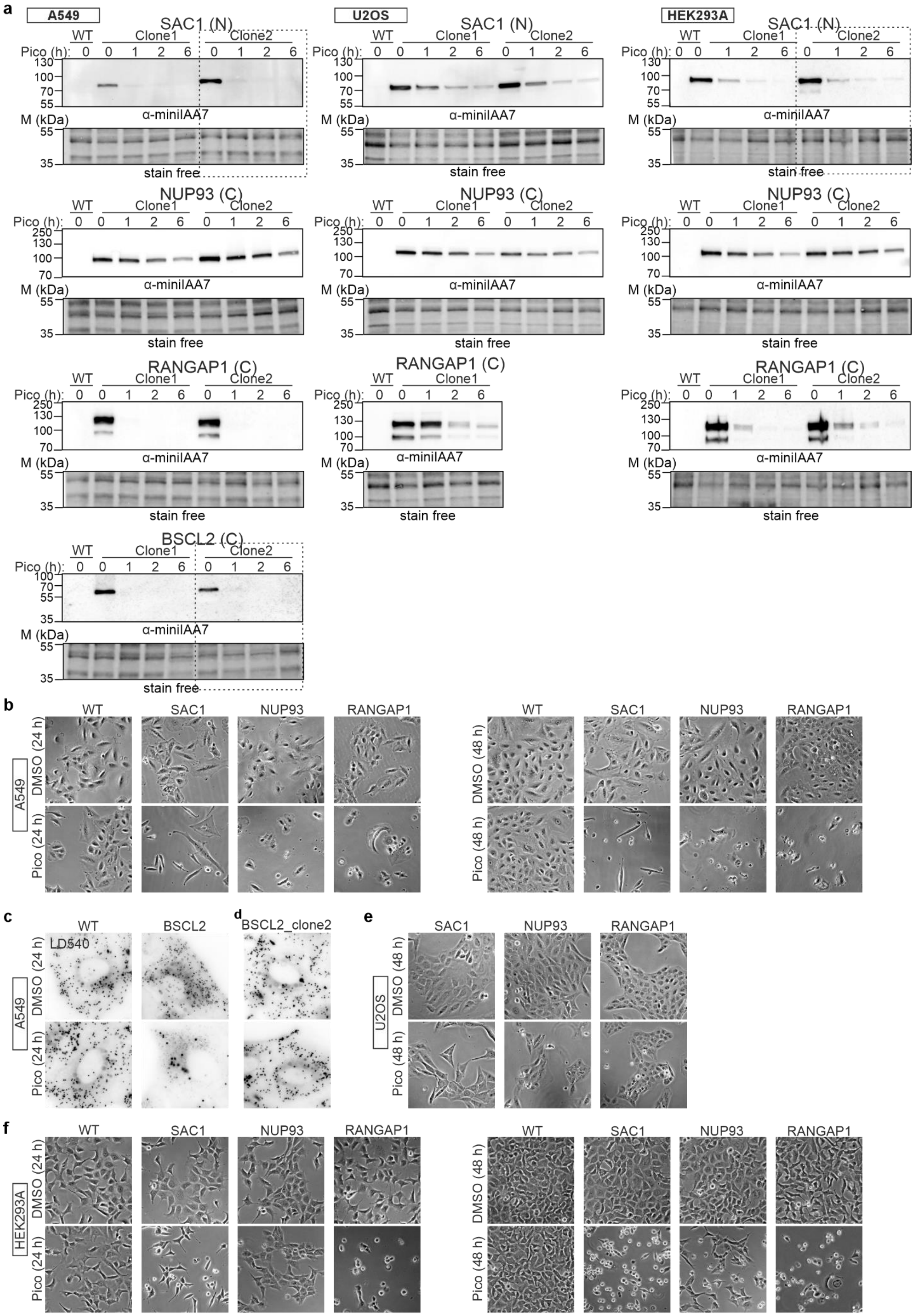
a
A549
SAC1 (N)
WT
Clone1
Clone2
Pico (h): 0 0 1 2 6 0 1 2 6
M (kDa)
130
100
70
55
α-miniIAA7
55
35
stain free
U2OS
HEK293A
NUP93 (C)
250
RANGAP1 (C)
BSCL2 (C)
b
WT
SAC1
NUP93
RANGAP1
DMSO (24 h)
Pico (24 h)
DMSO (48 h)
Pico (48 h)
c
BSCL2
LD540
d
BSCL2_clone2
e
f

Fig. S7

Fig. S7: WB and functional analysis of A549, U2OS and HEK293A degron cell lines generated with HiHo-AID2.

a, WB analysis of inducible degradation with anti-miniIAA7 antibody. Homozygous clones identified in gPCR generated with HDR enhancers. Frames highlight the results of SAC1 shown in Fig. 3h, and BSCL2_clone 2 that might be heterozygously tagged with no clear phenotypic change after induction in (c). N and C in brackets indicate terminus of degron-tagging.

b, live-cell images showing morphological and cell-density changes in homozygous clones after 24 h (left) and 48 h (right) of induction in A549 cells. N=7 (NUP93), 5 (both SAC1 and RANGAP1) clones.

c-d, live-cell images of lipid droplets stained by LD540 showing lots of small with a few big lipid droplets in A549 BSCL2 degron cells after 24 h pico_cvxIAA treatment (c) and BSCL2_clone 2 without clear change (d). 0.2 mM of oleic aicd was added during the final 2 h to induce LD formation. WB of BSCL2_clone 2 shown in (a). N=5 (c) and 1(d) clones.

e, live-cell imaging analysis of morphological changes in homozygous clones after 48 h of induction in U2OS cells.

f, live-cell images showing morphological and cell density changes in homozygous clones after 24 h (left) and 48 h (c) of induction in HEK293A cells. N= 4 (SAC1), 1 (NUP93) and 2 (RANGAP1) clones.

WT: wild-type; pico: 0.5 μM pico_cvxIAA treatment.

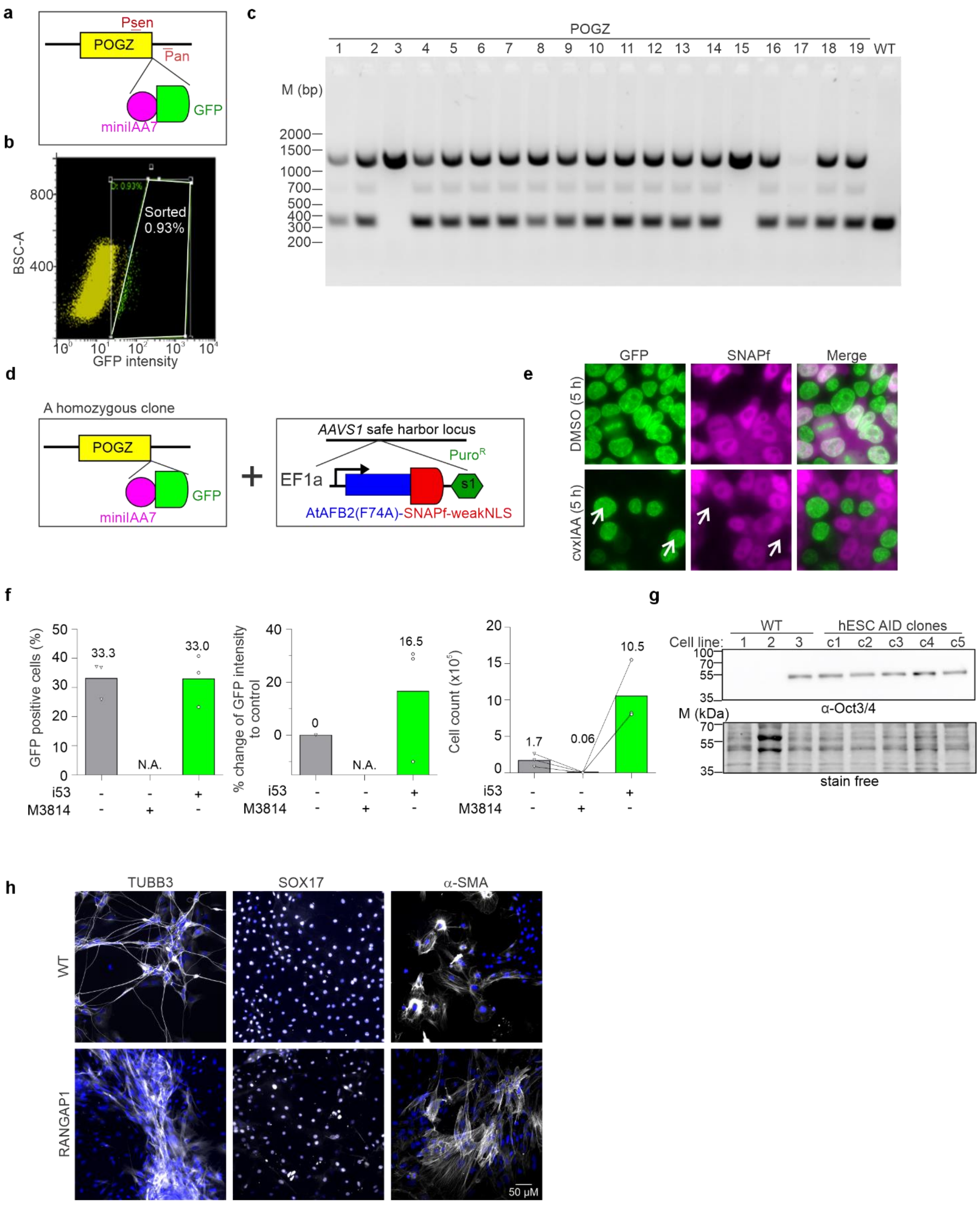

a
Psen
POGZ
Pan
GFP
miniIAA7
b
Sorted 0.93%
BSC-A
GFP intensity
c
POGZ
M (bp)
1 2 3 4 5 6 7 8 9 10 11 12 13 14 15 16 17 18 19 WT
2000
1500
1000
700
500
400
300
200
d
A homozygous clone
POGZ
GFP
miniIAA7
AAVS1 safe harbor locus
PuroR
EF1a
s1
AtAFB2(F74A)-SNAPf-weakNLS
e
GFP
SNAPf
Merge
DMSO (5 h)
cvxIAA (5 h)
f
GFP positive cells (%)
33.3
N.A.
33.0
% change of GFP intensity to control
0
N.A.
16.5
Cell count (x10^5)
1.7
0.06
10.5
i53
M3814
g
WT
hESC AID clones
Cell line: 1 2 3 c1 c2 c3 c4 c5
M (kDa)
α-Oct3/4
stain free
h
TUBB3
SOX17
α-SMA
WT
RANGAP1
50 μM

Fig. S8

Fig. S8: Generation of AID hESCs with a conventional procedure and immunostainings of AID hESC-derived embryoid bodies.

a, scheme showing endogenous POGZ tagging with miniIAA7-mEGFP at C-terminus. Pse and Pan for genomic PCR in (c).

b, FACS plot of analyzing POGZ tagging efficiency. Cells in the white frame (0.93%) were used for single-cell cloning.

c, genotyping PCR identifying POGZ-miniIAA7-mEGFP single-cell clones derived from sorted cells in (b).

d, scheme showing introduction of *At*AFB2(F74A)-SNAPf-weakNLS into a homozygous clone through AAVS1 integration.

e, live cell images showing single-cell clones generated from (d) with or without 5 μM cvx_IAA induction for 5 h. Arrows indicate cells without *At*AFB2(F74A) expression and showing defective POGZ depletion after cvx_picoIAA induction. Representative of 10 clones.

f, graph depicting the percentage of GFP positive cells, the percentual change of single- cell GFP intensity change to control and the cell count (x10^5 cells/ml) of 3 hESC AID clones treated with 0.25 μM M3814 or i53.

g, WB analysis of Oct3/4 expression in hESC clones generated with HiHo-AID2. Wild-type 1-3: A431 (1), hESC-derived neurons (2) and hESCs (3); c1-c5: hESC AID clones targeting NUP93 (c1), RANGAP1 (c2), SAC1 (c3), PEX3 (c4) and BSCL2 (c5). Representative of 2 AID clones for each target.

h, Immunocytochemical detection of tri-lineage differentiation markers for ectoderm (TUBB3), mesoderm (α-SMA), and endoderm (SOX17) in plated EBs derived from hESC-AIDs. Nuclei stained blue. Scale bar, 50 μm.

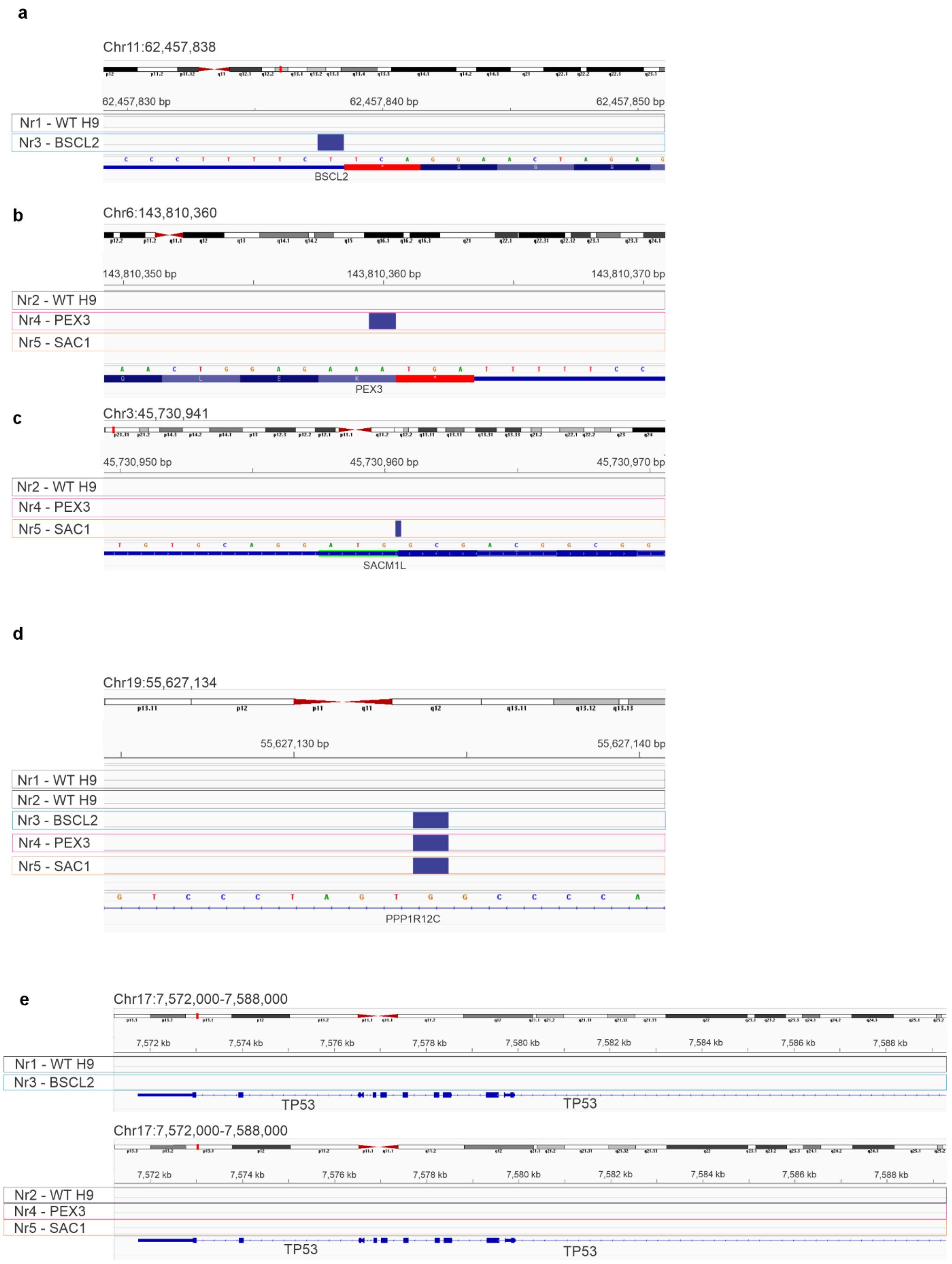

a
Chr11:62,457,838
62,457,830 bp
62,457,840 bp
62,457,850 bp
Nr1 - WT H9
Nr3 - BSCL2
BSCL2
b
Chr6:143,810,360
143,810,350 bp
143,810,360 bp
143,810,370 bp
Nr2 - WT H9
Nr4 - PEX3
Nr5 - SAC1
PEX3
c
Chr3:45,730,941
45,730,950 bp
45,730,960 bp
45,730,970 bp
Nr2 - WT H9
Nr4 - PEX3
Nr5 - SAC1
SACM1L
d
Chr19:55,627,134
55,627,130 bp
55,627,140 bp
Nr1 - WT H9
Nr2 - WT H9
Nr3 - BSCL2
Nr4 - PEX3
Nr5 - SAC1
PPP1R12C
e
Chr17:7,572,000-7,588,000
7,572 kb
7,574 kb
7,576 kb
7,578 kb
7,580 kb
7,582 kb
7,584 kb
7,586 kb
7,588 kb
Nr1 - WT H9
Nr3 - BSCL2
TP53
TP53
Chr17:7,572,000-7,588,000
Nr2 - WT H9
Nr4 - PEX3
Nr5 - SAC1
TP53
TP53

Fig. S9

Fig. S9: On-target insertions in HiHo-AID2 hESCs.

a, WGS data showing the location of the on-target insertion of the miniIAA7-tag at chromosome 11:62,457,838 at the BSCL2 gene of the BSCL2 AID2 hESCs cell line, with Nr1 wild-type (WT) hESCs as control.

b, WGS data showing the location of the on-target insertion of the miniIAA7-tag at chromosome 6:143,810,360 at the PEX3 gene of the PEX3 AID2 hESC line, with Nr2 WT hESCs as control.

c, WGS data showing the location of the on-target insertion of the miniIAA7-tag at chromosome 3:45,730,941 at the SACM1L gene of the SAC1 AID2 hESC line, with Nr2 WT hESCs as control.

d, WGS data showing the location of the on-target insertion of the *At*AFB2(F74A) receptor through AAVS1 safe harbour integration at chromosome 19:55,627,134.

e, WGS data showing no variations in the TP53 gene upon HiHo-AID2 mediated knock-in in hESCs. BSCL2, PEX3 and SAC1 AID2-hESCs compared to their respective parental WT hESC line.

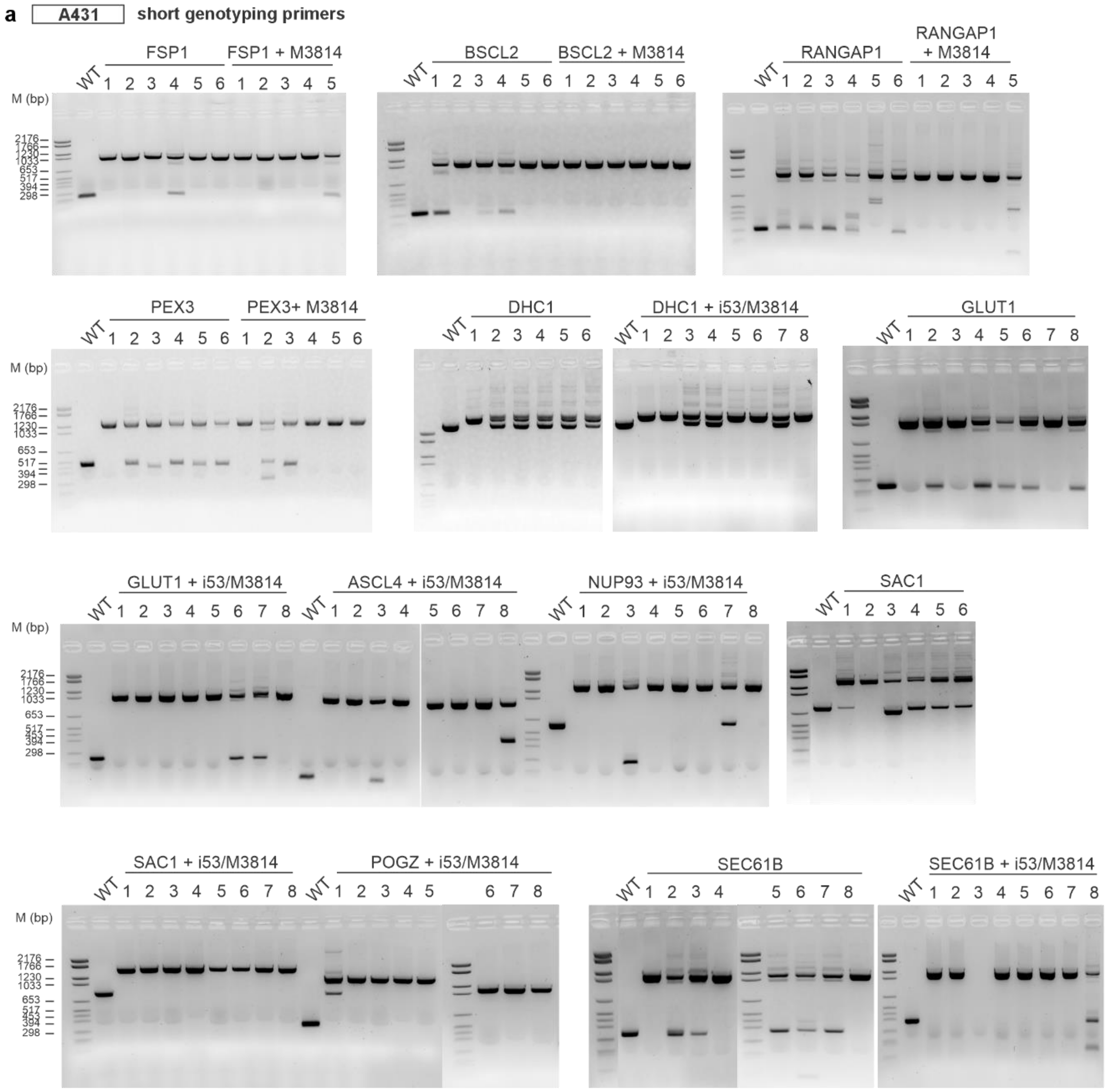
a
A431
short genotyping primers
M (bp)
2176
1766
1230
1033
653
517
453
394
298
WT
FSP1
FSP1 + M3814
BSCL2
BSCL2 + M3814
RANGAP1
RANGAP1 + M3814
PEX3
PEX3+ M3814
DHC1
DHC1 + i53/M3814
GLUT1
GLUT1 + i53/M3814
ASCL4 + i53/M3814
NUP93 + i53/M3814
SAC1
SAC1 + i53/M3814
POGZ + i53/M3814
SEC61B
SEC61B + i53/M3814

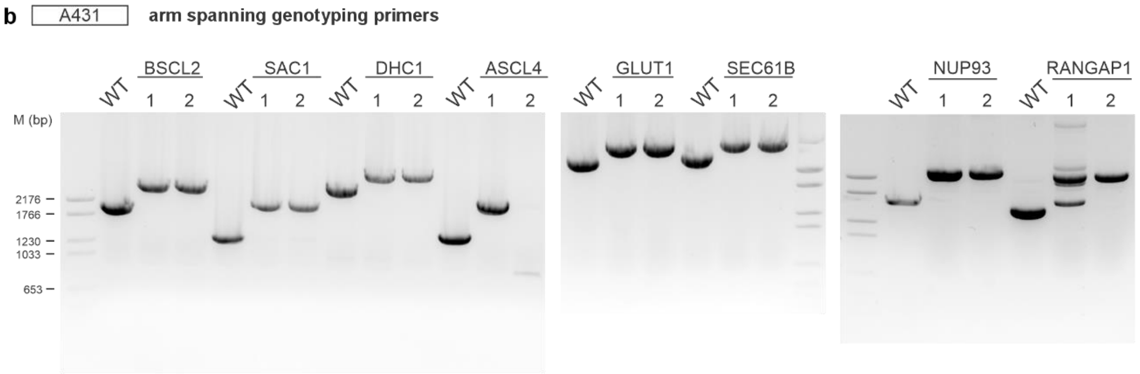
b
A431
arm spanning genotyping primers
M (bp)
2176
1766
1230
1033
653
WT
BSCL2
SAC1
DHC1
ASCL4
GLUT1
SEC61B
NUP93
RANGAP1

Data S1

c A549 short genotyping primers

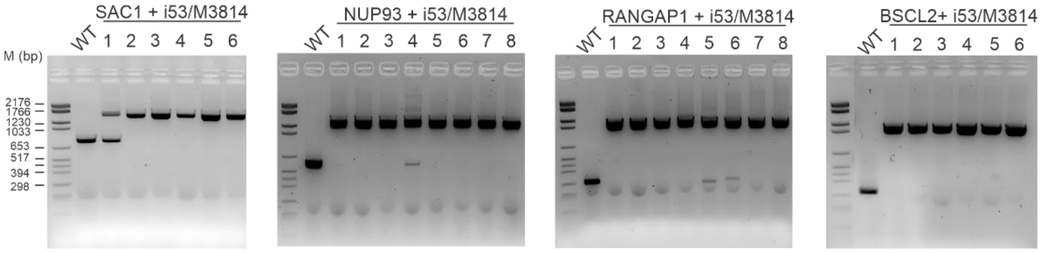

d A549 arm spanning genotyping primers

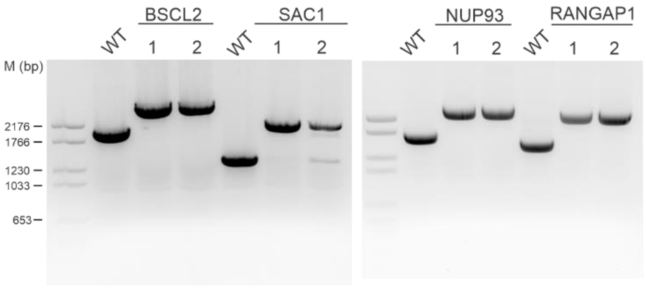

e HEK293A short genotyping primers

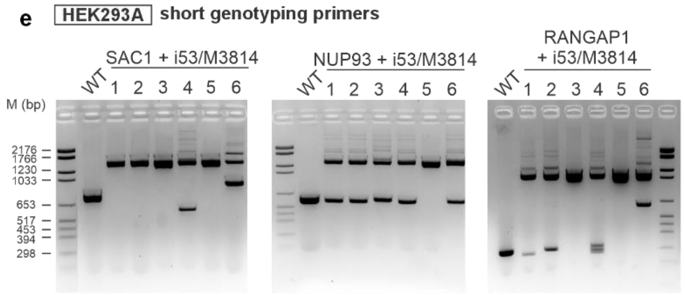

f HEK293A arm spanning genotyping primers

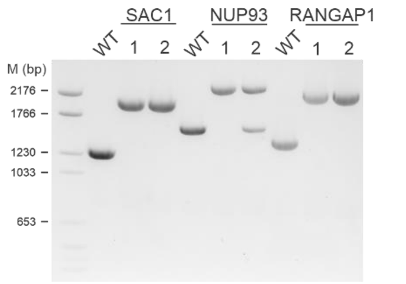

g U2OS short genotyping primers

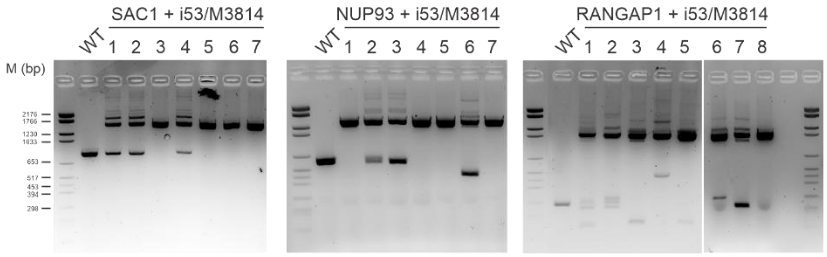

h U2OS arm spanning genotyping primers

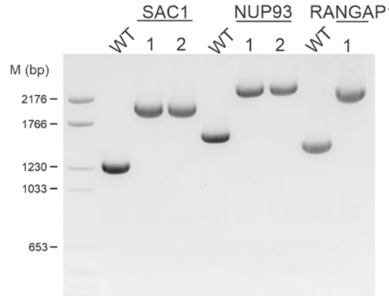

i hESCs short genotyping primers

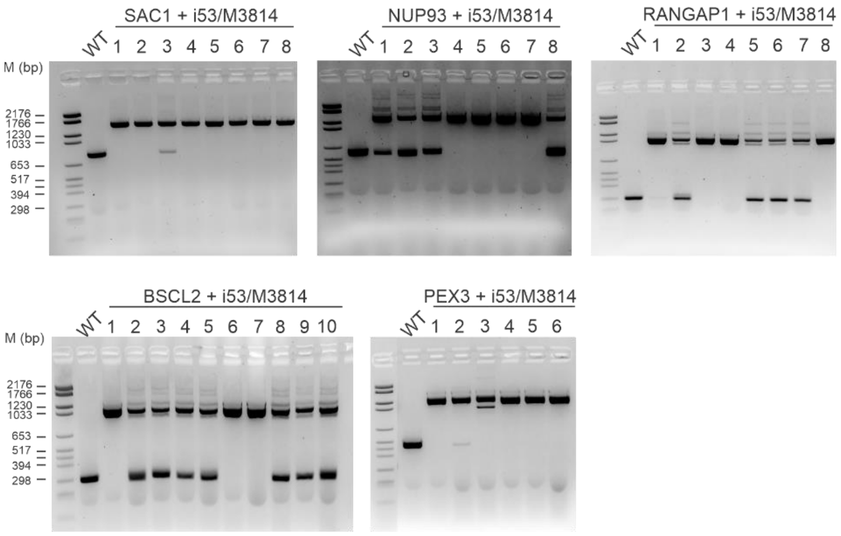

j hESCs arm spanning genotyping primers

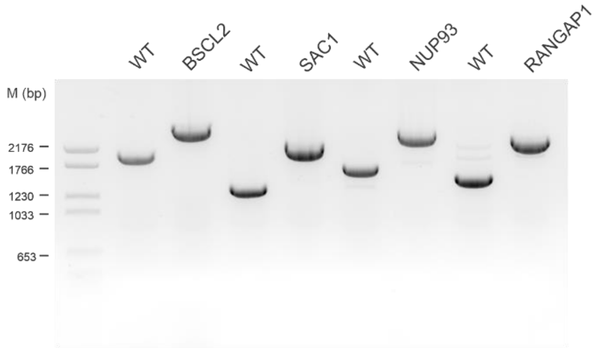

Data S1: Analysis of HiHo-AID2 cell lines using genomic PCR.

a-j, genotyping PCR using short genotyping primers (within homology arms of donor constructs) or arm-spanning primers of HiHo-AID2 A431 (**a-b**) A549 (**c-d**), HEK293A (**e-f**), U2OS (**g-h**), and hESC (**i-j**) cell lines. For arm-spanning primers, 1-2 homozygous clones identified with short primers for each cell line were used.
