# Supplementary material for: HiHo-AID2: boosting homozygous knock-in efficiency enables robust generation of human auxin-inducible degron cells
Source: Genome Biol. 2024 Feb 26;25:58. doi: 10.1186/s13059-024-03187-w (PMC10895734; doi:10.1186/s13059-024-03187-w)
Supplement: Supplementary file 3 — Additional file 3: Table S2-5. Table S2. Summary of sequences included. Table S3. sgRNA sequences. Table S4. PCR primers for genotyping PCR. Table S5. Arm-spanning primers for genotyping PCR. [file 13059_2024_3187_MOESM3_ESM.pdf]

Li et al. HiHo-AID2: boosting homozygous knock-in efficiency enables robust generation of human auxin-inducible degron cells

Additional file 3: Table S2–5.

Table S2: Summary of sequences included

Table S3: sgRNA sequences

Table S4: PCR primers for genotyping PCR

Table S5: Arm-spanning primers for genotyping PCR

Table S2: Summary of sequences included

**Auxin receptors**		7-DHC1.C- miniIAA7.3xFlag.P2A.BSD
P_{CAG}_*At*AFB2**(F74A)**-mCherry		8-Glut1.C- miniIAA7.3xFlag.P2A.BSD
*Os*TIR1**(F74G)**-mCherry		9-NUP93.C- miniIAA7.3xFlag.P2A.BSD
		10-SEC61B.N-BSD.P2A.miniIAA7.3xFlag
**CRISPR/Cas9**		11-POGZ.C- miniIAA7.3xFlag.P2A.BSD
P_{u6}_sgAAVS1.5/ P_{CAG}_Cas9-P2A-Puro		12-RABGGTA.N-miniIAA7.mEGFP
		13-LMNA.N-Sh_ble.P2A.miniIAA7.3xFlag
**HDR templates**		14-MYH9.N-Sh_ble.P2A.miniIAA7.3xFlag
1-SAC1.N-BSD.P2A.miniIAA7.3xFlag		15-LBR.C- miniIAA7.3xFlag.P2A.Sh_ble
2-RANGAP1.C-miniIAA7.3xFlag.P2A.BSD		16- LMNB1.N-miniIAA7.mEGFP
3-FSP1.C-miniIAA7.3xFlag.P2A.BSD		
4-ACSL4.C-miniIAA7.3xFlag.P2A.BSD		**HDR enhancers**
5-BSCL2.C-miniIAA7.3xFlag.P2A.BSD		EF1a_**i53**_HSV TK poly(A)
6-PEX3.C-miniIAA7.3xFlag.P2A.BSD		**P53DD** (mouse P53 Δ14-301aa.)

Auxin receptors:

P_{CAG}_*At*AFB2**(F74A)**-mCherry

(WT: **GCC** to TTC; F74G: **GCC** to GGC)

gacattgattattgactagttattaatagtaatcaattacggggtcattagttcatagcccatatatggagttccgcgttacataacttacggtaaatgg
cccgcctggctgaccgcccaacgacccccgcccattgacgtcaataatgacgtatgttcccatagtaacgccaatagggactttccattgacgtc
aatgggtggactatttacggtaaactgcccacttggcagtacatcaagtgtatcatatgccaagtacgccccctattgacgtcaatgacggtaaatg
gcccgcctggcattatgcccagtacatgaccttatgggactttcctacttggcagtacatctacgtattagtcatcgctattaccatgggtcgaggtg
agccccacgttctgcttcactctccccatctcccccccctccccacccccaattttgtatttatttattttttaattattttgtgcagcgatgggggcggg
ggggggggggcgcgcgccaggcggggcggggcggggcgaggggcggggcggggcgaggcggagaggtgcggcggcagccaatc
agagcggcgcgctccgaaagtttccttttatggcgaggcggcggcggcggcggccctataaaaagcgaagcgcgcggcgggcgggagtcg
ctgcgttgccttcgccccgtgccccgctccgcgccgcctcgcgccgcccgccccggctctgactgaccgcgttactcccacaggtgagcggg
cgggacggcccttctcctccgggctgtaattagcgcttggtttaatgacggctcgtttcttttctgtggctgcgtgaaagccttaaagggctccggg
agggccctttgtgcgggggggagcggctcgggggtgcgtgcgtgtgtgtgtgcgtggggagcgccgcgtgcggcccgcgctgcccggcg
gctgtgagcgctgcgggcgcggcgcggggctttgtgcgctccgcgtgtgcgcgaggggagcgcggccgggggcggtgccccgcggtgcg
gggggctgcgaggggaacaaaggctgcgtgcggggtgtgtgcgtgggggggtgagcagggggtgtgggcgcggcggtcgggctgtaa
cccccccctgcacccccctccccgagttgctgagcacggcccggcttcgggtgcggggctccgtgcggggcgtggcgcggggctcgccgt
gccgggcggggggtggcggcaggtgggggtgccgggcggggcggggccgcctcgggccggggagggctcgggggaggggcgcggc
ggccccggagcgccggcggctgtcgaggcgcggcgagccgcagccattgccttttatggtaatcgtgcgagagggcgcagggacttcctttg
tcccaaatctggcggagccgaaatctgggaggcgccgccgcacccctctagcgggcgcgggcgaagcggtgcggcgccggcaggaag
gaaatgggcggggagggccttcgtgcgtcgccgcgccgccgtccccttctccatctccagcctcggggctgccgcaggggggacggctgcctt
cgggggggacggggcagggcggggttcggcttctggcgtgtgaccggcggctctagagcctctgctaaccatgttcatgccttcttctttttcct
acag

ctcctgggcaacgtgctggttattgtgctgtctcatcattttggcaaaggctaccggactcagatctacc
atgaactactttcccgatgaggtcattgagcacgtctttgatttcgtcacaagccacaaggataggaacgccattagcctggtctgtaagagctggt
acaagatcgagcggtattccagacagaaggtgttcatcggcaactgctacgccatcaatccagagaggctgctgcggagatttccctgtctgaa
gagcctgaccctgaagggcaagccccac**GCC**gccgactttaacctggtgcctcacgagtggggaggattcgtgctgccatggatcgaggc
cctggcacggtccagagtgggcctggaggagctgaggctgaagcgcatggtggtgacagacgagtctctggagctgctgtctcggagcttcg
tgaacttcaagagcctggtgctggtgtcctgcgagggctttaccacagatggcctggcaagcatcgcagcaaactgtaggcacctgcgggacc
tggacctgcaggagaatgagatcgacgatcacagaggccagtggctgtcctgcttccccgatacctgtaccacactggtgacactgaactttgc
ctgcctggagggcgagacaaatctggtggccctggagaggctggtggcacgctctcccaacctgaagagcctgaagctgaatagggcagtg
cctctggacgcactggcaagactgatggcatgcgcaccacagatcgtggatctgggcgtgggctcctacgagaacgaccctgattccgagtctt
atctgaagctgatggccgtgatcaagaagtgtaccagcctgcgcagcctgtccggcttcctggaggcagcacctcactgcctgtccgcctttcac
ccaatctgtcacaacctgacatccctgaatctgtcttacgccgccgagatccacggctcccacctgatcaagctgatccagcactgcaagaagct
gcagaggctgtggatcctggactccatcggcgataagggcctggaggtggtggcctctacctgtaaggagctgcaggagctgcgcgtgttccc
atctgacctgctgggaggaggaaacaccgcagtgacagaggagggcctggtggcaatctctgccggatgccccaagctgcacagcatcctgt
atttttgtcagcagatgaccaacgccgccctggtgacagtggccaagaactgccccaatttcatcaggtttcgcctgtgcatcctggagcccaata
agcctgaccacgtgacatctcagcctctggatgagggcttcggcgccatcgtgaaggcctgcaagagcctgaggcgcctgtctctgagcggcc
tgctgaccgaccaggtgttcctgtacatcggcatgtatgccaaccagctggagatgctgtctatcgcctttgccggcgacacagataagggcatg
ctgtacgtgctgaatggctgtaagaagatgaagaagctggagatcagagacagcccttttggcgataccgccctgctggcagacgtgagcaag
tatgagacaatgcggagcctgtggatgagctcctgcgaggtgaccctgagcggctgtaagagactggccgagaaggccccatggctgaacgt
ggagatcatcaacgagaatgacaacaataggatggaggagaatggccacgagggccgccagaaggtggataagctgtatctgtataggacc
gtcgtcgggactcggatggatgcccccccctttgtctggattctgggcggatctggcggggtgagcaagggcgaggaggataacatggccatc
atcaaggagttcatgcgcttcaaggtgcacatggagggctccgtgaacggccacgagttcgagatcgagggcgagggcgagggccgcccct
acgagggcacccagaccgccaagctgaaggtgaccaagggtggccccctgcccttcgcctgggacatcctgtcccctcagttcatgtacggct
ccaaggcctacgtgaagcaccccgccgacatccccgactacttgaagctgtccttccccgagggcttcaagtgggagcgcgtgatgaacttcg
aggacggcggcgtggtgaccgtgacccaggactcctccctgcaggacggcgagttcatctacaaggtgaagctgcgcggcaccaacttcccc
tccgacggccccgtaatgcagaagaagaccatgggctgggaggcctcctccgagcggatgtaccccgaggacggcgccctgaagggcgag
atcaagcagaggctgaagctgaaggacggcggccactacgacgctgaggtcaagaccacctacaaggccaagaagcccgtgcagctgccc
ggcgcctacaacgtcaacatcaagttggacatcacctcccacaacgaggactacaccatcgtggaacagtacgaacgcgccgagggccgcc
actccaccggcggcatggacgagctgtacaagtagcggccgc

*Os*TIR1**(F74G)**-mCherry

(WT: **GGC** to TTC; F74A: **GGC** to GCC)

agatctacc
atgacctacttccctgaggaggtggtggagcacatcttctcctttctgccagcccagagggaccgcaacacagtgtctctggtgtgcaaagtgtg
gtacgagatcgagcgcctgtccaggagaggcgtgttcgtgggcaactgttatgccgtgcgggcaggaagagtggcagcaaggtttccaaatgt
gcgggccctgaccgtcaaggggaaaccccac**GGC**gccgactttaacctggtgccacctgattggggaggatatgcaggaccatggatcga
ggcagcagcaaggggatgccacggactggaggagctgaggatgaagcgcatggtggtgagcgacgagtccctggagctgctggcacgga
gcttcccacggtttagagccctggtgctgatctcttgcgagggctttagcaccgatggactggcagcagtggcaagccactgtaagctgctgcg
ggagctggacctgcaggagaacgaggtggaggatcggggccctagatggctgtcctgcttcccagactcttgtacaagcctggtgtccctgaa
ctttgcctgcatcaagggcgaagtgaatgccggcagcctggagagactggtgtccaggtctcctaacctgaggtccctgcgcctgaatcggag
cgtgtccgtggacaccctggccaagatcctgctgagaacaccaaacctggaggatctgggcaccggcaatctgacagacgatttccagaccg
agagctactttaagctgacatccgccctggagaagtgtaagatgctgaggtctctgagcggattctgggatgcatcccccgtgtgcctgtcttttat
ctaccctctgtgcgcccagctgaccggcctgaatctgtcttatgcccccaccctggacgcctctgatctgacaaagatgatcagcagatgcgtga
agctgcagaggctgtgggtgctggactgtatcagcgataagggcctgcaggtggtggccagctcctgcaaggacctgcaggagctgagagtg
ttccccagcgacttctacgtggccggctattccgccgtgacagaggagggactggtggccgtgtctctgggatgccctaagctgaacagcctgc
tgtatttctgtcaccagatgaccaatgccgccctggtgacagtggccaagaactgcccaaatttcaccaggtttcgcctgtgcatcctggagccag

gcaagcccgacgtggtgacatcccagcccctggatgagggctttggcgccatcgtgagagagtgcaagggcctgcagaggctgtccatctct
ggcctgctgaccgacaaggtgttcatgtacatcggcaagtatgccaagcagctggagatgctgtccatcgcctttgccggcgactctgataagg
gcatgatgcacgtgatgaacggctgtaagaatctgagaaagctggagatcagggactcccctttcggcgatgccgccctgctgggcaactttgc
ccgctacgagaccatgcggtctctgtggatgtctagctgcaatgtgacactgaagggctgtcaggtgctggcctctaagatgccaatgctgaacg
tggaagtgatcaacgagagggacggcagcaacgagatggaggagaatcacggcgatctgcccaaggtggagaagctgtacgtgtataggac
cacagcaggagcaagggacgatgcacctaacttcgtgaagatcctgggcggatctggcggggtgagcaagggcgaggaggataacatggc
catcatcaaggagttcatgcgcttcaaggtgcacatggagggctccgtgaacggccacgagttcgagatcgagggcgagggcgagggccgc
ccctacgagggcacccagaccgccaagctgaaggtgaccaagggtggccccctgcccttcgcctgggacatcctgtcccctcagttcatgtac
ggctccaaggcctacgtgaagcaccccgccgacatccccgactacttgaagctgtccttccccgagggcttcaagtgggagcgcgtgatgaac
ttcgaggacggcggcgtggtgaccgtgacccaggactcctccctgcaggacggcgagttcatctacaaggtgaagctgcgcggcaccaactt
cccctccgacggccccgtaatgcagaagaagaccatgggctgggaggcctcctccgagcggatgtaccccgaggacggcgccctgaaggg
cgagatcaagcagaggctgaagctgaaggacggcggccactacgacgctgaggtcaagaccacctacaaggccaagaagcccgtgcagct
gcccggcgcctacaacgtcaacatcaagttggacatcacctcccacaacgaggactacaccatcgtggaacagtacgaacgcgccgagggc
cgccactccaccggcggcatggacgagctgtacaagtagcggccgc

CRISPR/Cas9

P_{u6}_sgAAVS1.5/ P_{CAG}_Cas9-P2A-Puro

Gagggcctatttcccatgattccttcatatttgcatatacgatacaaggctgttagagagataattggaattaatttgactgtaaacacaaagatatta
gtacaaaatacgtgacgtagaaagtaataatttcttgggtagtttgcagttttaaaattatgttttaaaatggactatcatatgcttaccgtaacttgaaa
gtatttcgatttcttggctttatatatcttgtggaaaggacgaaacaccgctagtggccccactgtggggggttttagagctagaaatagcaagttaaa
ataaggctagtccgttatcaacttgaaaaagtggcaccgagtcggtgcttttttagatctcgagtctagaggaggtcgctgagtagtgcgcgagc
aaaatttaagctacaacaaggcaaggcttgaccgacaattgcatgaagaatctgcttagggttaggcgttttgcgctgcttcgcgatgtacgggcc
agatatacgcgttgacattgattattgactagttattaatagtaatcaattacggggtcattagttcatagcccatatatggagttccgcgttacataact
tacggtaaatggcccgcctggctgaccgcccaacgacccccgcccattgacgtcaataatgacgtatgttcccatagtaacgccaatagggact
ttccattgacgtcaatgggtggactatttacggtaaactgcccacttggcagtacatcaagtgtatcatatgccaagtacgccccctattgacgtcaa
tgacggtaaatggcccgcctggcattatgcccagtacatgaccttatgggactttcctacttggcagtacatctacgtattagtcatcgctattaccat
gggtcgaggtgagccccacgttctgcttcactctccccatctcccccccctccccacccccaattttgtatttatttattttttaattattttgtgcagcga
tgggggcggggggggggggggcgcgcgccaggcggggcggggcggggcgaggggcggggcggggcgaggcggagaggtgcggc
ggcagccaatcagagcggcgcgctccgaaagtttccttttatggcgaggcggcggcggcggcggccctataaaaagcgaagcgcgcggcg
ggcgggagtcgctgcgttgccttcgccccgtgccccgctccgcgccgcctcgcgccgcccgccccggctctgactgaccgcgttactcccac
aggtgagcgggcgggacggcccttctcctccgggctgtaattagcgcttggtttaatgacggctcgtttcttttctgtggctgcgtgaaagccttaa
agggctccgggagggccctttgtgcgggggggagcggctcgggggggtgcgtgcgtgtgtgtgtgcgtggggagcgccgcgtgcggcccg
cgctgcccggcggctgtgagcgctgcgggcgcggcgcggggctttgtgcgctccgcgtgtgcgcgaggggagcgcggccgggggcggtg
ccccgcggtgcgggggggctgcgaggggaacaaaggctgcgtgcggggtgtgtgcgtgggggggtgagcagggggtgtgggcgcggcg
gtcgggctgtaacccccccctgcacccccctccccgagttgctgagcacggcccggcttcgggtgcggggctccgtgcggggcgtggcgcg
gggctcgccgtgccgggcggggggtggcggcaggtgggggtgccgggcggggcggggccgcctcgggccggggagggctcgggga
ggggcgcggcggccccggagcgccggcggctgtcgaggcgcggcgagccgcagccattgccttttatggtaatcgtgcgagagggcgca
gggacttcctttgtcccaaatctggcggagccgaaatctgggaggcgccgccgcaccccctctagcgggcgcgggcgaagcggtgcggcgc
cggcaggaaggaaatgggcggggagggccttcgtgcgtcgccgcgccgccgtccccttctccatctccagcctcggggctgccgcagggg
gacggctgccttcgggggggacggggcagggcggggttcggcttctggcgtgtgaccggcggctctagagcctctgctaaccatgttcatgc
cttcttctttttcctacagctcctgggcaacgtgctggttattgtgctgtctcatcattttggcaaagaattgctaccggactcagatccaccggtcgcc
accatggactataaggaccacgacggagactacaaggatcatgatattgattacaaagacgatgacgataagatggccccaaagaagaagcg
gaaggtcggtatccacggagtcccagcagccgacaagaagtacagcatcggcctggacatcggcaccaactctgtgggctgggccgtgatca
ccgacgagtacaaggtgcccagcaagaaattcaaggtgctgggcaacaccgaccggcacagcatcaagaagaacctgatcggagccctgct
gttcgacagcggcgaaacagccgaggccacccggctgaagagaaccgccagaagaagatacaccagacggaagaaccggatctgctatct

gcaagagatcttcagcaacgagatggccaaggtggacgacagcttcttccacagactggaagagtccttcctggtggaagaggataagaagc
acgagcggcaccccatcttcggcaacatcgtggacgaggtggcctaccacgagaagtaccccaccatctaccacctgagaaagaaactggtg
gacagcaccgacaaggccgacctgcggctgatctatctggccctggcccacatgatcaagttccggggccacttcctgatcgagggcgacctg
aaccccgacaacagcgacgtggacaagctgttcatccagctggtgcagacctacaaccagctgttcgaggaaaaccccatcaacgccagcgg
cgtggacgccaaggccatcctgtctgccagactgagcaagagcagacggctggaaaatctgatcgcccagctgcccggcgagaagaagaat
ggcctgttcggaaacctgattgccctgagcctgggcctgacccccaacttcaagagcaacttcgacctggccgaggatgccaaactgcagctg
agcaaggacacctacgacgacgacctggacaacctgctggcccagatcggcgaccagtacgccgacctgtttctggccgccaagaacctgtc
cgacgccatcctgctgagcgacatcctgagagtgaacaccgagatcaccaaggcccccctgagcgcctctatgatcaagagatacgacgagc
accaccaggacctgaccctgctgaaagctctcgtgcggcagcagctgcctgagaagtacaaagagattttcttcgaccagagcaagaacggct
acgccggctacattgacggcggagccagccaggaagagttctacaagttcatcaagcccatcctggaaaagatggacggcaccgaggaact
gctcgtgaagctgaacagagaggacctgctgcggaagcagcggaccttcgacaacggcagcatccccccaccagatccacctgggagagctg
cacgccattctgcggcggcaggaagatttttacccattcctgaaggacaaccgggaaaagatcgagaagatcctgaccttccgcatcccctact
acgtgggccctctggccaggggaaacagcagattcgcctggatgaccagaaagagcgaggaaaccatcaccccctggaacttcgaggaagt
ggtggacaagggcgcttccgcccagagcttcatcgagcggatgaccaacttcgataagaacctgcccaacgagaaggtgctgcccaagcaca
gcctgctgtacgagtacttcaccgtgtataacgagctgaccaaagtgaaatacgtgaccgagggaatgagaaagcccgccttcctgagcggcg
agcagaaaaaggccatcgtggacctgctgttcaagaccaaccggaaagtgaccgtgaagcagctgaaagaggactacttcaagaaaatcgag
tgcttcgactccgtggaaatctccggcgtggaagatcggttcaacgcctccctgggcacataccacgatctgctgaaaattatcaaggacaagg
acttcctggacaatgaggaaaacgaggacattctggaagatatcgtgctgaccctgacactgtttgaggacagagagatgatcgaggaacggct
gaaaacctatgcccacctgttcgacgacaaagtgatgaagcagctgaagcggcggagatacaccggctggggcaggctgagccggaagctg
atcaacggcatccgggacaagcagtccggcaagacaatcctggatttcctgaagtccgacggcttcgccaacagaaacttcatgcagctgatcc
acgacgacagcctgacctttaaagaggacatccagaaagcccaggtgtccggccagggcgatagcctgcacgagcacattgccaatctggcc
ggcagccccgccattaagaagggcatcctgcagacagtgaaggtggtggacgagctcgtgaaagtgatgggccggcacaagcccgagaac
atcgtgatcgaaatggccagagagaaccagaccacccagaagggacagaagaacagccgcgagagaatgaagcggatcgaagagggcat
caaagagctgggcagccagatcctgaaagaacaccccgtggaaaacacccagctgcagaacgagaagctgtacctgtactacctgcagaatg
ggcgggatatgtacgtggaccaggaactggacatcaaccggctgtccgactacgatgtggaccatatcgtgcctcagagctttctgaaggacg
actccatcgacaacaaggtgctgaccagaagcgacaagaaccggggcaagagcgacaacgtgccctccgaagaggtcgtgaagaagatga
agaactactggcggcagctgctgaacgccaagctgattacccagagaaagttcgacaatctgaccaaggccgagagaggcggcctgagcga
actggataaggccggcttcatcaagagacagctggtggaaacccggcagatcacaaagcacgtggcacagatcctggactcccggatgaaca
ctaagtacgacgagaatgacaagctgatccgggaagtgaaagtgatcaccctgaagtccaagctggtgtccgatttccggaaggatttccagttt
tacaaagtgcgcgagatcaacaactaccaccacgcccacgacgcctacctgaacgccgtcgtgggaaccgccctgatcaaaaagtaccctaa
gctggaaagcgagttcgtgtacggcgactacaaggtgtacgacgtgcggaagatgatcgccaagagcgagcaggaaatcggcaaggctacc
gccaagtacttcttctacagcaacatcatgaactttttcaagaccgagattaccctggccaacggcgagatccggaagcggcctctgatcgagac
aaacggcgaaaccggggagatcgtgtgggataagggccgggattttgccaccgtgcggaaagtgctgagcatgccccaagtgaatatcgtga
aaaagaccgaggtgcagacaggcggcttcagcaaagagtctatcctgcccaagaggaacagcgataagctgatcgccagaaagaaggactg
ggaccctaagaagtacggcggcttcgacagccccaccgtggcctattctgtgctggtggtggccaaagtggaaaagggcaagtccaagaaac
tgaagagtgtgaaagagctgctggggatcaccatcatggaaagaagcagcttcgagaagaatcccatcgactttctggaagccaagggctaca
aagaagtgaaaaaggacctgatcatcaagctgcctaagtactccctgttcgagctggaaaacggccggaagagaatgctggcctctgccggcg
aactgcagaagggaaacgaactggccctgccctccaaatatgtgaacttcctgtacctggccagccactatgagaagctgaagggctcccccg
aggataatgagcagaaacagctgtttgtggaacagcacaagcactacctggacgagatcatcgagcagatcagcgagttctccaagagagtga
tcctggccgacgctaatctggacaaagtgctgtccgcctacaacaagcaccgggataagcccatcagagagcaggccgagaatatcatccac
ctgtttaccctgaccaatctgggagcccctgccgccttcaagtactttgacaccaccatcgaccggaagaggtacaccagcaccaaagaggtgc
tggacgccaccctgatccaccagagcatcaccggcctgtacgagacacggatcgacctgtctcagctgggaggcgacaaaaggccggcggc
cacgaaaaaggccggccaggcaaaaaagaaaaaggaattcggaagcggcgcaacaaacttctcactactcaaacaagcaggtgacgtgga
ggagaatcccgggcctcttaagatgaccgagtacaagcccacggtgcgcctcgccacccgcgacgacgtccccagggccgtacgcaccctc
gccgccgcgttcgccgactaccccgccacgcgccacaccgtcgatccggaccgccacatcgagcgggtcaccgagctgcaagaactcttcct
cacgcgcgtcgggctcgacatcggcaaggtgtgggtcgcggacgacggcgccgcggtggcggtctggaccacgccggagagcgtcgaag
cgggggcggtgttcgccgagatcggcccgcgcatggccgagttgagcggttcccggctggccgcgcagcaacagatggaaggcctcctgg

cgccgcaccggcccaaggagcccgcgtggttcctggccaccgtcggcgtctcgcccgaccaccagggcaagggtctgggcagcgccgtcg
tgctccccggagtggaggcggccgagcgcgccggggtgcccgccttcctggagacctccgcgccccgcaacctccccttctacgagcggct
cggcttcaccgtcaccgccgacgtcgaggtgcccgaaggaccgcgcacctggtgcatgacccgcaagcccggtgcctgaatcgatgcggga
ctctggggttcgaaatgaccgaccaagcgacgcccaacctgccatcacgagatttcgattccaccgccgccttctatgaaaggttgggcttcgg
aatcgttttccgggacgccggctggatgatcctccagcgcggggatctcatgctggagttcttcgcccaccctaggggaggctaactgaaaca
cggaaggagacaataccggaaggaacccgcgctatgacggcaataaaaagacagaataaaacgcacggtgttgggtcgtttgttcataaacg
cggggttcggtcccagggctggcactctgtcgataccccaccgagaccccattggggccaatacgcccgcgtttcttccttttccccaccccacc
ccccaagttcgggtgaaggcccagggctcgcagccaacgtcggggcggcaggccctgccatagcctc

HDR templates:

1- SAC1.N-BSD.P2A.miniIAA7.3xFlag

LA (left arm)-BSD.P2A.miniIAA7.3xFlag.linker-RA (right arm)

CTTTCCAAGGTCTTGTAATACTTTCACATTGTGAATATACATCTTTACAAGACCCAGTAGT
ACATGGGTCAAGTGTGGCAAAACAGACATCGCGAGCCAGCCAGGAGTCGAACCTAGAA
TCTTCTGATCCGTAGTCAGACGCGTTATCCATTGCGCCACTGGCCCCGACTTGCTAAAGC
CTTCTCCGTGATAGCTGATCACCCTTATACAGTTTGAACGGGCTTCCGGGTACCAACGAT
CCATTTTGCCCTTGCGCCGCGCTCCCGCTGCTCTGCCTTTCCCCAAAACGCGCCCCACCA
ATCCGCAGCGGCTTCGTCACGGCGCTCTTCGTCGCACTCCAACCCGCCGCGTCGGCGGC
ATCTCTCGCGTGCTCTTGCTTGCGCACTAATTTGTCCTCCACCGCCTCCCGTTCAGGCCG
GCTCTCCTGCGGGGCGGGGAAAGGGGTGGGAAGAGGCTGGGAGCTGCGGCCGCTGTG
CCAGGGTGACCGGTAGAGTTGTAGCCGAGGTGGCGGCGCGGGGCGGGGCGGGCGGAG
AGAGAAGGAAGGAGGTGGTTGTGCAGGATGGGAGCCAAGCCACTGAGCCAGGAGGAG
AGCACACTGATTGAAAGAGCAACTGCCACTATTAACAGCATTCCCATTAGCGAGGACTA
TTCTGTGGCAAGCGCCGCCCTGAGCTCCGACGGAAGGATCTTCACCGGCGTGAACGTG
TACCACTTTACAGGCGGCCCCTGCGCCGAGCTGGTGGTGCTGGGAACCGCAGCAGCAG
CAGCAGCAGGCAATCTGACATGTATCGTGGCCATCGGCAACGAGAATAGGGGCATCCTG
TCCCCATGCGGCCGGTGTAGACAGGTGCTGCTGGATCTGCACCCTGGCATCAAGGCCAT
CGTGAAGGACTCTGATGGACAGCCAACCGCCGTCGGGATTAGAGAACTGCTGCCATCA
GGATACGTCTGGGAGGGCGGCTCCGGCGCAACCAACTTTTCTCTGCTGAAGCAGGCCG
GCGACGTGGAAGAAAATCCAGGCCCCggcttctctgagaccgtggacctgatgctgaacctgcagtccaataaggagg
gctctgtggatctgaagaacgtgagcgccgtgcctaaggagaagaccacactgaaggacccatccaagccccctgccaaggcacaggtggt
gggatggccacccgtgcggaactacagaaagaatatgatgacccagcagaagacaagctccGactacaaagaccatgacggtgattataaa
gatcatgacatcgattacaaggatgacgatgacaagtccggaGGcGGAGGGAGCGGGGGAGGCGGATCTGGCG
GAGGCGGaTCCGCGACGGCGGCCTACGAGCAGCTGAAGCTGTGAGTCCCACGGGCCAG
AGGCCTGAGGCGCGGCGGGCGGGGCGGCAGGTGCGGGCCCTGGCCTCGGGGAGGGCT
TCTGAGTCCCGGATTGCAGATAGGGTGTGGGAGCTCCTCGCATTTACCGGTTTTCCTCAG
CCGGCGCGGCCTCTCCTAGGCCTCGCCCGAGTAGCCATAGGCCGGGAACCAGCGGCTCG
CCGGCCTTTCTCCACCGTGCTCGGCCCCAGCGCCCACGGCTGAAGAGAGAAGCTGCCG
ACTGGCCCCCACCGAGCCGGGGTCGGCGTTATGATGCGGCCCTTCAGGGCACGCTTGTC
CCCACTTTCTATGCAGAGCTACCGACTAAATTTATCTGTATGACTTAACAGAGAAAAAGA
GTTTTCTCTTACTTAACGGACCTACAGCAGCAATTTAGAGTCTACGCACAAATCAACAAA
TCTTTGTTTTGCTTACAGCCTATCTGGGTACCGAAGCCTGCCTGCCCCCTCTTCAGTGTG
CAGAAAAAATTTAAAGTACAGTCAGTAGTCTTAAGGTCCTGAAGGAGTTTACTTTGGTT
TAAATTTTAAACAGCTCCAGCCTAAAGCAGGTTCTTAAGTCAGTGTCGACAGCCAG

2- RANGAP1.C-miniIAA7.3xFlag.P2A.BSD

LA (left arm)-linker.miniIAA7.3xFlag.P2A.BSD-RA (right arm)

GCCAGTCCTATGTGGCCACTGGCAGCCAGTAAGGATTCCCTGGCCTATGTCTGCTGGAA
GGGCCCTTGGAGAGCCTGGCTGAGGGGGCCTTCATTCTGGCCAGCTCTGGGCTGTGTGG
CGTTGGGTGCCTGGCTCTCTGAGCCTCTCGCACCTCCCATCCCCACCCACGGTGTGCAG
AAGGCAGAGGGCTAATGTCTCTCTTGCTGAGTCGTTGTGCAAATTAACAAAAATGAGTC
TGGCACCAAAATGGCCCCCACACTTGGAAAACAGGCCAAGAGGCATCCACCGACCTGC
TGACCCCTCTTTCCCCGCAGGCCCAACAGCGCCCTGGAATCCTGCTCCTTCGCCCGCCA
CAGTCTGCTGCAGACGCTGTACAAaGTCGGATCtGGCGGAGGAGGGAGCGGGGGAGGC
GGATCTGGCGGAGGCGGaTCCGGCTTCTCTGAGACCGTGGACCTGATGCTGAACCTGCA
GTCCAATAAGGAGGGCTCTGTGGATCTGAAGAACGTGAGCGCCGTGCCTAAGGAGAAG
ACCACACTGAAGGACCCATCCAAGCCCCCTGCCAAGGCACAGGTGGTGGGATGGCCAC
CCGTGCGGAACTACAGAAAGAATATGATGACCCAGCAGAAGACAAGCTCCGACTACAA
AGACCATGACGGTGATTATAAAGATCATGACATCGATTACAAGGATGACGATGACAAGG
GCTCCGGCGCCACCAACTTTTCTCTGCTGAAGCAGGCCGGCGATGTGGAGGAGAATCCT
GGCCCAGGAGCCAAGCCACTGAGCCAGGAGGAGAGCACACTGATTGAAAGAGCAACT
GCCACTATTAACAGCATTCCCATTAGCGAGGACTATTCTGTGGCAAGCGCCGCCCTGAGC
TCCGACGGAAGGATCTTCACCGGCGTGAACGTGTACCACTTTACAGGCGGCCCCTGCGC
CGAGCTGGTGGTGCTGGGAACCGCAGCAGCAGCAGCAGCAGGCAATCTGACATGTATC
GTGGCCATCGGCAACGAGAATAGGGGCATCCTGTCCCCATGCGGCCGGTGTAGACAGGT
GCTGCTGGATCTGCACCCTGGCATCAAGGCCATCGTGAAGGACTCTGATGGACAGCCAA
CCGCCGTCGGGATTAGAGAACTGCTGCCATCAGGATACGTCTGGGAGGGCTAGTGTACA
AGTAGACTCAAAGCCTCTCCCATCCCTTGGCCTGGACCAGTGAGCTGGGGAGGGA
CTCGGATGAACTGAGGCGCAGCCTACGCCATTGCCTTGGACAGGACTCTGGCCACAGG
CAGGGCGGGTCTGTGTCCCATGTGTCCTGTCAGTCCCCTGAGTATGTGTGTGGGTGTGG
CGCATGTGCAGGTCTGTGCCTCCTGTCGGGATTTGGGTTTTAACGTCTTCTGCTGGCCC
AGCCCTGCTCTGTTGTGGGGAGTTGGCCCCCAGGGGAAAGGGCTGTGAGCTGCTCCGC
CATTAAACTCACCTCCACCTGAGGGCGCTCTGCTGATCTCCGCCTGGGCCCTGATGGCC
GTCCCCACCCACCTGCCTTCCGGCCCGGCTCCCTGGCGGAGCCAGAACCCAGGGAGTT
GCCCGCGTGCTGTCCTTCCCCTCTGTGTTGTGATTGGGTTGTTTCCTGCCCTGCCTGGGG
CTGCTTCTCGTCACCAAGCCCTGGTCCTGCGGCAGCTGTCACCCCTACCATCCATACCA
CTGTGCTGACCGCTCAGCCTGAAGAGCAGAGAATGCCATGGGTGGGACTGTGGGGGTC
GGATCGTGGGGTTGTTGGCAGAGGGCAACCCTGGGCCCCACACCGTGTGGACAGGCAG
ACACCAGATTGTCCAGGAGCAGGAGCTGCTGGGACTGCGCTGGCCCCGGACCTAGTGG
GCCTTCTCCTGGCTGCTGAGATGTCGTCTGTGACTGGCCTGGCTGGAGGGGGAGTGTTG
ACAACCCAAAGCTGTTCTCCAGTCTGGGGAGGGAGAGGCAGGGTCCCCAATGTCCGAG
CTGCATCTGGACGCTGCTCTTAAAGGACCTCCTGGGGCAGGGGAGCGGTAGGGTCTGG
ACTGGGCAGATGCTGTATGACCTCCCTGAGCACCCGTGACTGCCCCATGCTTTCCCCTT
TGTGCTCTGTGTGTGTCTGGGCTGTGCCCGGGGGCTTCACAAATAAAGTCGTGTGGCAG
CTTCAGAGACT

3- FSP1.C-miniIAA7.3xFlag.P2A.BSD

LA (left arm)-linker.miniIAA7.3xFlag.P2A.BSD-RA (right arm)

GAGAGGCCCCATGTGCTTTGCAGTTTTAATAGAGTTTGGGGTTAAACATGGGGAAGCCT
TTTTGGGCTATCCCATACCTGGCCACTAGAACAAACTCTCCAAGCTGGTGATGGAAACAT
CCCCTTAGAGCTCTTTAAAAATAGGCTGGGCGCGGTGGCTCACACCTGTAACCCCAGCA
CTTTGGGAGGCCGAGGCGGGCAGATCACCTGAGGTCGGGAGTTCGAGACCAGCCTGAC
CAACATGGAGAAACCCCGTCTCTACTAAAAATACAAAATTAGCCAGGTGTGGTGGTGCA
TGCCTGTAATCCCAGCTACTCGGGAGGTTGAGGCAGGAGAATGGCTTGAACCCAGGAG
GTGGAGGTTGCAGCGAGCCAAGATCACACCATTGCACTCCAGCCTGGGCAACAAGAGC
AAAACTCTGTCTCAAAAAAACAAACAAAAAAAAATCAGGGTCTCACCCACCTTCCTAA
AGCGATTTCACATTTCCGGCCCTGAGTCTGAAGGCCTCGGGAAGGATCGGCACTCTGGG
AGGTGCAGCCCCAGGCGGTTCAGTGAGGTTCTGGTTGTTTCTGTGTCTCTGCTTGCAGG
TGCACTGACGTTCCTCCTGTCCATGGGGAGAAATGACGGTGTGGGCCAAATCAGTGGCT
TCTATGTGGGCCGGCTCATGGTTCGGCTGACCAAGAGCCGGGACCTGTTCGTCTCTACG
AGCTGGAAAACCATGAGGCAGTCTCCACCTGGATCtGGCGGAGGAGGGAGCGGGGGAG
GCGGATCTGGCGGAGGCGGaTCCGGCTTCTCTGAGACCGTGGACCTGATGCTGAACCTG
CAGTCCAATAAGGAGGGCTCTGTGGATCTGAAGAACGTGAGCGCCGTGCCTAAGGAGA
AGACCACACTGAAGGACCCATCCAAGCCCCCTGCCAAGGCACAGGTGGTGGGATGGCC
ACCCGTGCGGAACTACAGAAAGAATATGATGACCCAGCAGAAGACAAGCTCCGACTAC
AAAGACCATGACGGTGATTATAAAGATCATGACATCGATTACAAGGATGACGATGACAA
GGGCTCCGGCGCCACCAACTTTTCTCTGCTGAAGCAGGCCGGCGATGTGGAGGAGAAT
CCTGGCCCAGGAGCCAAGCCACTGAGCCAGGAGGAGAGCACACTGATTGAAAGAGCA
ACTGCCACTATTAACAGCATTCCCATTAGCGAGGACTATTCTGTGGCAAGCGCCGCCCTG
AGCTCCGACGGAAGGATCTTCACCGGCGTGAACGTGTACCACTTTACAGGCGGCCCCTG
CGCCGAGCTGGTGGTGCTGGGAACCGCAGCAGCAGCAGCAGCAGGCAATCTGACATGT
ATCGTGGCCATCGGCAACGAGAATAGGGGCATCCTGTCCCCATGCGGCCGGTGTAGACA
GGTGCTGCTGGATCTGCACCCTGGCATCAAGGCCATCGTGAAGGACTCTGATGGACAGC
CAACCGCCGTCGGGATTAGAGAACTGCTGCCATCAGGATACGTCTGGGAGGGCTAGTGT
ACAAGTGATGGAGAGGCCAGGCGGGAGAACTACCGCAGCAGGTGGGCGTACGGACTG
CTTGGCGCATGGCACCCGCCTGGCAAGTGCTAGAACTAATGCTATTCTTCTGGAATAAGA
TGCCAATGATGTGGTGGCTAGAAATGCAACTTGTATAAAACAAAAATGGGAGAGAGAGA
GGTATTAAACAAATACCCCCCTTAGAGGATACTTTCTGGGTTTGGAAGGTGTGCTTGCTG
TGGTACTGGGTGAGCGGCTCATGTGTGCTGGCTGCATGGTGCTGGGGAGGCCACAGCCA
GCCCTTCCTCTGCACCTGCCTCCTCTGGGATGTGCATGTGTGTGTACGTGCTTGTGGTCA
TGACGCGTGCCATTTAGAGCTCTCAGAGCAGGGCAGATTGCTGGGCTCTGGTGGCCAGT
GTCTGTCTGTGAGGGCAGGAAGGAGAGCTGCACATTGAGAACAAAGGAGGGACCTGA
GGTGGAGAGAGGCCCAGCACCCCAAATCTCTGCCATCACACGGTCGGGGAGCCCATAC
ATTCTGCAACAACCAGGGACTTCACAGGAGCCTTGTTTTCAATTTGCTAACAGGTGCAT
AATCCC

4- ACSL4.C- miniIAA7.3xFlag.P2A.BSD

LA (left arm)-linker.miniIAA7.3xFlag.P2A.BSD-RA (right arm)

GCACAGTATTTGGGTGGGAGTGACCCGATTTTCCAGGTGACCCGTTTCTTTGACTAGGA
AAGGGAACTCCCTGACCCCTTGTGCTTCCCGAGTGAGGCAATGCCTCGCCCTGCTTCGG
CTCGCGCATGGTGCGCACACCCACTGACCTGCGCCCACTGTCTGGCACTCCCTAGTGAG

ATGAACCTGGTACCTCAGATGGAAATGCAGAAATCACCCGTCTTCTGTGTCGCTCAGGC
TGGGAGCTGTAGACCAGAGCTGTTCGTATTTGGCCATCTTGGCTCCTCCCCCTTCTCTTT
TGTTATGAGTGGTCAGGGATGTCCTCTCCGGGGTGGTGGTATTTGAGCAGACATATGGAT
AAAGTATGGAAGTGACACACAACTTTATTGAGAAAGACCATCTCAGGCAAATATAAAGT
CTCTGAAGAGGAAGATGTGTGTGTATGTGTGTGTGTGTGAGTATAAATAATGTGTATATGT
ATGTATATTTTAATAACCACATAGACTAAAACTATCCAACTGAAACAACTTAGGTATACTT
TGAGAAATATAAAATGAAATTGTAAGGTAGAGGTAGCATTAATAAACTATCTAGTATACCA
TTAAATAAGTTTGTGGTGATTGGTGATTCAACACCCGAATCATCTAGGTAAATCCTGAAA
AACTCATATTTTATTAATTGCTTCTCCTTCCCCTTCTTTCCCCGTCCCCACTCCTTTCTCGT
CTCTTTCCCCCCCTCTCCTTTTCTCCCATCTTTCACTTCTAATTTCAGTGAAATTGGAGCG
ATTTGAAATTCCAATCAAGGTTCGATTAAGCCCAGAGCCATGGACCCCTGAAACTGGTTT
GGTAACTGATGCTTTCAAACTGAAAAGGAAGGAGCTGAGGAACCATTACCTCAAAGAC
ATTGAACGAATGTATGGcGGCAAAGGATCtGGCGGAGGAGGGAGCGGGGGAGGCGGATC
TGGCGGAGGCGGaTCCGGCTTCTCTGAGACCGTGGACCTGATGCTGAACCTGCAGTCCA
ATAAGGAGGGCTCTGTGGATCTGAAGAACGTGAGCGCCGTGCCTAAGGAGAAGACCAC
ACTGAAGGACCCATCCAAGCCCCCTGCCAAGGCACAGGTGGTGGGATGGCCACCCGTG
CGGAACTACAGAAAGAATATGATGACCCAGCAGAAGACAAGCTCCGACTACAAAGACC
ATGACGGTGATTATAAAGATCATGACATCGATTACAAGGATGACGATGACAAGGGCTCCG
GCGCCACCAACTTTTCTCTGCTGAAGCAGGCCGGCGATGTGGAGGAGAATCCTGGCCC
AGGAGCCAAGCCACTGAGCCAGGAGGAGAGCACACTGATTGAAAGAGCAACTGCCAC
TATTAACAGCATTCCCATTAGCGAGGACTATTCTGTGGCAAGCGCCGCCCTGAGCTCCGA
CGGAAGGATCTTCACCGGCGTGAACGTGTACCACTTTACAGGCGGCCCCTGCGCCGAGC
TGGTGGTGCTGGGAACCGCAGCAGCAGCAGCAGCAGGCAATCTGACATGTATCGTGGC
CATCGGCAACGAGAATAGGGGCATCCTGTCCCCATGCGGCCGGTGTAGACAGGTGCTGC
TGGATCTGCACCCTGGCATCAAGGCCATCGTGAAGGACTCTGATGGACAGCCAACCGCC
GTCGGGATTAGAGAACTGCTGCCATCAGGATACGTCTGGGAGGGCTAGTGTACAAGTAA
AATGTTGTTGTCTTATTGACAGTTGTGCAGGAGGTAGCCTGGTGGTTTTCAACCTCTAGA
ATTTTAAGCCTTTGTTGAACTGTTAGAATGTAAGGTATATCATTCTAAAGATAGAGTAAAA
AGAAAACAAAACCAAAAGTTATTAAAATTGTTGTCCGGTTTACTTTAACTTAGTTTTGCA
TAGTTCTAGTGCAGCTGAAATTGAAAAGTTATTTCCCTTTAGCTGTGTTATTATAGAGCAG
AAATTCTGTTTTTAAAAATTAGCCTAAGATATACTTGTTTTTGTAAAGAAAAATATTTAAT
GTTGAACAAAATAAATTGGAGTTGGAGTAGAATGTAGTTTGAGGAAATTTGCAGCTTCC
AATGCCTCTTGTCTTCCTATTTCAGAAGTTTAAATATTAAGCATGACAGAAAATATGTATT
AACACTACTCAAAGC

5- BSCL2.C- miniIAA7.3xFlag.P2A.BSD

LA (left arm)-linker.miniIAA7.3xFlag.P2A.BSD-RA (right arm)

GGAAGGAAGTCCAACGAAGGATCTCTGCTCATCAGCCAGGTAAAGGTGTTGGCTGGGG
AGCTGTTGAGGTGGGCTAGGTTGAGCTGGGCTGGGCCAGAAGGAGTCCCTGCTTCATCC
AGTATGAGGCTTAGGGTATCACCGACTGAGACAAGGGTCAAAGGAAAGGCGAGGAAGG
TGAGGTTGGCACTGCCGTCCAGGCACAGCCACCCTAACCCTAACCTGTCCTCTCCCTCT
TTGGAGGTGCAGGGCCTGAAGGCCAGGAGGAGTCAACTCCGCAATCAGATGTTACAGA
GGATGGTGAGAGCCCTGAAGATCCCTCAGGGACAGGTATGGCGCAGCCACCACACTCT
CTCCTTCCTGACTCCTTGGCTTTCTCCTTCAGTGTCTGGGTCTGACCCCTGCATTCCCCTT
TTGGCTGCAGAGGGTCAGCTGTCCGAGGAGGAGAAACCAGATCAGCAGCCCCTGAGCG

GAGAAGAGGAGCTAGAGCCTGAGGCCAGTGATGGTGAGGGGCATCCTGCAGCGTGACC
TGGGTGGGGCCCCCGGTTAATCTAATATCCCATTTGAGATTTTGTTTTTCCCTTTCCTGGC
CCAGGTTCAGGCTCCTGGGAAGATGCAGCTTTGCTGACGGAGGCCAACCTGCCTGCTCC
TGCTCCTGCTTCTGCTTCTGCCCCTGTCCTAGAGACTCTGGGCAGCTCTGAACCTGCTGG
GGGTGCTCTCCGACAGCGCCCCACCTGCTCTAGTTCCGGATCtGGCGGAGGAGGGAGCG
GGGGAGGCGGATCTGGCGGAGGCGGaTCCGGCTTCTCTGAGACCGTGGACCTGATGCTG
AACCTGCAGTCCAATAAGGAGGGCTCTGTGGATCTGAAGAACGTGAGCGCCGTGCCTA
AGGAGAAGACCACACTGAAGGACCCATCCAAGCCCCCTGCCAAGGCACAGGTGGTGG
GATGGCCACCCGTGCGGAACTACAGAAAGAATATGATGACCCAGCAGAAGACAAGCTC
CGACTACAAAGACCATGACGGTGATTATAAAGATCATGACATCGATTACAAGGATGACG
ATGACAAGGGCTCCGGCGCCACCAACTTTTCTCTGCTGAAGCAGGCCGGCGATGTGGA
GGAGAATCCTGGCCCAGGAGCCAAGCCACTGAGCCAGGAGGAGAGCACACTGATTGA
AAGAGCAACTGCCACTATTAACAGCATTCCCATTAGCGAGGACTATTCTGTGGCAAGCG
CCGCCCTGAGCTCCGACGGAAGGATCTTCACCGGCGTGAACGTGTACCACTTTACAGGC
GGCCCCTGCGCCGAGCTGGTGGTGCTGGGAACCGCAGCAGCAGCAGCAGCAGGCAATC
TGACATGTATCGTGGCCATCGGCAACGAGAATAGGGGCATCCTGTCCCCATGCGGCCGG
TGTAGACAGGTGCTGCTGGATCTGCACCCTGGCATCAAGGCCATCGTGAAGGACTCTGA
TGGACAGCCAACCGCCGTCGGGATTAGAGAACTGCTGCCATCAGGATACGTCTGGGAG
GGCTAGTGTACAAGTAGAGAAAAGGGGCAGACTCCTCACATTCCAGCACTTTCCCACCT
GACTCCTCTCCCCTCGTTTTTCCTTCAATAAACTATTTGTGTCAGCTTCTTCCTTGACTC
TGAAATGGGTTCAGGGCCATTTGAACACCCTGTGTCCCCCATGCATCTCTCACTTTCCGC
CATTAGAGGGGAGGTCTTCTTGTCCAGGGGAAGGCCTCCAGACCTGGGCTATTTTAAGC
ACAGATGTTTCCATTCATAAGCCTAGGAAGTCCAAGGAGGGAGAAGCTATCCTGGCCCC
TAGCAAGCTTTCCCACTCCCTGCATGTGACTTTCATCTCCCCCAGAGGAGGGGGATGGT
GTAGGGCAGGTCTGCAGAGCTGGGAGACATCATTCTGGCCCCCTCTACACCCCTCCCTT
CCTCCCTTCCTCCCTTTCTCTGTCCATTGGGAGGCAGAAGACAGGAAATGACTGCCATG
GATGTGGTAGGATTTTTACCAAAACAGCATTTTGCGGCTCAGGCCCGTGCTGTTCGCAG
CTGTTTTCCTCTAATATCAGCCAGGTGGCCGCCTTCTATGTTCCCCTCCCCATCCCGTTGT
ATGACTTCAGCCTTCGGATTCTAAGCTCCTGGCTGCTTTCCACATTCCACTCCCCTTCCA
GAACCCAGGCATCCTGGGCTCCAGCTGAAACCATTGCATGTGGCTTTCCCCATCCCTGG
CCCCGTGACTCAGTCCCTCTGAAGGGAGCAGCCCTCTTTTTGGCAATCACCAGGGAGG
TGGGGGGAGGAGGAGGGGAGCTAGGTGGTGACATCACAGTCGAAGGTTATAAAGCTT
CCAGCCAAACGGCATTGAAGTTGAAGATACAACCTGACAGCACAGCCTGAGATCTTGG
GGATCCCTCAGCCTAACACCCACAGACGTCAGCTGGTGGATTCCCGCTGCATCAAGGCC
TACCCACTGGTGAGGAGCTGCTATGGGCCAGAGAGGAGGAGGGAGGCATGCAGGGGCC
AGGACTCCTTGGACAGGATTTGCACtGATAGCAGGTGG

6- PEX3.C- miniIAA7.3xFlag.P2A.BSD

LA (left arm)-linker.miniIAA7.3xFlag.P2A.BSD-RA (right arm)

CCTTACACTATAGTGTATATGTGTGGTTCATGTATGTGTGTATATGTAAAATATGCACTTAC
TACACATATTCATACCGTATGTTAGATCTTCTAGAAAGTAATACACCCTTAACCGTGTTATG
TCAGCTTCCAGCAAAAGTACCTTTACTCCTACAATTATTTATTCAACAGATACCTATCTAG
CACTTGACATACACCAAAATTCATTTGAAGACTATTTGGGGTTTTTGTCCCTCAAAGTCA
CACTTGAACGCTTATGATACCATCATCCCTCATATAAGAACCTAAATAATAACTATCTCCTA

GAATTTATTACAAGAAAAACTACTCATTTTCTTAAATGGTTTGCCTCTTGGCCTTTACCAC
AAAACTTAGAGCTGAATTCATCGCTTGATTGCAGAAATTAATTCAAATTAGCTATATGTTT
TGCAAACTATAATGTTATATTATCATCTTTGCTAGGATCTGTTGACAATGGAGCAAGTGAA
AGACTTTGCTGCTAATGTGTATGAAGCTTTTAGTACCCCTCAGCAACTGGAGAAATTCtGG
CGGAGGAGGGAGCGGGGGAGGCGGATCTGGCGGAGGCGGaTCCGGCTTCTCTGAGACC
GTGGACCTGATGCTGAACCTGCAGTCCAATAAGGAGGGCTCTGTGGATCTGAAGAACGT
GAGCGCCGTGCCTAAGGAGAAGACCACACTGAAGGACCCATCCAAGCCCCCTGCCAAG
GCACAGGTGGTGGGATGGCCACCCGTGCGGAACTACAGAAAGAATATGATGACCCAGC
AGAAGACAAGCTCCGACTACAAAGACCATGACGGTGATTATAAAGATCATGACATCGAT
TACAAGGATGACGATGACAAGGGCTCCGGCGCCACCAACTTTTCTCTGCTGAAGCAGGC
CGGCGATGTGGAGGAGAATCCTGGCCCAGGAGCCAAGCCACTGAGCCAGGAGGAGAG
CACACTGATTGAAAGAGCAACTGCCACTATTAACAGCATTCCCATTAGCGAGGACTATTC
TGTGGCAAGCGCCGCCCTGAGCTCCGACGGAAGGATCTTCACCGGCGTGAACGTGTAC
CACTTTACAGGCGGCCCCTGCGCCGAGCTGGTGGTGCTGGGAACCGCAGCAGCAGCAG
CAGCAGGCAATCTGACATGTATCGTGGCCATCGGCAACGAGAATAGGGGCATCCTGTCC
CCATGCGGCCGGTGTAGACAGGTGCTGCTGGATCTGCACCCTGGCATCAAGGCCATCGT
GAAGGACTCTGATGGACAGCCAACCGCCGTCGGGATTAGAGAACTGCTGCCATCAGGAT
ACGTCTGGGAGGGCTAGTGTACAAGTGATTTTTCCTTCAAGAAAAACTACAGTGGGATT
CATTTACTTTTTAAAATACACTGGGTAAATCACCTATACTTAGAGTAACAGTTTGTTATCA
AAATGCCTGATAAAATATATTCTTAATAAAAGTCTTCATTTCATAATGAAATCAATTTATTT
GGCATCTTAATATATTTTTTTAGATTCATCAACAGACCAGTTTTGTGGGCATATATATATA
CACGTGCAAATATCAGAATTGTTAATAATTTGTTACACATGGACATTTGTTCCAAACTGAC

7- DHC1.C- miniIAA7.3xFlag.P2A.BSD

LA (left arm)-linker.miniIAA7.3xFlag.P2A.BSD-RA (right arm)

TCCTGTTTGCCCCAAAAGCCCTAATTGCCAGGAACACTGAGCCACTTGTGTGACATGAG
GTTTTAGCCGCGGTTTTGCTTGGATGTGATTATGGTCCTCCAGAAAGACACACCTGGACT
AATTTGACCCTAGAAACTGCACGGTTCTGAGACATGCTCTGGACCAGCCTGAGCTAGAG
CAGATGTGGTGAGGGCGGCGCCAGGGGCATAAAGTGCAGCCTGGGAAAGGCAGTAGGT
GGAGCCGCCAGCCGCCTGTGTGGGCAGCCAGGATGCCTAGCACTTGCACATTTGTTCCA
TCTGTGCTGGGGGAGTTGTGAGAGCTGACACCCTGGGCTCTGTGTGCCTTGGCTGCAGG
GATCTTGCCTCGGAGCTGGTCCCACTACACGGTGCCTGCCGGCATGACCGTCATCCAGT
GGGTGTCCGACTTCAGCGAGAGGATCAAACAGCTGCAGAACATCTCACTGGCAGCTGC
ATCTGGTGGCGCCAAGGAGCTAAAGGTGAAGGCGCTCCTGACGAGTCTCGGGTGGTCA
GCAGCTGTCCTGGGCTGGGGTGGGAGTGGCTCTGGGGAAAAACACAGGGCCCAGGTCT
GACCTGAGCTCCTTCCCCTGGGGGCTGCTGCTTTCCACAGAACATCCACGTGTGCCTGG
GTGGCCTGTTCGTGCCTGAGGCGTACATCACTGCCACCAGGCAGTATGTGGCCCAGGCC
AACAGCTGGTCCCTGGAGGAGCTCTGCCTGGAAGTCAACGTCACCACCTCACAGGGCG
CCACCCTTGACGCTTGCAGCTTCGGAGTCACGGGTGAGTGGAGTCTCACAGAAAATACT
GGCTCTTTGCAGGTGACCTCGGTGGCCTGAGACCATTGTTCCCAGATACATGCACTTAG
GGTGACCGGCTGGCAGTTGGGTGGAGCCTCTGGGCGCCCTGTGACTGGGGTTTGTGTA
GCTGTTGTGTTCACCTCAGCCTGGGTTTTGGCTTCCGCCTCACAGGTTTGAAACTTCAA
GGGGCCACGTGCAACAACAACAAGCTGTCACTGTCCAATGCCATCTCAACCGCCCTTCC
CCTGACGCAGCTGCGCTGGGTCAAGCAGACAAACACCGAGAAGAAGGCCAGTGTGGT
AAGGAGGCACTGCCTTTCCCAGGCATTCTGCAGGGACCCCTGCGGTAACAAGGGCAGA

GGCGGCTCCTCTTCTATGCCTGGGTTCCACTTGGAACGGGAAATGAGGTTCACAGAGGA
AATCCATTTCGCACCTGTCCAAACCTCTCCTTGCCGGGCCCCATCAGCTGTCCCGGGCA
GTCTTCCAGTTTTCTTACTTTTCCCTTAAGCCACCAGTAAACCCCTCTGCTTCTGCAGGT
AACCTTACCTGTCTACCTGAACTTCACCCGTGCAGACCTCATCTTCACCGTGGACTTCGA
AATTGCTACAAAGGAGGATCCTCGCAGCTTCTACGAGCGGGGTGTCGCAGTCTTGTGCA
CAGAGGGATCtGGCGGAGGAGGGAGCGGGGGAGGCGGATCTGGCGGAGGCGGaTCCGG
CTTCTCTGAGACCGTGGACCTGATGCTGAACCTGCAGTCCAATAAGGAGGGCTCTGTGG
ATCTGAAGAACGTGAGCGCCGTGCCTAAGGAGAAGACCACACTGAAGGACCCATCCAA
GCCCCCTGCCAAGGCACAGGTGGTGGGATGGCCACCCGTGCGGAACTACAGAAAGAAT
ATGATGACCCAGCAGAAGACAAGCTCCGACTACAAAGACCATGACGGTGATTATAAAGA
TCATGACATCGATTACAAGGATGACGATGACAAGGGCTCCGGCGCCACCAACTTTTCTC
TGCTGAAGCAGGCCGGCGATGTGGAGGAGAATCCTGGCCCAGGAGCCAAGCCACTGAG
CCAGGAGGAGAGCACACTGATTGAAAGAGCAACTGCCACTATTAACAGCATTCCCATTA
GCGAGGACTATTCTGTGGCAAGCGCCGCCCTGAGCTCCGACGGAAGGATCTTCACCGGC
GTGAACGTGTACCACTTTACAGGCGGCCCCTGCGCCGAGCTGGTGGTGCTGGGAACCG
CAGCAGCAGCAGCAGCAGGCAATCTGACATGTATCGTGGCCATCGGCAACGAGAATAG
GGGCATCCTGTCCCCATGCGGCCGGTGTAGACAGGTGCTGCTGGATCTGCACCCTGGCA
TCAAGGCCATCGTGAAGGACTCTGATGGACAGCCAACCGCCGTCGGGATTAGAGAACT
GCTGCCATCAGGATACGTCTGGGAGGGCTAAACTTTTCTAGCTGCCCCTTTCTGTAATAG
TGAAAGTTGGTATTTAACATTTATTCATTTTTAAAATATTTGGAAGGTCTGAGCTTGTGAA
AAGAAAGTGGTTGGTCTGAGGTTGGAGGAAGCTGAATGGAATCTGACGGTTGGGAGTG
GTGGAAATTGGAAGGATACCAGGAGGTATTTGGGAAGGCCAATGGCGTGGCTCCTTTGA
GGAAATAAAACACTAAGCATGAGCCGGCTCCGCCTCTTCTGTCTCCGCTTTCATCCCAG
GGCACAGAGCCTTGCCTTCCATGCTGCCCAGGGAGGGCAGCCCACGGCAGCCATGCCC
CTCCCCACCTCGCTTTCATCATGAGCTCGCTCCCGAGCGGCCACAGCACTCATGAATGA
AGACCTTGGGGCCCTTCACAGACACAGATGCAGCCAGCTGTGGCTCTGAAGGCCCTGG
GGCCCGGGCACCATGGTTCACACCTTTAATCGCAGCACTTTGGGAGTCTGGGAGTTAAA
GACCAGCCTCGGCAACATAGTGAGACCCCGTCTCTACAGGAAATTAAATCAGGTGTGGT
GGTGCATGCCTGTAGTCCCAGCTACTTGAGGGCTGAGGTGGGAGGATCACCCAAGCCCA
AGAGGTCGAGGCTGCAGTGAGCTGTGATCTCACCACTGCACTCCAGCCTGGGTGACAG
AGCAAGACCCTGTCTCAAAAAAAAAAAAAGCTGGGCGTGGTGGCTCATGCCTGTAATC
CCAGCACTTTGGGAGGCCGAGGCGGGCGGATCACCAAATTAGCCGGGCATGGTGGCAC
ATGCCTGTAATCCCAGCTACTCGGGAGGCTGAGGCAGGAGAATTGCTTGAACCTGGGAG
GCGGAGGTTGCAGTGAGCTGAAAAAAAAAAAGGCAGCCCCCAGCCGCTTGTGTTCTTG
ACCAGGGCCCCAGGACTTGGCTCCTCCAGACAAGGGAGTTTGTGCTGTAGATGAGGG
AGTTGCCCATCGCCGCCCTAGCAAGTCCATTCCCACACGACCTTTCCAGTGGTGATGATG
ACAGTGGCCCATACAGCTGACTGTTTGCATCTCACGTTCACATTGCTAGAGGTGATGGGT
GTGCTACACCCGTGGAAACAGGCTTCTGGCATCTCAGTGT

8- Glut1.C- miniIAA7.3xFlag.P2A.BSD

LA (left arm)-linker.miniIAA7.3xFlag.P2A.BSD-RA (right arm)

CCCCAGGAATGTTCTGCATCCTGCCACAGGCGGACTGTGCTGCAGGCACAGACAGGGG
ACCATGGGGCCTCTTAGCACCGTCCACATGCACACCACATTACCACTCCCAGCGGGTGG
ACTCAACACTTGCAGCCACCCTGCAGGACTTAAATCATTTGTTCGGATGAGGAAACTT

AGGCCCAATGCTGGATACACAACAAATCCAGGACCATCCCCAGGCTTCCTGCCTTCTCG
CATAGCTCTGCTCTGGCCTCTGTAGCTTCTGTTTCCCCTGAGGATCCATCACAACCCAGT
CTAACTTTTCCCCCTCTCCGTCATCCTCAACAGGAGCAGCTACCCTGGATGTCCTATCTG
AGCATCGTGGCCATCTTTGGCTTTGTGGCCTTCTTTGAAGTGGGTCCTGGCCCCATCCCA
TGGTTCATCGTGGCTGAACTCTTCAGCCAGGGTCCACGTCCAGCTGCCATTGCCGTTGC
AGGCTTCTCCAACTGGACCTCAAATTTCATTGTGGGCATGTGCTTCCAGTATGTGGAGGT
GAGAACCCCCACTGTCTCTATACTCACCCGCACGCATGACCCCACAGTGCTGTGCAAAG
TGCTGGCCCAGCATTCTCATTAGGAATCATGCTGAGGAGGAAACTGAGGGTAGGAAGCC
CAGGTCACATGACCAAGAATGCTGACCTCAACTCCAGGGTTCTCCCCTTAGCCTTGAAT
GGTGCTCCTGGAGCCTGTTACATTCTAGGTACAAGCGTGGTCTCAGTTAATTATGATAGC
ATCCCTGGGTAGGTGGATGTTCCCTTTTCAGCTCAAAGGCCCAAAGGTAAAGTGACTTAT
CCAAAGTCCTACAGCCAGGATGTAGGGTCATGACTCCAACCAAGTGTGTCTGTGTGTCT
TTCAGCAACTGTGTGGTCCCTACGTCTTCATCATCTTCACTGTGCTCCTGGTTCTGTTCTT
CATCTTCACCTACTTCAAAGTTCCTGAGACTAAAGGCCGGACCTTCGATGAGATCGCTTC
CGGCTTCCGGCAGGGGGGAGCCAGCCAAAGTGACAAGACACCCGAGGAGCTGTTCCAT
CCCCTGGGGGCTGATTCCCAAGTGGGATCtGGCGGAGGAGGGAGCGGGGGAGGCGGAT
CTGGCGGAGGCGGaTCCGGCTTCTCTGAGACCGTGGACCTGATGCTGAACCTGCAGTCC
AATAAGGAGGGCTCTGTGGATCTGAAGAACGTGAGCGCCGTGCCTAAGGAGAAGACCA
CACTGAAGGACCCATCCAAGCCCCCTGCCAAGGCACAGGTGGTGGGATGGCCACCCGT
GCGGAACTACAGAAAGAATATGATGACCCAGCAGAAGACAAGCTCCGACTACAAAGAC
CATGACGGTGATTATAAAGATCATGACATCGATTACAAGGATGACGATGACAAGGGCTCC
GGCGCCACCAACTTTTCTCTGCTGAAGCAGGCCGGCGATGTGGAGGAGAATCCTGGCC
CAGGAGCCAAGCCACTGAGCCAGGAGGAGAGCACACTGATTGAAAGAGCAACTGCCA
CTATTAACAGCATTCCCATTAGCGAGGACTATTCTGTGGCAAGCGCCGCCCTGAGCTCCG
ACGGAAGGATCTTCACCGGCGTGAACGTGTACCACTTTACAGGCGGCCCCTGCGCCGA
GCTGGTGGTGCTGGGAACCGCAGCAGCAGCAGCAGCAGGCAATCTGACATGTATCGTG
GCCATCGGCAACGAGAATAGGGGCATCCTGTCCCCATGCGGCCGGTGTAGACAGGTGCT
GCTGGATCTGCACCCTGGCATCAAGGCCATCGTGAAGGACTCTGATGGACAGCCAACCG
CCGTCGGGATTAGAGAACTGCTGCCATCAGGATACGTCTGGGAGGGCTAGTGTACAAGT
GAGTCGCCCCAGATCACCAGCCCGGCCTGCTCCCAGCAGCCCTAAGGATCTCTCAGGAG
CACAGGCAGCTGGATGAGACTTCCAAACCTGACAGATGTCAGCCGAGCCGGGCCTGGG
GCTCCTTTCTCCAGCCAGCAATGATGTCCAGAAGAATATTCAGGACTTAACGGCTCCAG
GATTTAACAAAAGCAAGACTGTTGCTCAAATCTATTCAGACAAGCAACAGGTTTTATAA
TTTTTTTATTACTGATTTTGTTATTTTTATATCAGCCTGAGTCTCCTGTGCCCACATCCCAG
GCTTCACCCTGAATGGTTCCATGCCTGAGGGTGGAGACTAAGCCCTGTCGAGACACTTG
CCTTCTTCACCCAGCTAATCTGTAGGGCTGGACCTATGTCCTAAGGACACACTAATCGAA
CTATGAACTACAAAGCTTCTATCCCAGGAGGTGGCTATGGCCACCCGTTCTGCTGGCCTG
GATCTCCCCACTCTAGGGGTCAGGCTCCATTAGGATTTGCCCCTTCCCATCTCTTCCTACC
CAACCACTCAAATTAATCTTTCTTTACCTGAGACCAGTTGGGAGCACTGGAGTGCAGGG
AGGAGAGGGGAAGGGCCAGTCTGGGCTGCCGGGTTCTAGTCTCCTTTGCACTGAGGGC
CACACTATTACCATGAGAAGAGGGCCTGTGGGAGCCTGCAAACTCACTGCTCAAGAAG
ACATGGAGACTCCTGCCCTGTTGTGTATAGATGCAAGATATTTATATATATTTTTGGTTGTC
AATATTAAATACAGACACTAAGTTATAGTATATCTGGACAAGCCAACTTGTAAATACACCA
CCTCACTCCTGTTACTTACCTAAACAGATATAAATGGCTGGTTTTTAGAAACATGGTTTTG
AAATGCTTGTGGATTGAGGGTAGGAGGTTTGGATGGGAGTGAGACAGAAGTAAGTGGG
GTTGCAACCACTGCAACGGCTTAGACTTCGACTCAGGATCCAGTCCCTTACACGTACCT
CTCATCAGTGTCCTCTTGCTCAAAAATCTGTTTGATCCCTGTTACCCAGAGAATATATACA

TTCTTTATCTTGACATTCAAGGCATTTCTATCACATATTTGATAGTTGGTGTTCAAAAAAA
CACTAGTTTTGTGCCAGCCGTGATGCTCAGGCTTGAAATGCATTATTTTGAATGTGAAGT
AAATACTGTACCTTTATTGGACAGGCTCAAAGAGGTTATGTGCCTGAAGTCGCACAGTG
AATAAGCTAAAACACCTGCTTTTAACAATGGTACCATACAACCACTACTCCATTAACTCC
ACCCACCTCCTGCACCCCTCCCCACACACACAAAATGAACCACGTTCTTTGTATGG

9- NUP93.C- miniIAA7.3xFlag.P2A.BSD

LA (left arm)-linker.miniIAA7.3xFlag.P2A.BSD-RA (right arm)

ATAAATACTGTTGCCTGGGGAAAGAAGGGCTAAGCATGTATGTCGGGAAGGGAGAAAC
AGGAGGAATGAAAGGAAGGAAGAGGAAAGATGCATGGGAGGAAGAGAGCTGGATTGG
GACTGCACAGTCACAGCCCTTGCCTCCGGTGTCACAAGGGCTTCATGGGGCTCTGGAG
AGTCAGATCCCTGTGAAAGCAGATGGACAGAAACCAGCCAGAGAGAGAGGCTCAGAA
GATTGGAGCAGGCAGTTCTGAAGCTCAGGGCTGTGTCAAAAGCTAGCCAAATGTGTTG
GGGCGAGGCGGCTTGCCTGGCAAACCCATCTGCTTTTTGCTTAATAGATGGGTTTGGATG
CCTGTGGAACAGAGGCCTCGGGGGACGAGCTTTGTTAACTTTGTGTTATGTTGAAGGAA
TGTGACAGAGGAGGGTATGACTGTCATCCACCCATCAGGGATCTGTCCCTGACACGCTG
GGGTAGAGGATGGAAGAACATGGAATAGAGGATGGAAGAATATGGAATAGTGCCCTGAC
TCGAAAGTTAACCGATTTCCTTCCCTTCCTTCCCTTCTCTCTCAGCAACTCCGAAGTCAA
GCCCGCACTCTGATTACCTTTGCTGGAATGATACCATACCGAACGTCTGGGGACACCAAT
GCGAGGCTGGTGCAGATGGAGGTCCTCATGAATGGATCtGGCGGAGGAGGGAGCGGGG
GAGGCGGATCTGGCGGAGGCGGaTCCGGCTTCTCTGAGACCGTGGACCTGATGCTGAAC
CTGCAGTCCAATAAGGAGGGCTCTGTGGATCTGAAGAACGTGAGCGCCGTGCCTAAGG
AGAAGACCACACTGAAGGACCCATCCAAGCCCCCTGCCAAGGCACAGGTGGTGGGATG
GCCACCCGTGCGGAACTACAGAAAGAATATGATGACCCAGCAGAAGACAAGCTCCGAC
TACAAAGACCATGACGGTGATTATAAAGATCATGACATCGATTACAAGGATGACGATGAC
AAGGGCTCCGGCGCCACCAACTTTTCTCTGCTGAAGCAGGCCGGCGATGTGGAGGAGA
ATCCTGGCCCAGGAGCCAAGCCACTGAGCCAGGAGGAGAGCACACTGATTGAAAGAGC
AACTGCCACTATTAACAGCATTCCCATTAGCGAGGACTATTCTGTGGCAAGCGCCGCCCT
GAGCTCCGACGGAAGGATCTTCACCGGCGTGAACGTGTACCACTTTACAGGCGGCCCCT
GCGCCGAGCTGGTGGTGCTGGGAACCGCAGCAGCAGCAGCAGCAGGCAATCTGACATG
TATCGTGGCCATCGGCAACGAGAATAGGGGCATCCTGTCCCCATGCGGCCGGTGTAGAC
AGGTGCTGCTGGATCTGCACCCTGGCATCAAGGCCATCGTGAAGGACTCTGATGGACAG
CCAACCGCCGTCGGGATTAGAGAACTGCTGCCATCAGGATACGTCTGGGAGGGCTAGTG
TACAAGTAAGTGCCATGCTTTGTGGGAGTCTGGGTCGGCACACTGTCAGTACATCAGGC
ACATGGGCCCACTAGGCTGGGGTTTCTGGTTTTGTTTCTGTTGTGTTTTGTTTTGGTTTCT
GTATTATGTATTTTTGTCAACGCCAATAAATTTCTTTGATTTGTATTTCTTTTCAACATTCTT
TTATTTTCTTTTTTTTTTTCTTTGAAATTTTGTTTACATTTTTATTTGTGTAATGGAAATAC
CGTCACTAGCTCTTGGGCCAGGGAATGAGAGGCTATGTAGATATTCATTATTTGGTTAAAT
TGACCTTAATTAATTAAAAATCTACCCAAAATGAGCCAGGAAACAAAGCCTTTGGGAGT
TACTTTTATTTAGATATAATATTCCATATAGCAGAGTCACGATTCATTTTCACATTTTTATTT
GAATGTGAAAGTCAACCTCAGCCTTAGAAGATGTAAGATTTGTGTGTGAAATATCAAGC
ACTCCATGTCCTCCTCTAGAGAGTGTGACTTAAGTCCAGATGGTCCAAGACCATCCCCC
AAATCATCAGCTCTGATTGCAGGATTCATCAGTTTGCCGTTCTGTTGCAATCCCAGTGGA
GATAAAAGCATAGACCTAGAAAAAGCCTTAAGAAGGAAGGTTGCAAGTGCCCCCAGCT
TGCACTTGAGGAAGGGGTAAACCTTGCCACTGCACTCCTCTGTAGCTGCTCCACTGTGA

GTTGACTGCTCGGCCTTGTACACACCCCTCCTAGAAAGACGTTTAAGCCCCTATTTCCCT
TCTCTGGTGGTCAGTTCCTCTGCTCAGACGACCCCCTTCCCCACTCATTAAGGTAATTGG
CCAGGCAGGCTCATGGGTCACGTGCTGAGTCTGCAATGTACAGAATCCAGACC

10- SEC61B.N-BSD.P2A.miniIAA7.3xFlag

LA (left arm)-BSD.P2A.miniIAA7.3xFlag.linker-RA (right arm)

AACCCCAGCAATCTGCAACTTTAAATGGGCCCACACTAAAGTTAGAGAACCACAGGCTC
GCTCACAACCCTGACTTCTCCATGTCAGTTCCGATCTTTGCGAACCGCAGACAGGGAAG
GTCTTCTCTCAGGGGTCATGCCCGCGGCCGCCCTCCACGGCGAGGTCCGCACTCGCGCA
GCCGGCCCCGCGGCCGCCTCACCTGGTCGCACACTACCACGTCGAACTCCTCGTCGGCG
AGGAACAGCACGTAGAGCGCCAGGAAAACCATGCGCACGTAGGCGCAGACGGCGGCG
CCGCGGCCGCCCCAGCCCAGGCCTCGCGGCAGCCAGTCCCCGGCACAGCGCACCGGTA
GCTCGCGGCTCTCGGCGAAACAGTGGCCCGGGTCGTAGTGCGCTGTCCAGATCTTCACG
CTACACCCGCGCGCCTGCAGCGCCAGCGCCGCGTCCAACACCAGCCGCTCAGCGCCGC
CCACGCCCAGGTCTGGGTGGAGGAACAGCACCGACGGCTTGGGAACCGAGTCCCGTTC
CCGGCCCTGCTCCTCCGCCATGGCCCTGGAGCCGCAACTGCACCCCGCACCCTGATGGG
GGTCTTCTGCGCAAGCTCCGCGCTCGTAGCTCCCAGCTGGCCACTGCGGGCCGACCCCG
CCCTGCCGTACGTGCGTCAGTTAGGCCACATCAGCGCAAATCTGTGAGGGTCTAGTAAC
TGCCTGAGAAAATATCTTGTCTGACCCCGGTTATATTTTTCCTTCGGTAGGGATTGGACTT
TCTGAAGGACGTTGTGATCCAAAGGAAGGAGGCCGGAGGTCTCTACTTCCCATACAGCA
GGTAACTAAGTTGTCTGTAGCAGACTGTCTACAGGCATATCGTGAGACGACCCAGGCGT
CCCTGGGGTCAGAGAGGACCTTGCCTGCAAGTCCGGGGGCGGGGCCTGAGTCAGTCTC
GCCAGCTGCCGGTCTTTCGGGGGCTCCGTAACTTTCTATCCGTCCGCGTCAGCGCCTTGC
CACCCTCATCTCCAATATGcctaggGGAGCCAAGCCACTGAGCCAGGAGGAGAGCACACTG
ATTGAAAGAGCAACTGCCACTATTAACAGCATTCCCATTAGCGAGGACTATTCTGTGGCA
AGCGCCGCCCTGAGCTCCGACGGAAGGATCTTCACCGGCGTGAACGTGTACCACTTTAC
AGGCGGCCCCTGCGCCGAGCTGGTGGTGCTGGGAACCGCAGCAGCAGCAGCAGCAGG
CAATCTGACATGTATCGTGGCCATCGGCAACGAGAATAGGGGCATCCTGTCCCCATGCGG
CCGGTGTAGACAGGTGCTGCTGGATCTGCACCCTGGCATCAAGGCCATCGTGAAGGACT
CTGATGGACAGCCAACCGCCGTCGGGATTAGAGAACTGCTGCCATCAGGATACGTCTGG
GAGGGCGGCTCCGGCGCAACCAACTTTTCTCTGCTGAAGCAGGCCGGCGACGTGGAAG
AAAATCCAGGCCCCggcttctctgagaccgtggacctgatgctgaacctgcagtccaataaggagggctctgtggatctgaagaac
gtgagcgccgtgcctaaggagaagaccacactgaaggacccatccaagccccctgccaaggcacaggtggtgggatggccacccgtgcgg
aactacagaaagaatatgatgacccagcagaagacaagctccGactacaaagaccatgacggtgattataaagatcatgacatcgattacaag
gatgacgatgacaagtccggaGGcGGAGGGAGCGGGGGAGGCGGATCTGGCGGAGGCGGaTCCATG
GTATGGCGGCCCTTCCATGATCCCCGCCTCTCCCAGAAGCCCTGACTCCTCCTGCTTTGC
GCCGTGCTTTTCCTCTGTAGCTCCCTTGCTTCCCCCAGCCTCGGGTGTGGGTGTCTAGGC
CGGGGTTCTGGGGCAGGCCTGCCGCGCTCACCCGTCTGTCTGCTTGTCTCCCTCTACAG
CCTGGTCCGACCCCCAGTGGCACTAACGTGGGATCCTCAGGGCGCTCTCCCAGCAAAGC
AGTGGCCGCCCGGGCGGCGGGATCCACTGTCCGGCAGAGGTAAGGAACCCTGCAGTTC
GTTCGCTTCCAGACTCGGAGATAGGACCCAGAACCTCGCTGATTCTGGGGTGGAGACCC
TAGCATGTGAAGATTGACAAAGGCAAAATGAGCTTCTAGTGACGTGGCCGTGGGAGTAG
TTAAAGGCCTTTTGGGAGGAAGGCGACATTTTTTTCTCGTTGCTCAGTTTAGGGCACTA
CTCTTAAAAAAGGAAAGTTAACAAACTGGAATAGAGTCAGAGATAACTTTGAGAAAAC
CGATGTCATTAAACTGGTGTCTCTGGACCTGAGGTTTGCACTCACATTTCCATCTGGCGG

CCCCATAAGCAATCTGTCCTACAGATAACTCGTCCTACACAAAACTTAGTCTCTTTTCAG
CTCAGCTCTCTCACTCTCAATTATATCTCCTTACTTCCATATGGCACTGTTGTACACTCATT
TACTCAGAGCCAGAAACGTCAGCGTCATCTTGGATTTTTCTTATGCTCTTTCTCTCTCTAG
TCATATGCCAGACTTTAAACTCTGCTTGAAAGCTTTCTCATAAGCTCTTTCCTTTTCCCTT
TCTACTGCTTTGCATTTGCTACTTAACCCTTTTCTTCAGGCTGTTTGCTTTCCAGTCCATC
GTTCGCTCTGCTGTTACTCTTCTGCGTAGTTTCTGTTACTTGTTGCTGAACTAAAAAAA
CAAACAAACAAAAAAAAAAAACCGGGGGGATACCAAAGCTCTTAGTGGTCATTTCAGC
CTAACCTTTCTTTTTCCTTTTTTTTTTTTTGGGATGGAATCTCACTCTGTCGCCCAGGCTG
GAGTGCAGTGGCACGATCTCGGCTCACTACAGCCTTTGCCTCCAGAGTTCAAGCAATTC
TCCTGCCTCAGCCTCCTGAGTAGCTGAGACTGCAGGCACGTGCCACCACACCCAGCTAA
TTTTTTGTATTTTTAGTAGAGACGGGGTTTCACCATGTTAGCCAGGATGGTCTCGATCTCC
TGACCTTGTGATCCTCCGTCTCTGCCTCCAAAAGTGCTGGAATTACAGGCGTGAGCCAC
TGCGCCCGGCCTTCAGCCTAATCTTTCTTTGCTGTCCTATGATCCAGCACTCGTGAACCC
ATTACTGTTTTCTGAATTCACAAACTTTCTCCCTCATCTCAAGGCAAATTTCTACTTATTTT
CCAAGACTAAGCTCAGGGGTTATTTCCTCTTGAGGCCTTCTCTGTCTGTAAGCAGCAGTA
GCTACTAGTTTTCCCTTTTGCCCCCA

11- POGZ.C-miniIAA7.3xFlag.P2A.BSD

LA (left arm)-linker.miniIAA7.3xFlag.P2A.BSD-RA (right arm)

TACTGGCTATGCTTAGTGCCTCTAGCACTTTGCCTGCAGTGGTCCCAGCAGGCTGTAGCT
CCAAAATTCAGCCATTAGATGTATGCATCAAAAGAACTGTCAAGAACTTCCTGCATAAAA
AATGGAAGGAACAGGCTCGGGAAATGGCAGATACTGCATGTGATTCTGATGTCCTGCTT
CAGCTGGTGCTTGTCTGGCTGGGTGAAGTGCTAGGTGTCATTGGGGACTGTCCAGAGCT
AGTTCAGCGCTCCTTCCTGGTGGCTAGTGTTCTGCCTGGCCCCGATGGCAACATTAACTC
ACCTACAAGAAATGCTGACATGCAGGAGGAGCTAATTGCCTCCCTAGAGGAGCAACTGA
AGCTGAGTGGGGAACATTCTGAGTCTTCCACTCCACGACCCAGATCATCTCCTGAAGAG
ACAATTGAGCCTGAAAGTCTTCACCAGCTCTTTGAGGGTGAAAGTGAGACCGAGTCTTT
CTATGGCTTTGAAGAAGCTGACCTAGATCTGATGGAGATTGGATCtGGCGGAGGAGGGA
GCGGGGGAGGCGGATCTGGCGGAGGCGGaTCCGGCTTCTCTGAGACCGTGGACCTGAT
GCTGAACCTGCAGTCCAATAAGGAGGGCTCTGTGGATCTGAAGAACGTGAGCGCCGTG
CCTAAGGAGAAGACCACACTGAAGGACCCATCCAAGCCCCCTGCCAAGGCACAGGTGG
TGGGATGGCCACCCGTGCGGAACTACAGAAAGAATATGATGACCCAGCAGAAGACAAG
CTCCGACTACAAAGACCATGACGGTGATTATAAAGATCATGACATCGATTACAAGGATGA
CGATGACAAGGGCTCCGGCGCCACCAACTTTTCTCTGCTGAAGCAGGCCGGCGATGTG
GAGGAGAATCCTGGCCCAGGAGCCAAGCCACTGAGCCAGGAGGAGAGCACACTGATT
GAAAGAGCAACTGCCACTATTAACAGCATTCCCATTAGCGAGGACTATTCTGTGGCAAG
CGCCGCCCTGAGCTCCGACGGAAGGATCTTCACCGGCGTGAACGTGTACCACTTTACAG
GCGGCCCCTGCGCCGAGCTGGTGGTGCTGGGAACCGCAGCAGCAGCAGCAGCAGGCA
ATCTGACATGTATCGTGGCCATCGGCAACGAGAATAGGGGCATCCTGTCCCCATGCGGCC
GGTGTAGACAGGTGCTGCTGGATCTGCACCCTGGCATCAAGGCCATCGTGAAGGACTCT
GATGGACAGCCAACCGCCGTCGGGATTAGAGAACTGCTGCCATCAGGATACGTCTGGGA
GGGCTAGTGTACAAGTGAGTGTTGGGGTCATGAGGGGGTGTGGAGTGAGGGTGGGAAC
ATGTGAGGGAGGGTAAAGGGGCTTAGGGAAAAGGGGGCATACCAGGTGGGGTATTTGG
TTTCTATTTTTTAATTTTATACCACCACTCCCCCCTGAAGTTGACTTACACTTCCCTGTGG

ATTTGTGGATTAATTAGGAAAACCAATAGTAATCACGTCTGAGCCAAGGAGCTGGCCCAT
TGGTCATTCACTTCTGCTAAAAACAGGTTTTTGTGACTTTTTTTTTTTTTAAATTTAAATC
ACTGTGTTTGGTATTTTTCTGACAAAATTAAGAAAAAGAAAAAAAATTATTTGTGGGCA
AATGTTAAATTTTTTTGTTTCCCCTTTTACCTCAATTGTATCATAGTACTGGGTTTTTTTGT
TTGTTTTATTGTGTGGCCAATGTCTTTGGGCATGATGCTA

12- RABGGTA.N-miniIAA7.mEGFP

LA (left arm)-miniIAA7.mEGFP.linker-RA (right arm)

GGAGAAGCCAGAATGGGGGTGTCAAGTATAAAGAAATGGGTAGCGTCAAATGCTTCAG
AGGTTAGAGAAAATGACAGGAAACTCCCCCTGTTAGATTTGACATCTAGAAGGCTGTCG
GTGTGGCCAGGGTGAGGTCAGTGGAGTAGTGAAGGTGCGGGTTTCCGGCTGTTGGAGA
AACCTCTTCCACTAACTACATTGCACTCTGAGTTACCAACTGGCATGGTCCTCCCCCCAT
TTCACACGCACGCTACAGTCGCCCGTGCGCACTCGTACACACCAGGTGGCTAACAGGTA
CGAGGCGTTTCCGGACAGCGAGCGCAGAGCCCCCGCGCACGCGCCTCCGAGCCGGCCT
CTTCCGCCGCAAGTCCCTCCCCAAGCCTGCAGAGTGCGCTGCGTTTCGATGAGCCGGGA
CGTGGCGCCGCTCTAGCCAGCGCCTGGGCTCTGTGGCGGGCGCCGCAGCTCCGCGTCCC
CCGCGCCTCCTCCCAGCGCAGGTGGGTCCGCCCGCGGGGGGCGGGGGTGCCCGGGAGC
CGTGGGACGGGCGGGTAGCGGCAACCTTTGGGGAGCAGAGTGGTCCCCAACGCCCAGG
TGCCCCGGCTCGCGCTCTGGCCCGGGCGAATCGGGCTATAGGAAGGGCCACACGGATG
GAAGTCCTAGTCCGGGTGCTCACCTCTTGTGGAACGTGCAAAGCCTGTCCCAGGACCTC
TTTACACTCTGGGGGTCTCTGCCCAGGCACGCTTGCTGCTTCCGGACACAGCTGTGGGC
GGAGCTAGTAGGGGCGGGGCTACGTGATTGACACTTCTCTCCTCAGACTTCAAGGGCTA
CCACTGGACCCTTCCCCTGTCTTGAACCCTGAGCCGGCACCATGcatggcttctctgagaccgtggacc
tgatgctgaacctgcagtccaataaggagggctctgtggatctgaagaacgtgagcgccgtgcctaaggagaagaccacactgaaggaccca
tccaagccccctgccaaggcacaggtggtgggatggccacccgtgcggaactacagaaagaatatgatgacccagcagaagacaagctcct
ccggaGTGAGCAAGGGCGAGGAGCTGTTCACCGGGGTGGTGCCCATCCTGGTCGAGCTGG
ACGGCGACGTAAACGGCCACAAGTTCAGCGTGTCCGGCGAGGGCGAGGGCGATGCCAC
CTACGGCAAGCTGACCCTGAAGTTCATCTGCACCACCGGCAAGCTGCCCGTGCCCTGGC
CCACCCTCGTGACCACCCTGACCTACGGCGTGCAGTGCTTCAGCCGCTACCCCGACCAC
ATGAAGCAGCACGACTTCTTCAAGTCCGCCATGCCCGAAGGCTACGTCCAGGAGCGCA
CCATCTTCTTCAAGGACGACGGCAACTACAAGACCCGCGCCGAGGTGAAGTTCGAGGG
CGACACCCTGGTGAACCGCATCGAGCTGAAGGGCATCGACTTCAAGGAGGACGGCAAC
ATCCTGGGGCACAAGCTGGAGTACAACTACAACAGCCACAACGTCTATATCATGGCCGA
CAAGCAGAAGAACGGCATCAAGGTGAACTTCAAGATCCGCCACAACATCGAGGACGGC
AGCGTGCAGCTCGCCGACCACTACCAGCAGAACACCCCCATCGGCGACGGCCCCGTGC
TGCTGCCCGACAACCACTACCTGAGCACCCAGTCCAAGCTGAGCAAAGACCCCAACGA
GAAGCGCGATCACATGGTCCTGCTGGAGTTCGTGACCGCCGCCGGGATCACTCTCGGCA
TGGACGAGCTGTACAAGaccGGaGGcGGAGGGAGCGGGGGAGGCGGATCTGGCGGAGGC
GGaTCCGTGAGAACCCTGAGCCCAGCCCTTCACCTCCGCAGGCCCGCGCCCTCACCCCC
AGTGGCAGTGCCATGGTTGCCCCGTATGAACCTAGGGCTCCACAGTGTGGGGTTTTTGC
AGGTCAGCTTCTGCAAGGCAACCTGCACTCTTCCAACCCGTTCATCCTTGTAGCACGGA
CGCCTGAAGGTGAAGACGTCAGAAGAGCAGGCGGAGGCCAAAAGGCTAGAGCGAGAG
CAGAAGCTGAAGCTATACCAGTCAGCCACCCAGGCCGTATTCCAGAAGGTGGGGCCCTC
AGAGGTAGCCCCGACCCCAAGTAGCTCCCTTAGTATAGTGCAGAGGCCAAGAGCTGGTC
CCTAGAGTCAGACTGCCTAGATCCAATTCTGGGTTATCTCACTTATAACTGTATGACTTTG

GGCAAGTTGCTGAATCTCTTTGGGCCTCAGTTTGCTCATCTGTAAAATGGGGCTAATAAT
TGCACCTACTGCATAAGGCTGTTGTGAAGATTAAATGAGAGAATACCTGTAAGGCCATAA
AGCATGGTTCATAGTACCTGTTGTATAATGTGGGATTCACAGAATATTGGATGGTTTTAAG
TTTACGTTATTTAGTCCTGTAGTGGGAAAATTATATGATCTCTAACCCTGCCACCTATCTTC
TTAGCGCCAGGCTGGTGAGCTGGATGAGTCCGTGCTGGAACTGACAAGCCAGATTCTGG
GAGCCAACCCTGATTTTGCCACCCTCTGGAACTGCCGACGAGAGGTGCTCCAGCAGCT
GGAGACTCAGAAGTGCGTAGGGATTCAGGATCTCAGAAGACTGCCCTGGCCCTGCGCC
TGCACTGCCTGGGTCTAGGGGTGGAGGAGAAATGGGGCAGCCAGTGGCATAGATGGAG
GATAGTAGGGGGCTGGGTGCAGAAAGTGAGGGGTGAGCTGAGGTTGACTGTAGGAGAG
CCCTGACC

13- LMNA.N-Sh_ble.P2A.miniIAA7.3xFlag

LA (left arm)-Sh_ble.P2A.miniIAA7.3xFlag.linker-RA (right arm)

GACTGTGGCTTGTTGCTTGGGTCTAAAAACGAATGCTTGGCTTTGAAGAGAGATAGATT
GGGGCAAAAGAAAGAAAAAAAGGGACCCCCCAAACTCCTTGATCCCTGGCCCCAAACT
GGGGGCATAAAGGAACTCAGGTTCCAGAACTTTGCTCCCCCCAGGGAACCCAGGCATT
CCTTCTCCACCCCACTCCTGGCACACTGAGATGCAGCTCTGAATGGGCTGCCCACGTGT
GGAGGGGGGTTGGGGTGACTCACTATTACTACTGGGAGGACAGGGGGAGCCAGTGGTG
GAAGAAGGGTGAGTCACACTGATGGGCACCAGCCTCAGCCCTCCCCCCACTTTCCTGG
CTCCCAGCCCTGCCTACCTGACCCTCTCCCTTGCTTTGCGCCCACTTCCCTCTCTTTCTCC
CCGACCCTTTTGCCCACCCACTCTCCCTCCTTGGCTCTGCCCTCTAGCCCAGAAGGTCTG
AGGCAATGGGGGCAAGCTTGGAGCCGACAGTGCTGAGCAGGCAGGAGCCAAGAGAGG
GGAAGCTTGAGCCTCACGCAGTTAGGGGTGCGCTGGAGAGGGTGGGGCCCGACTCCGC
CACACCCCAACGGTCCTTCCCCCTCCTCACCACTCCCGCCCCCACCCCCAATGGATCTG
GGACTGCCCCTTTAAGAGTAGTGGCCCCTCCTCCCTTCAGAGGAGGACCTATTAGAGCC
TTTGCCCCGGCGTCGGTGACTCAGTGTTCGCGGGAGCGCCGCACCTACACCAGCCAACC
CAGATCCCGAGGTCCGACAGCGCCCGGCCCAGATCCCCACGCCTGCCAGGAGCAAGCC
GAGAGCCAGCCGGCCGGCGCACTCCGACTCCGAGCAGTCTCTGTCCTTCGACCCGAGC
CCCGCGCCCTTTCCGGGACCCCTGCCCCGCGGGCAGCGCTGCCAACCTGCCGGCCATGG
CAAAGCTGACCAGCGCCGTGCCTGTGCTGACAGCAAGGGATGTGGCAGGAGCAGTGGA
GTTCTGGACAGACCGGCTGGGCTTCAGCAGAGATTTTGTGGAGGACGATTTCGCAGGA
GTGGTGCGCGACGATGTGACCCTGTTTATCTCCGCCGTGCAGGACCAGGTGGTGCCAGA
TAACACACTGGCATGGGTGTGGGTGCGGGGACTGGACGAGCTGTACGCAGAGTGGAGC
GAGGTGGTGTCCACCAATTTCAGAGATGCCTCCGGCCCTGCCATGACAGAGATCGGAGA
GCAGCCATGGGGAAGGGAGTTCGCACTGCGCGACCCCGCAGGAAATTGCGTGCACTTT
GTGGCAGAGGAGCAGGATGGCTCCGGCGCAACCAACTTTTCTCTGCTGAAGCAGGCCG
GCGACGTGGAAGAAAATCCAGGCCCCggcttctctgagaccgtggacctgatgctgaacctgcagtccaataaggagg
gctctgtggatctgaagaacgtgagcgccgtgcctaaggagaagaccacactgaaggacccatccaagccccctgccaaggcacaggtggt
gggatggccacccgtgcggaactacagaaagaatatgatgacccagcagaagacaagctccGactacaaagaccatgacggtgattataaa
gatcatgacatcgattacaaggatgacgatgacaagtccggaGGcGGAGGGAGCGGGGGAGGCGGATCTGGCG
GAGGCGGaTCCGAGACCCCGTCCCAGCGGCGCGCCACCCGCAGCGGGGCGCAGGCCAG
CTCCACTCCGCTGTCGCCCACCCGCATCACCCGGCTGCAGGAGAAGGAGGACCTGCAG
GAGCTCAATGATCGCTTGGCGGTCTACATCGACCGTGTGCGCTCGCTGGAAACGGAGAA
CGCAGGGCTGCGCCTTCGCATCACCGAGTCTGAAGAGGTGGTCAGCCGCGAGGTGTCC
GGCATCAAGGCCGCCTACGAGGCCGAGCTCGGGGATGCCCGCAAGACCCTTGACTCAG

TAGCCAAGGAGCGCGCCCGCCTGCAGCTGGAGCTGAGCAAAGTGCGTGAGGAGTTTAA
GGAGCTGAAAGCGCGGTGAGTTCGCCCAGGTGGCTGCGTGCCTGGCGGGGAGTGGAG
AGGGCGGCGGGCCGGCGCCCCTGGCCGGCCGCAGGAAGGGAGTGAGAGGGCCTGGAG
GCCGATAACTTTGCCATAGTCTCCTCCCTCCCCGGAACTGCCCCCAGCGGGTGACTGGC
AGTGTCAAGGGGAATTGTCAAGACAGGACAGAGAGGGAAGTGGTGGTCTCTGGGAGA
GGGTCGGGGAGGATATAAGGAATGGTGGGGGTATCAGGGACAAGTTGGGGCTGGGGCC
GGCCTGAATTCGGTCAGATTGGGATTTGCCAACTATCTGGAGCCGGGGGGAGGGGCTTG
AGCAAAACAGAACTAGCCCTGCCAGCTCGAAGAACTCTGGGCACCCAGGACACATCGG
AGTGGCAGAAAGGGTCCTGTTAGAACTTTGTTAGCGGGCTTGGCACTGTGCTAGCTTTG
CCCAAGCTGGCTCTGAACACATGATGCCCACTAAGACATAACTCTCAAGTTGGCATCTG
TCCAGCGTGTTGGA

14- MYH9.N-Sh_ble.P2A.miniIAA7.3xFlag

LA (left arm)-Sh_ble.P2A.miniIAA7.3xFlag.linker-RA (right arm)

CCCAAAGGGAAGCACTCTTAGCATTCTCCATGTTTCTTGGAACCTGGCATCTTTTCTCTG
TGCTTTTTTTGGGCTGTATTATCAAGATGGAATTAAAATCTGGTGGCAACATGTACTGATG
TTTTATTTTCTTCTCTTGTCTTAGTTGACTTCATGAACTAATAACAAGTTTTTTAAACTGG
AAACTTCCACTATGTGGGGTTGTAGTCAGTTGTGTTTTTTGGCTGATTAGAAACCTGTTT
TTGTTGGAAATCCTCCCAAACTGTGTGGCTACTTTGCTACCTTTGTATCTGTAGCATAGCG
ACTTGAATTGCATCTGTCTTTGAGACTCTTGTTATGTTCATTTGGCACAATAGTTTTCTTG
ATTGTGGATTGACCCTAGGGCGATACACCTTACCATTAGTTGCTAACAAAATCCCTGCTAT
CAGCTTGTTTTTCTGTTGCCCAGGCTGGAGTGCAGTGGTGTGAGCTGGCTTACTGCAAC
CTCCGCCTCCTGGGTTCAAGCGATTCTCCTGCCTCAGCCTCCCGAGTAGCTAGGATGAC
AGGTGTGCACCACCACGCCTGGTTAATTTTTGTATTTTTAGTAGAGATGGGTTTTCACCA
GGTTGGCCAGGCTGGTCTTGAACTCCTGACCTCAGGTGATATGCCTACCTTGGCCTCCCA
AAGTGCTGGGATTACAGGCATGAGCCACTGTCCTGGGCCCAACTTGTTTTTTCAGTGAT
GCACATCTTTATCCCAAAAGTGTGGAGCAAGAAAGTTGTGCAGCCTGGTTATATAGAGTT
TTACTGTGTCTTAATCCTATACAGTTTGCAAAGAGAAGAGGTGTGAGCATGAGTGATCTT
GTGTGGCTGACGTAGTTGTATGTAATGTGTTGTCCTTCTCCTCCCCGCTTAGGTCCTGGCT
ATAAGTCACCATGGCAAAGCTGACCAGCGCCGTGCCTGTGCTGACAGCAAGGGATGTG
GCAGGAGCAGTGGAGTTCTGGACAGACCGGCTGGGCTTCAGCAGAGATTTTGTGGAGG
ACGATTTCGCAGGAGTGGTGCGCGACGATGTGACCCTGTTTATCTCCGCCGTGCAGGAC
CAGGTGGTGCCAGATAACACACTGGCATGGGTGTGGGTGCGGGGACTGGACGAGCTGT
ACGCAGAGTGGAGCGAGGTGGTGTCCACCAATTTCAGAGATGCCTCCGGCCCTGCCATG
ACAGAGATCGGAGAGCAGCCATGGGGAAGGGAGTTCGCACTGCGCGACCCCGCAGGA
AATTGCGTGCACTTTGTGGCAGAGGAGCAGGATGGCTCCGGCGCAACCAACTTTTCTCT
GCTGAAGCAGGCCGGCGACGTGGAAGAAAATCCAGGCCCCggcttctctgagaccgtggacctgatgct
gaacctgcagtccaataaggagggctctgtggatctgaagaacgtgagcgccgtgcctaaggagaagaccacactgaaggacccatccaag
ccccctgccaaggcacaggtggtgggatggccacccgtgcggaactacagaaagaatatgatgacccagcagaagacaagctccGactac
aaagaccatgacggtgattataaagatcatgacatcgattacaaggatgacgatgacaagtccggaGGcGGAGGGAGCGGGGG
AGGCGGATCTGGCGGAGGCGGaTCCGCACAGCAAGCTGCCGATAAGTATCTCTATGTGG
ATAAAAACTTCATCAACAATCCGCTGGCCCAGGCCGACTGGGCTGCCAAGAAGCTGGTA
TGGGTGCCTTCCGACAAGAGTGGCTTTGAGCCAGCCAGCCTCAAGGAGGAGGTGGGCG
AAGAGGCCATCGTGGAGCTGGTGGAGAATGGGAAGAAGGTGAAGGTGAACAAGGATG

ACATCCAGAAGATGAACCCGCCCAAGTTCTCCAAGGTGGAGGACATGGCAGAGCTCAC
GTGCCTCAACGAAGCCTCGGTGCTGCACAACCTCAAGGAGCGTTACTACTCAGGGCTC
ATCTACGTAAGTGGCTGCCGTGGCACCCCGCAGGCTGGGTCTGAGGGCTCCGAGGTGG
GGGGGGGGGCGGGTCTTCCCATCACCCTCACGTGCCTGGGCCCTGGCTCTCTGGTTGA
GAAGGGGGCTTGAAGGAGTGCTGGGGTGACATCCTTGAACTTTGACATCTGAACATCCT
AGTGGGATGTTCTTATAGCATTATTCTTTTCCCTTTGCAGCCAAAAGCAAAACAGGAAGC
CTGTTTTAGAACATACGTAGGGAGAAGAATATGGCCCTGTCTGAGGGATGGCCACCCAC
TGGCCTTCGTTCTCAAGTTTTGGTGGAAATATGAAGGTTCAGGTCAATGTT

15- LBR.C-miniIAA7.3xFlag.P2A.Sh_ble

LA (left arm)-linker.miniIAA7.3xFlag.P2A. Sh_ble -RA (right arm)

TTTGACCCTTTTAGCCGCCAGAAAGATGTGTGTGTGTGTCCTTTGTGTGGGACAGTGTG
GCCAAGGTGGGTGTGAGTCTTTTTGTCGCCCTTTTCTACTGTGAGGTTGGCCTCTGTAGA
AGCTCCTCTGGCTTCTGAGGGGAATTGGGAATTCACAGAGCCTGAAGTTTTACTTTAAG
CCTAAAAGCACTATTTTAAGTTTTCAGCTGTGCCTGATAGCTTTTTTCTAAGTAGAATATA
TAGAAATTAATTCAGCTTCTTAATTCATGATTCAGTGTACATAGATTGGGAAAAACTTTGG
TTTTTAAGAAGAGAAAGAGATTCTGTTTTTCTTTTTGTGTGTCTGGCCTCGTGGGGAGCT
GGGAAAGCAGGGGAGCAGGGAGAGCTGTGGGAGCTGGAGGTGCTTGGGGAGCTGGGG
TGTCAGGGGGAGCTGGAGGTACTGGTGGAGCCAGGGGAGCTGGGGGAACTGGGGGAG
CAGGGGGATCTTGGGAAGCTGGGGGAGCTGGGGGAACTGGGGGAGCAGGGGGATCCA
GGGGAGCTGGTGGAGGTGGAGGAGCTGGCCCGTGCCTGCTGCCTGTTTGGTGGCCCAG
CCTGTGGTGGAAGACAGCAGTTTTTCTTCTTTGCTGGGGGAAGAAGGGCGAGAAATGA
AGAGATGTGTATTAACTATGAGGACAGATGTTTTGATTGTGATGGATTGTTAGAATGGATT
CATCAGTCTTTGAGTAGCTTCTAATTTAGATACGACCTTCTGCCACTTTCCAGTGGGATTT
TACAGACTTGTTTAATTATTCTCATCATTATTAGAGAAAATGAAGTACCTTGTGGTTCCAT
TTTTAGAGAATTTGGGGGAAAGCAGGAAAAAAATACATTGTAGTGATAATAAAAATATCTA
ATACTGTGTACTATTATTATCCACAGGTTTTAACCACATTCTGCCTTATTTCTACATAATTTA
TTTCACCATGTTGCTTGTCCACCGAGAAGCTCGTGACGAGTACCACTGTAAGAAGAAAT
ACGGCGTGGCTTGGGAAAAGTACTGTCAGCGTGTGCCCTACCGTATATTTCCATACATCT
ACGGATCtGGCGGAGGAGGGAGCGGGGGAGGCGGATCTGGCGGAGGCGGaTCCggcttctct
gagaccgtggacctgatgctgaacctgcagtccaataaggagggctctgtggatctgaagaacgtgagcgccgtgcctaaggagaagaccac
actgaaggacccatccaagccccctgccaaggcacaggtggtgggatggccacccgtgcggaactacagaaagaatatgatgacccagcag
aagacaagctccgactacaaagaccatgacggtgattataaagatcatgacatcgattacaaggatgacgatgacaagGGAAGCGGA
GCGACGAATTTAGTCTACTGAAACAAGCGGGAGACGTGGAGGAAAACCCTGGACCTG
GAGCAAAGCTGACCAGCGCCGTGCCTGTGCTGACAGCAAGGGATGTGGCAGGAGCAGT
GGAGTTCTGGACAGACCGGCTGGGCTTCAGCAGAGATTTTGTGGAGGACGATTTCGCA
GGAGTGGTGCGCGACGATGTGACCCTGTTTATCTCCGCCGTGCAGGACCAGGTGGTGCC
AGATAACACACTGGCATGGGTGTGGGTGCGGGGACTGGACGAGCTGTACGCAGAGTGG
AGCGAGGTGGTGTCCACCAATTTCAGAGATGCCTCCGGCCCTGCCATGACAGAGATCGG
AGAGCAGCCATGGGGAAGGGAGTTCGCACTGCGCGACCCCGCAGGAAATTGCGTGCAC
TTTGTGGCAGAGGAGCAGGATTAATGCTCTTCTGGCTTTTCTACAAAATACTCCTGCAAT
TCCAGCTGCCATTTGCAAAAACAAGGAAAAAAATCCGAAACTTTCTTTTGTTGCACTGA
CAGGGTCTGTACTTTTTTTTTTCTTTTTGAGTCAGGACTATGGAGCCGAGTAGTTGATCTT
TTAATATAGCCGTGTTTACTTGTATTAACTTACAGTTAACATAGGAAAAATACAAGTAAGG
ATGTGAGAATTTGCATTTAATGGGAAATTTTCAACCCTTAATCTGAAAACAGAAGACAG

TCTTAATATAAATGTACTGTGAAGAATGCTATTGATGTTTATGGTTTCTGATTACTTTTCAA
ATTTTGATGTTTTTTTGCCAGTTGGCTTTTCTTAAATGAAAACACTGTTCCATTTAAAGTA
CATTTATGTTTTATTCAGTAAGAGAATAGAATTTTCATTTGTTTTTCTTTAAATCCTTTACT
AATTATATAATTTGAAAGCAAAAAGAAGGGCCTATATTAAATGCTGAAAGTGAAAAGTG
ATGACATTATTAGCAGACACTGC

16- LMNB1.N-miniIAA7.mEGFP

LA (left arm)-miniIAA7.mEGFP.linker-RA (right arm)

GCCCATTAACACTGTTGGCAAATGATTTAAAATCAGGGTTGCTGGTTTTTCTGTGATAATC
TATCGGCTTCAAGACGCACAGATCTCACTTTCATGGAAAGAAGTTGCCTGATACAGTTTT
CCACCGGAGAGAAAAATAAAGGCTGGTACTTGGAACCTGCAAGCCGTGCATTTGGAAC
CTCGGACTCAAGTGCCTATTACGTAATTCCACAGCGTCCCGGCCTCCAGGCCGTTTCCCG
AGCCCTCCAGCGGAGCGGGGGATAAGGTTACCACGCCCGCGGTGGCCGGGGACACTCT
GAGTTTCGCGTGTGGCTTTTAGGGACGTTTATATTTGAATTTCCCTGAACCGCCGAGTGT
GGGCGGTGGCGCAGATCCGTCCCGGAAACCTCCGGGCTCCTTCCCGCCTTTCTCAGGCC
CGGCCCCTCCAAGGGGTCCCCGCGGGGCGGCGGGAGGGCCCTGGGCCCAGAGCCGCG
CGGGTGGGCAGTCCCAGGCGTCCTTCCTTACAGCCCTGAGCCTGGTCCGGGAACCGCCC
AGCCGGGAGGGCCGAGCTGACGGTTGCCCAAGGGCCAGATTTTAAATTTACAGGCCCG
GCCCCCGAACCGCCGAAGCGCGCTGCCTGCTCCCCATTGGCCCATGGTAGTCACGTGGA
GGCGCCGGGGCGTGCCGGCCATGTTGGGGAGTGCGGCGCCGCGGCCCGCGCCACCTCC
GCCCCCCGCGGCTTGCCTCCAGCCCGCCCCTCCCGGCCCTCCTCCCCCCGCCCGCCGCT
CCGTGCAGCCTGAGAGGAAACAAAGTGCTGCGAGCAGGAGACGGCGGCGGCGCGAAC
CCTGCTGGGCCTCCAGTCACCCTCGTCTTGCATTTTCCCGCGTGCGTGTGTGAGTGGGT
GTGTGTGTTTTCTTACAAAGGGTATTTCGCGATCGATCGATTGATTCGTAGTTCCCCCCCG
CGCGCCTTTGCCCTTTGTGCTGTAATCGAGCTCCCGCCATCCCAGGTGCTTCTCCGTTCC
TCTAAACGCCAGCGTCTGGACGTGAGCGCAGGTCGCCGGTTTGTGCCTTCGGTCCCCGC
TTCGCCCCCTGCCGTCCCCTCCTTATCACGGTCCCGCTCGCGGCCTCGCCGCCCCGCTGT
CTCCGCCGCCCGCCATGctcggcttctctgagaccgtggacctgatgctgaacctgcagtccaataaggagggctctgtggatct
gaagaacgtgagcgccgtgcctaaggagaagaccacactgaaggacccatccaagccccctgccaaggcacaggtggtgggatggccacc
cgtgcggaactacagaaagaatatgatgacccagcagaagacaagctcctccggaGTGAGCAAGGGCGAGGAGCTGTT
CACCGGGGTGGTGCCCATCCTGGTCGAGCTGGACGGCGACGTAAACGGCCACAAGTTC
AGCGTGTCCGGCGAGGGCGAGGGCGATGCCACCTACGGCAAGCTGACCCTGAAGTTCA
TCTGCACCACCGGCAAGCTGCCCGTGCCCTGGCCCACCCTCGTGACCACCCTGACCTAC
GGCGTGCAGTGCTTCAGCCGCTACCCCGACCACATGAAGCAGCACGACTTCTTCAAGTC
CGCCATGCCCGAAGGCTACGTCCAGGAGCGCACCATCTTCTTCAAGGACGACGGCAACT
ACAAGACCCGCGCCGAGGTGAAGTTCGAGGGCGACACCCTGGTGAACCGCATCGAGCT
GAAGGGCATCGACTTCAAGGAGGACGGCAACATCCTGGGGCACAAGCTGGAGTACAAC
TACAACAGCCACAACGTCTATATCATGGCCGACAAGCAGAAGAACGGCATCAAGGTGA
ACTTCAAGATCCGCCACAACATCGAGGACGGCAGCGTGCAGCTCGCCGACCACTACCA
GCAGAACACCCCCATCGGCGACGGCCCCGTGCTGCTGCCCGACAACCACTACCTGAGC
ACCCAGTCCAAGCTGAGCAAAGACCCCAACGAGAAGCGCGATCACATGGTCCTGCTGG
AGTTCGTGACCGCCGCCGGGATCACTCTCGGCATGGACGAGCTGTACAAGtccGGaGGcG
GAGGGAGCGGGGGAGGCGGATCTGGCGGAGGCGGaTCCGCGACTGCGACCCCCGTGCC
GCCGCGGATGGGCAGCCGCGCTGGCGGCCCCACCACGCCGCTGAGCCCCACGCGCCTG

TCGCGGCTCCAGGAGAAGGAGGAGCTGCGCGAGCTCAATGACCGGCTGGCGGTGTACA
TCGACAAGGTGCGCAGCCTGGAGACGGAGAACAGCGCGCTGCAGCTGCAGGTGACGG
AGCGCGAGGAGGTGCGCGGCCGTGAGCTCACCGGCCTCAAGGCGCTCTACGAGACCGA
GCTGGCCGACGCGCGACGCGCGCTCGACGACACGGCCCGCGAGCGCGCCAAGCTGCA
GATCGAGCTGGGCAAGTGCAAGGCGGAACACGACCAGCTGCTCCTCAAGTGAGTGCTA
GCTGGCGGCCGCGTTAGCGCCAAGGAGGGGCGGGGGCGCAACCGCGGCGACCAGCTC
ACCGGGTTCTGCCGTGGGGAGGGAGCAGAGGCCAGGATGCACGCGTCCTTCTGAAGGA
ACAGGGTCTCGGTCTCCGGAAAGGAGAAAGAATCTAGAGTTCATAGCGGAGCAGGGGT
CGCGGAGGGGGCTCGAGCTGTAGCGCTGGGGGGCCGTGATGCCCATTTCTAGATTTGG
ATACCCGCTGGGACGTGGTAAGTGCGCGCCTGGGACTGCCGAGAAGGAGCTCCCGCTT
TCGCACTCGAATCCGGGGAGCCGGCGCGGAGAGGCGGCCCCTCAGGCCCCAGGTGCGG
GGAGCTGGAGCGCGAGCGCGCGCTCGCGTGCGCGCCCCAGTTTCCGGCCGGCGCGAGA
CAAAGCGTCTAGCGGATTTGCAGTGCCGGGATGGGCGGCCGGGGAGGACTGGCAGCCC
GCCTCTAGAATGAATGAGCTTCGCGCGGGCAGAGAGAGGAAGGGGAGGGACCTTCCCG
CAGCATCCGCGTCTCCTGGGGGTGGGTCCCGCTTTGGCGCGCTCAGTCTTGGCCCTGTG
ACGTTTTGCGAAGATTCTACGCCTGCTTTAGGCGGAGAGAGAGGCGGAGCTTGATCCG
TGCGCCTCCAGCCAGCCCCGGGCAAGTTAGGTTTGCTAGCTGGTTAGCTTTCTCTGGGG
AAATGTGTCCTGTCCCAGCGGGGATGGTGCGGTCCCTCGGGTGGTTCTGCGCAGGGAA
GGAAAGCTGGGTTTGTGCATGTCAC

HDR enhancers

EF1a_**i53**_HSV TK poly(A)

ggctccggtgcccgtcagtgggcagagcgcacatcgcccacagtccccgagaagttggggggaggggtcggcaattgaaccggtgcctag
agaaggtggcgcggggtaaactgggaaagtgatgtcgtgtactggctccgcctttttcccgagggtgggggagaaccgtatataagtgcagta
gtcgccgtgaacgttctttttcgcaacgggtttgccgccagaacacaggtaagtgccgtgtgtggttcccgcgggcctggcctctttacgggttat
ggcccttgcgtgccttgaattacttccacctggctccagtacgtgattcttgatcccgagctggagccaggggcgggccttgcgctttaggagcc
ccttcgcctcgtgcttgagttgaggcctggcctgggcgctggggccgccgcgtgcgaatctggtggcaccttcgcgcctgtctcgctgctttcga
taagtctctagccatttaaaatttttgatgacctgctgcgacgctttttttctggcaagatagtcttgtaaatgcgggccaggatctgcacactggtattt
cggtttttggggccgcgggcggcgacggggcccgtgcgtcccagcgcacatgttcggcgaggcggggcctgcgagcgcggccaccgaga
atcggacgggggtagtctcaagctggccggcctgctctggtgcctggcctcgcgccgccgtgtatcgccccgccctgggcggcaaggctggc
ccggtcggcaccagttgcgtgagcggaaagatggccgcttcccggccctgctccagggggctcaaaatggaggacgcggcgctcgggaga
gcgggcgggtgagtcacccacacaaaggaaaggggcctttccgtcctcagccgtcgcttcatgtgactccacggagtaccgggcgccgtcca
ggcacctcgattagttctggagcttttggagtacgtcgtctttaggttggggggaggggttttatgcgatggagtttccccacactgagtgggtgga
gactgaagttaggccagcttggcacttgatgtaattctccttggaatttgccctttttgagtttggatcttggttcattctcaagcctcagacagtggttc
aaagtttttttcttccatttcaggtgtcgtgaacacgggctgcagaagttggtcgtgaggcactgggcaggtaagtatcaaggttacaagacaggtt
taaggagaccaatagaaactgggcttgtcgagacagagaagactcttgcgtttctgataggcacctattggtcttactgacatccactttgcctttct
ctccacaggtgtccactcccagttcaattacagctcttaaggctagagtacttaatacgactcactataggctagcctcgagaattcacc**atgGG
AGACTACAAGGATGACGATGACAAGGGATCTGCCGGCAGTGCTGCCGGAAGCGGC
GAGTTCCTGATCTTCGTGAAGACACTGACTGGCAAGACCATCACACTGGAAGTGG
AGCCTTCCGACACCATTGAGAACGTGAAGGCTAAGATTCAGGACAAGGAGGGCAT
TCCTCCAGACCAGCAGAGGCTGGCCTTCGCTGGCAAGTCTCTGGAGGATGGAAGG
ACTCTGTCTGACTACAACATCCTGAAGGACTCCAAACTGCACCCCCTGCTGCGGC
TGCGCCtga**tcgatGCGGGACTCTGGGGTTCGAAATGACCGACCAAGCGACGCCCAACCTGC
CATCACGAGATTTCGATTCCACCGCCGCCTTCTATGAAAGGTTGGGCTTCGGAATCGTTT

TCCGGGACGCCGGCTGGATGATCCTCCAGCGCGGGGATCTCATGCTGGAGTTCTTCGCC
CACCCTAGGGGGAGGCTAACTGAAACACGGAAGGAGACAATACCGGAAGGAACCCGC
GCTATGACGGCAATAAAAAGACAGAATAAAACGCACGGTGTTGGGTCGTTTGTTCATAA
ACGCGGGGTTCGGTCCCAGGGCTGGCACTCTGTCGATACCCCACCGAGACCCCATTGGG
GCCAATACGCCCGCGTTTCTTCCTTTTCCCCACCCCACCCCCCAAGTTCGGGTGAAGGC
CCAGGGCTCGCAGCCAACGTCGGGGCGGCAGGCCCTGCCATAGCCTC

P53DD (mouse P53 Δ14-301aa.)
atgACAGCCATGGAAGAGAGTCAGTCTGACATCTCCCTGAAAAGAGCCCTGCCAACTTGT
ACATCAGCCTCTCCTCCCCAGAAAAAGAAGCCACTGGATGGGGAGTACTTCACTCTGAA
GATCAGGGGAAGAAAGCGGTTTGAGATGTTCAGAGAGCTGAATGAAGCCCTGGAGCTG
AAGGATGCCCACGCTACAGAAGAGTCTGGAGACTCAAGAGCTCACTCTTCCTACCTGA
AGACCAAGAAAGGCCAGTCTACAAGCCGGCATAAAAAGACAATGGTGAAGAAAGTGG
GACCAGACTCTGATtga

Table S3: sgRNA sequences

Target	sgRNA*	PAM**
SAC1/SACM1L	GTGGTTGTGCAGGATGGCGA	cgg
FSP1	GAGGCAGTCTCCACCTTGA	tgg
BSCL2/Seipin	TGCTCTAGTTCCTGAAGAAA	agg
RANGAP1	TCTGCTGCAGACGCTGTACA	agg
	GGGAGAGGCTTTGAGTCT	AGA
PEX3	AAAATCATTTCTCCAGTTGC	TGA
	CCCCTCAGCAACTGGAGAAA	TGA
DHC1	CTTTCTGTAATAGTGAAAGT	tgg
Glut1	TCCCAAGTGTGAGTCGCCCC	AGA
	GATCTGGGGCGACTCACACT	tgg
SEC61B	TGCCACCCTCATCTCCAATA	tgg
	GGGCCGCCATACCATAT	tgg
NUP93	AAAGCATGGCACTTAATTCA	TGA
	GCATGGCACTTAATTCATG	agg
ACSL4	AAGACATTGAACGAATGTAT	ggg
	GACATTGAACGAATGTATG	ggg
POGZ	TCTGATGGAGATTTGAGTGT	tgg
LMNA	ACGGGGTCTCCATGGCCGGC	agg
	CCATGGAGACCCCGTCCCAG	cgg
MYH9	ATCGGCAGCTTGCTGTGCCA	tgg
	GTCCTGGCTATAAGTCACCA	tgg
LBR	TACATCTACTAATGCTCTTC	tgg
	GAAGAGCATTAGTAGATGTA	tgg
RABGGTA	CAGGGTTCTCACCATGGTGC	cgg
LMNB1	GCTGTCTCCGCCGCCCGCCA	tgg
	CACGGGGGTCGCAGTCGCCA	tgg

*start codon and stop codon are underlined; first sgRNAs have higher efficiencies and were used to generate single-cell clones

**for NGA PAM, Cas9/QRVR was used.

Table S4: PCR primers for genotyping PCR

Target	Sense	Antisense
SAC1/SACM1L	GCTCTGCCTTTCCCCAAAAC	GCTTCGGTACCCAGATAGGC
FSP1	TGGGGAGAAATGACGGTGTG	CTAGCCACCACATCATTGGC
BSCL2/Seipin	CAGGCTCCTGGGAAGATGCA	CATGGGGGACACAGGGTGTTC
RANGAP1	CCAAAATGGCCCCCACACTT	CACATACTCAGGGGACTGACA
PEX3	ATGGTTTGCCTCTTGGCCTT	GTCAGTTTGGAACAAATGTCCATGT
DHC1	CCCCTCTGCTTCTGCAGGTA	GGAGCCGGCTCATGCTTAGT
Glut1	TCCTGAGACTAAAGGCCGGA	TTGCTGGCTGGAGAAAGGAG
SEC61B	CTGGGGTCAGAGAGGACCTT	TGGGAGAGCGCCCTGAGGAT
NUP93	TGTCATCCACCCATCAGGGA	AGGCTTTGTTTCCTGGCTCA
ACSL4	CCATGGACCCCTGAAACTGG	AAAACCACCAGGCTACCTCC
POGZ	GAGTCTTCCACTCCACGACC	CTCCTTGGCTCAGACGTGAT
LMNA	CAATGGATCTGGGACTGCCC	CTTCAGACTCGGTGATGCGA
MYH9	AGAAAGTTGTGCAGCCTGGT	CTTGGGCGGGTTCATCTTCT
LBR	TGTTGCTTGTCCACCGAGAA	ACAGACCCTGTCAGTGCAAC
RABGGTA	TATAGGAAGGGCCACACGGA	CTTCTGCTCTCGCTCTAGCC

Table S5: Arm-spanning primers for genotyping PCR

**Target**	**Sense**	**Antisense**
SAC1/SACM1L	GTGAGCAAAGCGGCATCAAA	CCGAGATTAGCCTCTGGCTG
BSCL2/Seipin	GTGGTCAGGGGTATGTTGGG	TAGGGACGCTGGAATCCTCC
RANGAP1	GACTCTAGTGGGCCAGTCCT	CCCGAGACTATCCCGCTTTC
ASCL4	GCCTGTCGGAAAAGCACAGT	CCCTGCAGCACTTTTGCTTT
DHC1	ACGGTAGTAGGTTACGGCCT	ACACTGAGATGCCAGAAGCC
Glut1	CACGCCTTAGAACCTGAATCCA	TACAGAGGAACCAGAGGGGG
SEC61B	CTCAGGGCACCTGGATGAAA	TGTCATGCCTTATGGTGGGT
NUP93	CATCCTGAACAATCCAGGAA	CTGATGCCATGTGGTGGTCT
