# Supplementary material for: HiHo-AID2: boosting homozygous knock-in efficiency enables robust generation of human auxin-inducible degron cells
Source: Genome Biol. 2024 Feb 26;25:58. doi: 10.1186/s13059-024-03187-w (PMC10895734; doi:10.1186/s13059-024-03187-w)
Supplement: Supplementary file 5 — Additional file 5. Review history. [file 13059_2024_3187_MOESM5_ESM.docx]

Review History

**First round of review**

**Reviewer 1**

**Were you able to assess all statistics in the manuscript, including the appropriateness of statistical tests used?** No.

**Were you able to directly test the methods?** Yes.

**Comments to author:**

This is an excellent submission showing the following:

Used A-431 cell line (epithelial morphology from a patient with epidermoid carcinoma) to show that:

1. Use Co-incident insertion ("Co-IN") to insert:

a) AtAFB2 (F74A) (relative of OsTIR1, which they also insert in some experiments) at AAVS1 and

b) mAID miniIAA7-3xFlag at the target gene (in some examples they targeted the N-terminus instead of C-terminus).

* Ratio used of each was 1:3

2. Enhanced gene targeting using two additives:

a) Co-transfection with a plasmid encoding "i53" (https://www.addgene.org/74939/ - published here: https://www.nature.com/articles/nbt.4021), which inhibits 53BP1

b) Addition of 0.25 μM MG3814, which is a DNA-dependent protein kinase (DNA-PK) inhibitor

* Note: 1 μM alone is also OK, but causes more cell death.

3. Small-size Blasticidin S deaminase (BSD) outperforms other selections used (used Co-IN with Puro).

4. Combining 1-3 achieves one-step generation of AID cells with high-efficiency homozygous tagging

* Repeated 1 to 4 with lung alveolar cancer A549, embryonic kidney HEK293A, and osteosarcoma U2OS cell lines.

Then used H9 hESCs:

1. AtAFB2(F74A) under the EF1a promoter was unstable in H9 cells, leading to inefficient inducible degradation. CAG promoter 226 improved both stable expression of AtAFB2(F74A)-mCherry

2. i53 plus 0.25 μM M3814 increased the efficiency of degron-tagging.

o M3814 at 1μM showed severe cytotoxicity.

3. They also show that P53DD, a dominant negative P53 mutant, can enhance targeting efficiency, but this is not a good idea in my opinion.

Comments:

* More commentary of the findings in Figure Supplemental S1 is needed.

* Explain the target function of M3814 and i53

* Line 297 and 412 (and elsewhere) - correct I53 to i53

* What are the hESC targeting results for 0.25 μM M3814 alone (without i53 and P53DD)

* Explain the decreased efficiency of protein depletion (RANGAP1 and PEX3) in hESC-derived neurons.

* Differentiation to neurons is only showing one cell type/lineage being applicable to this approach. What about examples and results from others?

* In many of the graphs it is not clear whether statistical analyses were made. A table containing all statistical comparisons should be provided.

**Reviewer 2**

**Were you able to assess all statistics in the manuscript, including the appropriateness of statistical tests used?** Yes: All data are descriptive statistics. No statistics test were described in main and supplementary figures. Although the method section mentioned RNA sequencing analysis and DEG tests, I seems cannot find any RNA sequencing data presented in the main text and figures.

**Were you able to directly test the methods?** No.

**Comments to author:**

In this manuscript, the author presented a method based on CoIN, double selection and HR enhancing chemicals or genes to generate homozygous AID fusion tagged cell lines. The method can be useful if clearly and convincingly described. However, I have several serious concerns:

1. The most critical concern is that the author did not attempt to perform genomic quality control in their knock-in clones. CRISPR-Cas9 based gene editing can introduce off-target mutations that could have phenotypical consequence. Moreover, all the HR enhancer used in this manuscript could increase the level of mutations (DNA-pk inhibitor and 53BP inhibitor i53) in the final clones or allow clones with normally non-survivable DNA damage to remain viable (P53DD). Because of this, the knock-in clones need to be comprehensively characterized by whole genome sequencing to ensure that HiHo-AID2 is a viable method, rather than only appear to increase KI efficiency, but in fact creating more unpredictable mutations in cell clones.

2. The quality control of the on-target knock-in is not sufficiently stringent. The genotyping primers used to determine het or hom clones are both on the homology arms of the donor construct, thus will not only detect correct knock-in, but also random integrations of donor plasmids or on target concatemer insertion. These can all be mistakenly recognized as correct knock-in. They need to use arm-spanning primers to confirm correct insertion and perform copy number analysis by qPCR or ddqPCR .

3. The authors assumed that the clones they manually picked after the selection are single cell clones. However, there is no guarantee that is the case, the apparent clones can grow from a cluster of cells. The clonality needs to be tested.

4. The authors described RNA sequencing analysis in the method section. Yet there is no RNA seq data mentioned anywhere in the paper or figures.

5. The data presentation in the figures is confusing and without sufficient explanation. Here are the examples:

1. Why all cell counts, which is supposed to be a simple positive integer have to be presented as arbitrary unit (a.u.)? A direct presentation of cell count will be much easier to understand.

2. All the GFP positive cell data and the GFP intensity data in Figure 2-4 are presented as an average number. However, looking at the data points, they are very scattered, I am not sure an appropriate statistical test will reveal any significant difference. Furthermore, the author choose to present data in a converted form, for example the GFP intensity is not the real intensity but the % change, which artificially inflates perceived difference and creates hurdle for statistical analysis and interpretation. Flow cytometry can give absolute fluorescent intensity for experimental and control groups, there is no reason why each experiment cannot be presented with absolute fluorescent intensity subtracting control values, which will be more comparable intuitively and easier for statistical analysis.

3. The phenotypical effects of AID induced degradation of endogenous proteins are too simple. Only a morphological picture could not capture the phenotypical consequences of the protein degradations.

In summary, I believe the manuscript presented a interesting method that could be interested by researchers using AID in cell lines. However, study was not done and presented in a very rigorous way.

**Authors Response**

**Point-by-point responses to the reviewers’ comments:**

**Point-by-point responses to the Reviewers’ comments:**

**Reviewer #1:** GBIO-D-23-00756 Review

This is an excellent submission showing the following:
Used A-431 cell line (epithelial morphology from a patient with epidermoid carcinoma) to show that:
1.      Use Co-incident insertion ("Co-IN") to insert:
a)      AtAFB2 (F74A) (relative of OsTIR1, which they also insert in some experiments) at AAVS1 and
b)      mAID miniIAA7-3xFlag at the target gene (in some examples they targeted the N-terminus instead of C-terminus).
*       Ratio used of each was 1:3
2.      Enhanced gene targeting using two additives:
a)      Co-transfection with a plasmid encoding "i53" (<https://www.addgene.org/74939/> - published here: <https://www.nature.com/articles/nbt.4021>), which inhibits 53BP1
b)      Addition of 0.25 μM MG3814, which is a DNA-dependent protein kinase (DNA-PK) inhibitor
*       Note: 1 μM alone is also OK, but causes more cell death.
3.      Small-size Blasticidin S deaminase (BSD) outperforms other selections used (used Co-IN with Puro).
4.      Combining 1-3 achieves one-step generation of AID cells with high-efficiency homozygous tagging

*       Repeated 1 to 4 with lung alveolar cancer A549, embryonic kidney HEK293A, and osteosarcoma U2OS cell lines.

Then used H9 hESCs:
1.      AtAFB2(F74A) under the EF1a promoter was unstable in H9 cells, leading to inefficient inducible degradation. CAG promoter 226 improved both stable expression of AtAFB2(F74A)-mCherry
2.      i53 plus 0.25 μM M3814 increased the efficiency of degron-tagging.
o       M3814 at 1μM showed severe cytotoxicity.
3.      They also show that P53DD, a dominant negative P53 mutant, can enhance targeting efficiency, but this is not a good idea in my opinion.

Response: We wish to thank the Reviewer for the careful and insightful examination of our manuscript and for the constructive comments and suggestions provided. We are delighted that the Reviewer considers this manuscript ‘an excellent submission’ and are below providing point-by-point responses to the valuable comments that have helped us to significantly further improve the manuscript.

Comments:
*       More commentary of the findings in Figure Supplemental S1 is needed.

Thank you for the comment. We have now expanded the commentary on the findings related to Figure Supplemental S1 in **Supplemental Note 1, p 1-4**.

*       Explain the target function of M3814 and i53

We have now explained the functions of the targets of M3814 (DNA-PK) and i53 (53BP1) in the main text (**p. 5, line 119-120**) and in the **Supplemental Note 2 p 5, line 104 - 109**.

*       Line 297 and 412 (and elsewhere) - correct I53 to i53

Thank you for the careful reading of our manuscript. The text has been corrected.

*       What are the hESC targeting results for 0.25 μM M3814 alone (without i53 and P53DD)

This is a good question. To find this out, we transfected 3 targets in hESCs and compared control with treatment of 0.25 µM M3814 or i53. We measured the cell counts and analysed GFP intensities by FACS. This showed that 0.25 µM M3814 alone also causes severe cytotoxicity, and similarly as shown in Figure 4, i53 increases the efficiency of degron-tagging. We added graphs showing the percentage of GFP positive cells, the percentual change of single-cell GFP intensity and cell count (x10^5^ cells/ml) with these treatments to **Supplemental Figure 8f** and described this in the main text (**p11, line 254 – 255**).

*       Explain the decreased efficiency of protein depletion (RANGAP1 and PEX3) in hESC-derived neurons.

We envision that this may be related to the expression and accessibility of *At*AFB2(F74A) to its substrates that might be compromised in neurons, due to the differentiation procedure and the complex compartmentalization of neurons (from our earlier study, Li et al. Nat Methods 2019, PMID: 31451765; it is evident that the compartmentalization of the auxin receptor between the cytoplasm and nucleus in non-neuronal cells has a major effect on target degradation). Moreover, prominent local regulation of proteasomal activity in neurons might limit the degradation efficiency after inducible poly-ubiquitination. We have now added these points to the Discussion (**page 16, line 379 – 384**).

*       Differentiation to neurons is only showing one cell type/lineage being applicable to this approach. What about examples and results from others?

Thank you for bringing up this important point. It is obviously not feasible to examine the approach in all possible differentiation directions from hESCs, but we have now included additional experiments demonstrating the effective degradation of target proteins in hESC derived embryoid bodies (EB) comprising the three embryonic germ layers. We have included data showing efficient differentiation of hESCs to EBs using α-SOX17 (endoderm), α-SMA (mesoderm) and Tubulin-BIII (ectoderm) immunostainings and have demonstrated efficient degradation of selected targets in two different degron cell lines (SAC1 and RANGAP1; immunoblotted for the degradation tag miniIAA7 and mesoderm marker α-SMA). In addition, we checked the functionality of the degron cell lines, by showing increased PI4P staining of the EBs 3h after SAC1 degradation and live imaging/cell death analysis of the RanGAP and SAC1 degron EBs at late times (>80 h) upon inducing degradation. These results are included in the text at **page 12 (line 285 – 297**), **Figure 5a-d** and **Supplemental Figure 8h**.

*       In many of the graphs it is not clear whether statistical analyses were made. A table containing all statistical comparisons should be provided.

We apologize for not having been clear about this. We have now performed statistical analyses of the graphs and provide an overview of all the statistical comparisons in **Supplemental Table 2**, as requested.

**Reviewer #2:**

In this manuscript, the author presented a method based on CoIN, double selection and HR enhancing chemicals or genes to generate homozygous AID fusion tagged cell lines. The method can be useful if clearly and convincingly described.

We wish to thank the expert Reviewer for the careful and thoughtful examination of our manuscript and the insightful comments and concerns raised. We are very pleased that the Reviewer feels that the method can be useful and have now carefully revised the manuscript to describe it more clearly and convincingly.

However, I have several serious concerns:

1.      The most critical concern is that the author did not attempt to perform genomic quality control in their knock-in clones. CRISPR-Cas9 based gene editing can introduce off-target mutations that could have phenotypical consequence. Moreover, all the HR enhancer used in this manuscript could increase the level of mutations (DNA-pk inhibitor and 53BP inhibitor i53) in the final clones or allow clones with normally non-survivable DNA damage to remain viable (P53DD). Because of this, the knock-in clones need to be comprehensively characterized by whole genome sequencing to ensure that HiHo-AID2 is a viable method, rather than only appear to increase KI efficiency, but in fact creating more unpredictable mutations in cell clones.

Thank you for this important question. Indeed, CRISPR-Cas9 based gene editing is a relatively new method and the issue regarding potential off-target effects pertains to most if not all of the current papers reporting on its use. We admit that this is a technology under development and full-proof exclusion of any unwanted effects is challenging. Indeed, many methods have been developed the detect the off-target sites of CRISPR/Cas9, each – including whole genome sequencing (WGS), as suggested by the Reviewer - with inherent advantages and disadvantages.

To assess the potential unpredictable/unwanted mutations in an unbiased manner in this manuscript, we carried out WGS sequencing of 5 samples focusing on the most important application in hESCs (2 wild-type H9 cells and 3 degron cell lines underwent WGS). The analysis carried out by the company CeGaT showed that no off-target effects were found in the H9 hESCs. In addition, no variations were found in the TP53 gene in these cell lines. The WGS results are provided in **Supplemental Table 1** and **Supplemental Figure 9**. We discuss the WGS data in the main text on **p 11, line 262 – 271 and p 15, line 369 – 370,** The WGS data set will be made publicly available in the NCBI Sequence Read Archive data repository upon publication under the accession number: PRJNA1050771, and you can access the data via this link:

https://dataview.ncbi.nlm.nih.gov/object/PRJNA1050771?reviewer=m6pql2vi0e05lc8cakj3smvbsp

2.      The quality control of the on-target knock-in is not sufficiently stringent. The genotyping primers used to determine het or hom clones are both on the homology arms of the donor construct, thus will not only detect correct knock-in, but also random integrations of donor plasmids or on target concatemer insertion. These can all be mistakenly recognized as correct knock-in. They need to use arm-spanning primers to confirm correct insertion and perform copy number analysis by qPCR or ddqPCR.

Thank you for this valuable remark. We have now performed genomic PCR with arm-spanning primers as suggested, in the 5 different cell lines used (A431, A549, HEK293A, U2OS, and H9 hES cells) using multiple targets. These genomic PCR results provided in **Supplemental Data 1** show correct insertion in the expected knock-in site. With long-range PCR, we also detected additional deletions at on-target sites in 2 out of 16 (A431 cells),1 out of 8 (A549), 1 out of 6 (HEK293A) and 0 out of 5 (U2OS) clones, in agreement with the findings of Riesenberg et al. Nucleic Acids Res 2019, PMID: 31392986). This is now reported and discussed on **p. 8, line 184 – 189, p. 9, line 218 – 220, p. 11, line 261 – 262 and p. 14/15, line 347 - 350. Supplemental Data 1** also contains the genotyping results using primers on the homology arms, as the short PCR has its benefit for screening homozygous insertions due to its simplicity. The new WGS data added reveal that there are no indications of random integrations of donor plasmids or on target concatemer insertion and no copy number variations in the degron cell lines compared to the respective WT cells. These data are provided in **Supplemental Table 1** and added to the main text (**p 11, line 266 – 269**).

3.      The authors assumed that the clones they manually picked after the selection are single cell clones. However, there is no guarantee that is the case, the apparent clones can grow from a cluster of cells. The clonality needs to be tested.

Clonality is indeed an issue when picking apparent clones from cells that grow as groups. As cell clusters containing a fraction of cells with a non-tagged allele would give rise to a band at wild-type position, we excluded all clones with this band as heterozygous, as the first way to assess clonality. With the remaining clones, functional characterization of clones for several targets at single cell level provided no indication of contamination from wild-type or heterozygously tagged cells, arguing that these are indeed likely to originate from single cells. We have now clarified this point in the text (**p. 14, line 343 - 350**). Of note, some cells such as HEK293A do not form apparent cell clusters and may rather spread out to neighbouring locations. For such cells, limited dilution is recommended for single clone selection, as discussed in the manuscript (**p. 9, line 212 - 214**).

4.      The authors described RNA sequencing analysis in the method section. Yet there is no RNA seq data mentioned anywhere in the paper or figures.

The RNA seq data are provided in **Supplemental Fig. 1 m**. We have now clarified this by adding this information to the Method section (**p. 22, line 542**) and by adding a comment on the data in **Supplemental Note 1** (**p 3, line 59 – 61** and **line 63-67**). We have deposited the RNA seq data to the NCBI GEO database with accession number GSE243637. You can access the data via this link:

<https://www.ncbi.nlm.nih.gov/geo/query/acc.cgi?acc=GSE243637> by using the access token: **ghmbusiqvrcbjat**

5.      The data presentation in the figures is confusing and without sufficient explanation. Here are the examples:
1.       Why all cell counts, which is supposed to be a simple positive integer have to be presented as arbitrary unit (a.u.)? A direct presentation of cell count will be much easier to understand.

We apologize for the unclear/insufficient data presentation in the figures. We have now improved this as proposed. First, we have changed the graphs and presented the cell counts as cells x10^5^ or 10^6^ cells/ml.

2.      All the GFP positive cell data and the GFP intensity data in Figure 2-4 are presented as an average number. However, looking at the data points, they are very scattered, I am not sure an appropriate statistical test will reveal any significant difference. Furthermore, the author choose to present data in a converted form, for example the GFP intensity is not the real intensity but the % change, which artificially inflates perceived difference and creates hurdle for statistical analysis and interpretation. Flow cytometry can give absolute fluorescent intensity for experimental and control groups, there is no reason why each experiment cannot be presented with absolute fluorescent intensity subtracting control values, which will be more comparable intuitively and easier for statistical analysis.

We have also performed statistical analysis on the data and added these in **Supplemental Table 2**. The fluorescent data represent the summary of different endogenous targets that vary in their expression levels. Therefore, comparisons are only meaningful between the same targets. Due to the different fluorescence intensities between targets, we have provided the percentage of GFP positive cells instead of the absolute fluorescence intensities. In addition, we have provided the raw GFP intensity graphs and statistical table in the figure for Reviewer (**revFig.** and **RawGFP-intensity_Reviewer**).

3.      The phenotypical effects of AID induced degradation of endogenous proteins are too simple. Only a morphological picture could not capture the phenotypical consequences of the protein degradations.

Indeed, the phenotypical effects of individual target degradations are going to be more complex than what is presented here. We wish to emphasize that our intention in this study is not to provide significant novel biological insights but rather to use the known/expected biological outcomes as a proofs-of-principle to demonstrate the power of the system for diverse functional studies. We selected targets with robust and clear phenotypical readouts to simplify their functional assessment. The changes selected to be demonstrated are easy to score, and pictures are representative of multiple fields with random selection. The comprehensive characterization of the phenotypical consequences of rapid degradation of multiple targets is out of the scope of the present study and awaits further studies later on.

In summary, I believe the manuscript presented a interesting method that could be interested by researchers using AID in cell lines. However, study was not done and presented in a very rigorous way.

**Second round of review**

**Reviewer 1**

Thank you for addressing my comments.

**Reviewer 2**

The revision addressed all my concerns. I'm pleased recommend accept the paper as this form.
